# Modern venomics—Current insights, novel methods, and future perspectives in biological and applied animal venom research

**DOI:** 10.1093/gigascience/giac048

**Published:** 2022-05-18

**Authors:** Bjoern M von Reumont, Gregor Anderluh, Agostinho Antunes, Naira Ayvazyan, Dimitris Beis, Figen Caliskan, Ana Crnković, Maik Damm, Sebastien Dutertre, Lars Ellgaard, Goran Gajski, Hannah German, Beata Halassy, Benjamin-Florian Hempel, Tim Hucho, Nasit Igci, Maria P Ikonomopoulou, Izhar Karbat, Maria I Klapa, Ivan Koludarov, Jeroen Kool, Tim Lüddecke, Riadh Ben Mansour, Maria Vittoria Modica, Yehu Moran, Ayse Nalbantsoy, María Eugenia Pachón Ibáñez, Alexios Panagiotopoulos, Eitan Reuveny, Javier Sánchez Céspedes, Andy Sombke, Joachim M Surm, Eivind A B Undheim, Aida Verdes, Giulia Zancolli

**Affiliations:** Goethe University Frankfurt, Institute for Cell Biology and Neuroscience, Department for Applied Bioinformatics, 60438 Frankfurt am Main, Germany; LOEWE Centre for Translational Biodiversity Genomics, Senckenberg Frankfurt, Senckenberganlage 25, 60235 Frankfurt, Germany; Justus Liebig University Giessen, Institute for Insectbiotechnology, Heinrich Buff Ring 26-32, 35396 Giessen, Germany; Department of Molecular Biology and Nanobiotechnology, National Institute of Chemistry, 1000 Ljubljana, Slovenia; CIIMAR/CIMAR, Interdisciplinary Centre of Marine and Environmental Research, University of Porto, Terminal de Cruzeiros do Porto de Leixões, Av. General Norton de Matos, s/n, 4450–208 Porto, Portugal; Department of Biology, Faculty of Sciences, University of Porto, Rua do Campo Alegre, 4169-007 Porto, Portugal; Orbeli Institute of Physiology of NAS RA, Orbeli ave. 22, 0028 Yerevan, Armenia; Developmental Biology, Centre for Clinical, Experimental Surgery and Translational Research, Biomedical Research Foundation Academy of Athens, Athens 11527, Greece; Department of Biology, Faculty of Science and Letters, Eskisehir Osmangazi University, TR-26040 Eskisehir, Turkey; Department of Molecular Biology and Nanobiotechnology, National Institute of Chemistry, 1000 Ljubljana, Slovenia; Technische Universität Berlin, Department of Chemistry, Straße des 17. Juni 135, 10623 Berlin, Germany; IBMM, Univ Montpellier, CNRS, ENSCM, 34095 Montpellier, France; Department of Biology, University of Copenhagen, DK-2200 Copenhagen, Denmark; Institute for Medical Research and Occupational Health, Mutagenesis Unit, Ksaverska cesta 2, 10000 Zagreb, Croatia; Amsterdam Institute of Molecular and Life Sciences, Division of BioAnalytical Chemistry, Faculty of Science, Vrije Universiteit Amsterdam, De Boelelaan 1085, 1081HV Amsterdam, The Netherlands; University of Zagreb, Centre for Research and Knowledge Transfer in Biotechnology, Trg Republike Hrvatske 14, 10000 Zagreb, Croatia; BIH Center for Regenerative Therapies BCRT, Charité - Universitätsmedizin Berlin, Augustenburger Platz 1, 13353 Berlin, Germany; Translational Pain Research, Department of Anesthesiology and Intensive Care Medicine, Faculty of Medicine and University Hospital Cologne, University of Cologne, 50931 Cologne, Germany; Nevsehir Haci Bektas Veli University, Faculty of Arts and Sciences, Department of Molecular Biology and Genetics, 50300 Nevsehir, Turkey; Madrid Institute for Advanced Studies in Food, Madrid,E28049, Spain; The University of Queensland, St Lucia, QLD 4072, Australia; Department of Biomolecular Sciences, Weizmann Institute of Science, Rehovot 76100, Israel; Metabolic Engineering and Systems Biology Laboratory, Institute of Chemical Engineering Sciences, Foundation for Research & Technology Hellas (FORTH/ICE-HT), Patras GR-26504, Greece; Justus Liebig University Giessen, Institute for Insectbiotechnology, Heinrich Buff Ring 26-32, 35396 Giessen, Germany; Amsterdam Institute of Molecular and Life Sciences, Division of BioAnalytical Chemistry, Faculty of Science, Vrije Universiteit Amsterdam, De Boelelaan 1085, 1081HV Amsterdam, The Netherlands; LOEWE Centre for Translational Biodiversity Genomics, Senckenberg Frankfurt, Senckenberganlage 25, 60235 Frankfurt, Germany; Department of Bioresources, Fraunhofer Institute for Molecular Biology and Applied Ecology, 35392 Gießen, Germany; Department of Life Sciences, Faculty of Sciences, Gafsa University, Campus Universitaire Siidi Ahmed Zarrouk, 2112 Gafsa, Tunisia; Dept. of Biology and Evolution of Marine Organisms (BEOM), Stazione Zoologica Anton Dohrn, Via Po 25c, I-00198 Roma, Italy; Department of Ecology, Evolution and Behavior, Alexander Silberman Institute of Life Sciences, Faculty of Science, The Hebrew University of Jerusalem, Jerusalem 9190401, Israel; Department of Bioengineering, Faculty of Engineering, Ege University, 35100 Bornova, Izmir, Turkey; Unit of Infectious Diseases, Microbiology, and Preventive Medicine, Virgen del Rocío University Hospital, Institute of Biomedicine of Seville, 41013 Sevilla, Spain; CIBER de Enfermedades Infecciosas, Instituto de Salud Carlos III, Madrid, Spain; Metabolic Engineering and Systems Biology Laboratory, Institute of Chemical Engineering Sciences, Foundation for Research & Technology Hellas (FORTH/ICE-HT), Patras GR-26504, Greece; Animal Biology Division, Department of Biology, University of Patras, Patras, GR-26500, Greece; Department of Biomolecular Sciences, Weizmann Institute of Science, Rehovot 76100, Israel; Unit of Infectious Diseases, Microbiology, and Preventive Medicine, Virgen del Rocío University Hospital, Institute of Biomedicine of Seville, 41013 Sevilla, Spain; CIBER de Enfermedades Infecciosas, Instituto de Salud Carlos III, Madrid, Spain; Department of Evolutionary Biology, University of Vienna, Djerassiplatz 1, 1030 Vienna, Austria; Department of Ecology, Evolution and Behavior, Alexander Silberman Institute of Life Sciences, Faculty of Science, The Hebrew University of Jerusalem, Jerusalem 9190401, Israel; University of Oslo, Centre for Ecological and Evolutionary Synthesis, Postboks 1066 Blindern 0316 Oslo, Norway; Department of Biodiversity and Evolutionary Biology, Museo Nacional de Ciencias Naturales, José Gutiérrez Abascal 2, 28006 Madrid, Spain; Department of Ecology and Evolution, University of Lausanne, 1015 Lausanne, Switzerland; Swiss Institute of Bioinformatics, 1015 Lausanne, Switzerland

**Keywords:** venom, modern venomics, genomics, spatial -omics, evolution, translational research, bioassays, envenomation, antivenom, toxin production

## Abstract

Venoms have evolved >100 times in all major animal groups, and their components, known as toxins, have been fine-tuned over millions of years into highly effective biochemical weapons. There are many outstanding questions on the evolution of toxin arsenals, such as how venom genes originate, how venom contributes to the fitness of venomous species, and which modifications at the genomic, transcriptomic, and protein level drive their evolution. These questions have received particularly little attention outside of snakes, cone snails, spiders, and scorpions. Venom compounds have further become a source of inspiration for translational research using their diverse bioactivities for various applications. We highlight here recent advances and new strategies in modern venomics and discuss how recent technological innovations and multi-omic methods dramatically improve research on venomous animals. The study of genomes and their modifications through CRISPR and knockdown technologies will increase our understanding of how toxins evolve and which functions they have in the different ontogenetic stages during the development of venomous animals. Mass spectrometry imaging combined with spatial transcriptomics, *in situ* hybridization techniques, and modern computer tomography gives us further insights into the spatial distribution of toxins in the venom system and the function of the venom apparatus. All these evolutionary and biological insights contribute to more efficiently identify venom compounds, which can then be synthesized or produced in adapted expression systems to test their bioactivity. Finally, we critically discuss recent agrochemical, pharmaceutical, therapeutic, and diagnostic (so-called translational) aspects of venoms from which humans benefit.

## Background—Why Venoms Matter

**Figure 1:**
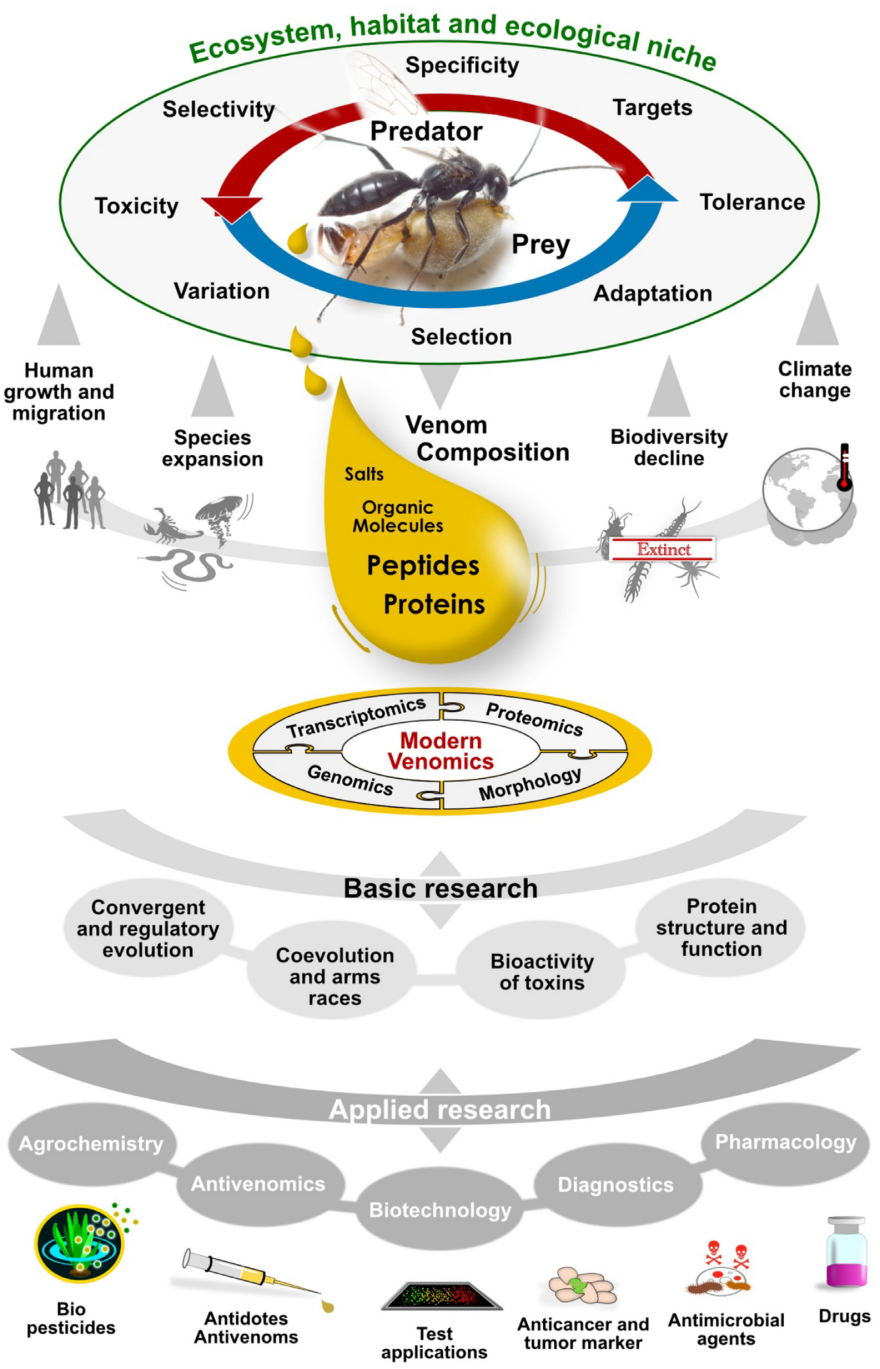
The importance and impact of venom. The biology and ecology of venomous species prompt diversity of venoms, which are constituted of highly specific toxin components that were adaptively produced over time. Predator-prey interactions are major evolutionary forces that often trigger arms races of venom toxicity and resistance. Extrinsic factors that affect venomous species and their interaction with humans include species expansion or decline (linked to the biodiversity crisis and climate change) but also the increasing human growth and migration. Basic venom research investigates why and how venoms and toxin genes evolve based on modern “omics” methods. Translational research exploits these basic studies for developing various applications, ranging from pharmacology (e.g., anti-pain and anti-cancer drugs, diagnostic markers, antivenom development) to agrochemistry (pesticides, antiparasitic compounds for crop and livestock protection) and biotechnology (e.g., nanopore sensing).

Venomous animals fascinate and affect humankind from time immemorial and influence—often unnoticed—many cultural, ecological, and economical aspects of our life [1,2]. Venom is such a successful adaptation that is critical for the fitness of many species, that it has evolved independently >100 times, across all major animal lineages, where it is predominately used for defense or predation [3–5]. Venomous species play key roles in ecological networks in almost all natural habitats. We are just starting to understand many of these relationships, through recent advances in the knowledge of the biology of many venomous animals, the ecological implications of such a complex trait as venom, and its dynamic composition [5–7] (see Fig. 1).

Venom is predominantly used in interspecific interaction, including both predation (such as in spiders, scorpions, centipedes, snakes) and defence (typical examples include bees, sea urchins, and fishes) [5]. In each lineage, venom components are refined—often presumed by arms races—making them highly effective disruptors of physiological processes. The co-evolutionary processes that shape venom diversity and specificity are still being studied, and especially larger, comparative studies are lacking. For few snake species first research results are available that focus on the evolution of venom resistance of rattlesnake prey, which support the arms race theory [8–12]. The remarkable target specificity of many venom compounds stimulated early interest in their potential uses for applied and translational research. As a result, molecules from a few selected taxa such as cone snails, snakes, spiders, and scorpions have been characterized in complex studies aiming at exploring their bioactivity over the course of decades [1,2]. Today, toxins are used in a variety of translational sectors including therapeutics, sustainable bioinsecticides in agrochemistry, and clinical markers in diagnostics [1,2,13–15] (see Fig. 1).

The research field in which all aspects of animal venoms such as evolution, ecology, and translational research, including antivenomics, are integratively studied is modern venomics [16]. Clinical effects of envenomations are frequently untreated because effective and cheap antivenoms, even for the most notorious snakes, spiders, scorpions, and bees, are often lacking. This is one of the urgent humanitarian challenges, especially in countries where envenomations are frequent [17–22]. Moreover, many venomous neobiota that invade new ecosystems facilitated by climate change pose threats not only to humans by increasing envenomations but also to native species and livestock [23,24].

Here we summarize current challenges and approaches on the most relevant theoretical, basic, and applied research disciplines of modern venomics (see Fig. 1). In addition, we highlight future directions and most promising innovations in methods, technology, and platforms that can contribute to animal venom research. The structure of this review reflects the typical workflow of venom studies, from the collection of venomous organisms to applied research, with the aim to be easily used as a blueprint for future venomics studies and a roadmap towards new methodological perspectives.

## Collection of Venomous Organisms

### Taxonomic expertise on venomous animals

**Figure 2:**
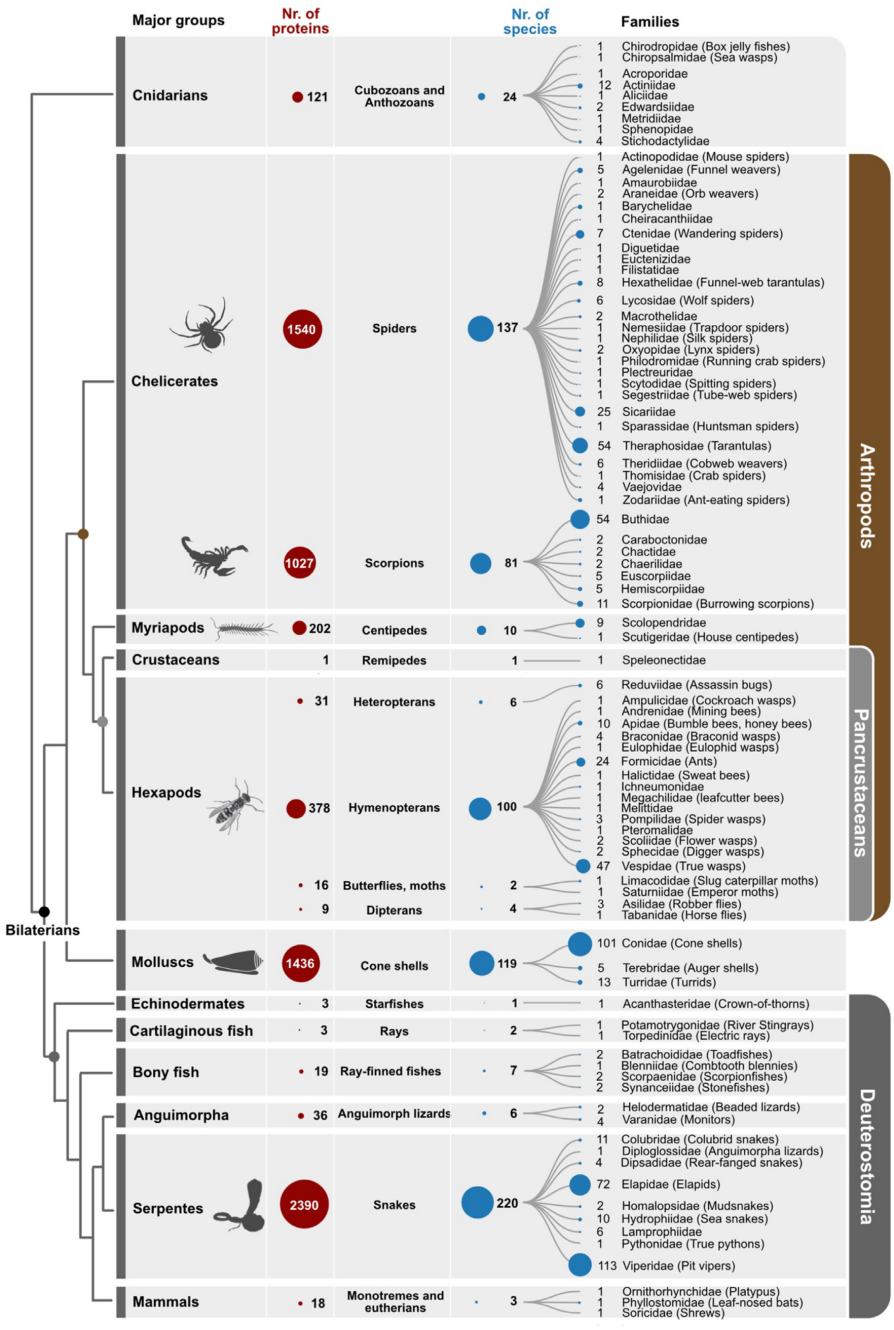
Studied venomous metazoan species and available venom proteins. The numbers of studied venomous metazoans and their reviewed venom proteins that are provided in UniProt's animal venom database (ToxProt) are illustrated. Accessed on 1 April 2022, we mined 7,230 entries for venomous species. Venom protein numbers are only given for the larger taxonomic groups; the red circles are proportional to each other. Only taxa with a described venom protein are included; other metazoan species are pruned. The blue circles that show the species numbers are in proportion to each other.

Most studies on venomous animals start by sampling, identifying, and collecting specimens of venomous species. Until recently, studies almost exclusively focused on taxa that were harmful to humans, such as snakes, spiders, and scorpions, driven partially by the need to mitigate the effects of envenomations [1,5,6] (see Fig. 2). The increasing collection of so far understudied species, particularly invertebrates, raises particular attention to a general, persisting impediment that affects all branches of modern zoology. For many animal groups taxonomic expertise has been declining for decades, precluding the precise assessment of global biodiversity and its trends [25–27]. Understanding the diversity of venomous animals is also crucial to understanding venom diversity and novel protein functions. New strategies to maintain and nurture taxonomic expertise in a biodiversity-driven biodiscovery approach have relevant impact on the field of venomics, especially because venom composition can vary between even closely related species [28].

### Legal collection aspects

A long overdue awareness of equally shared bioresources and responsible collection of species emphasizes old and new legal aspects linked to fieldwork. Naturally, researchers obtain official permissions for fieldwork, depending on collection locality and conservation status of the target species. More demanding, however, are the rather novel rules established by the international agreement on “Access and Benefit Sharing, ABS” of the Convention of Biological Diversity. This agreement aims to standardize a legal framework for the access, transfer, utilization, and benefits of organisms (genetic resources) in a fair and equitable way for the providing country in which samples are collected [29]. The resulting Nagoya protocol is currently enforced in 131 countries worldwide [30], leading on one hand to obvious benefits, such as a legal framework that prevents biopiracy and protects biodiversity, scientists, and traditional medicines in the countries of origin. On the other hand it also implies technocratic hurdles that often hinder collaborative research and in particular translational applications [31–33]. One difficulty is that the rules for knowledge transfer or (financial) benefit from collected organisms (or molecules from these) change from member state to member state if research is published or finally translated into a commercial application. These issues should be more explicitly addressed in the framework of the critical debate around the Nagoya protocol and its implementation.

### Various venom systems require different methods to obtain crude venom

The tremendously diverse venom systems in most animals and the complex anatomy of their venom apparatus [5] require different approaches to collect crude venom. A well-known venom collection method is the milking of front-fanged snakes, where the animals are forced to bite through a thin membrane and release their venom into a clean glass vessel. In contrast, rear-fanged snakes are usually injected with pilocarpine to increase salivation and the released venom is collected manually from the fangs [34]. Similar pilocarpine-based methods have been established for venomous lizards, mammals, and amphibians [34–36].

**Figure 3:**
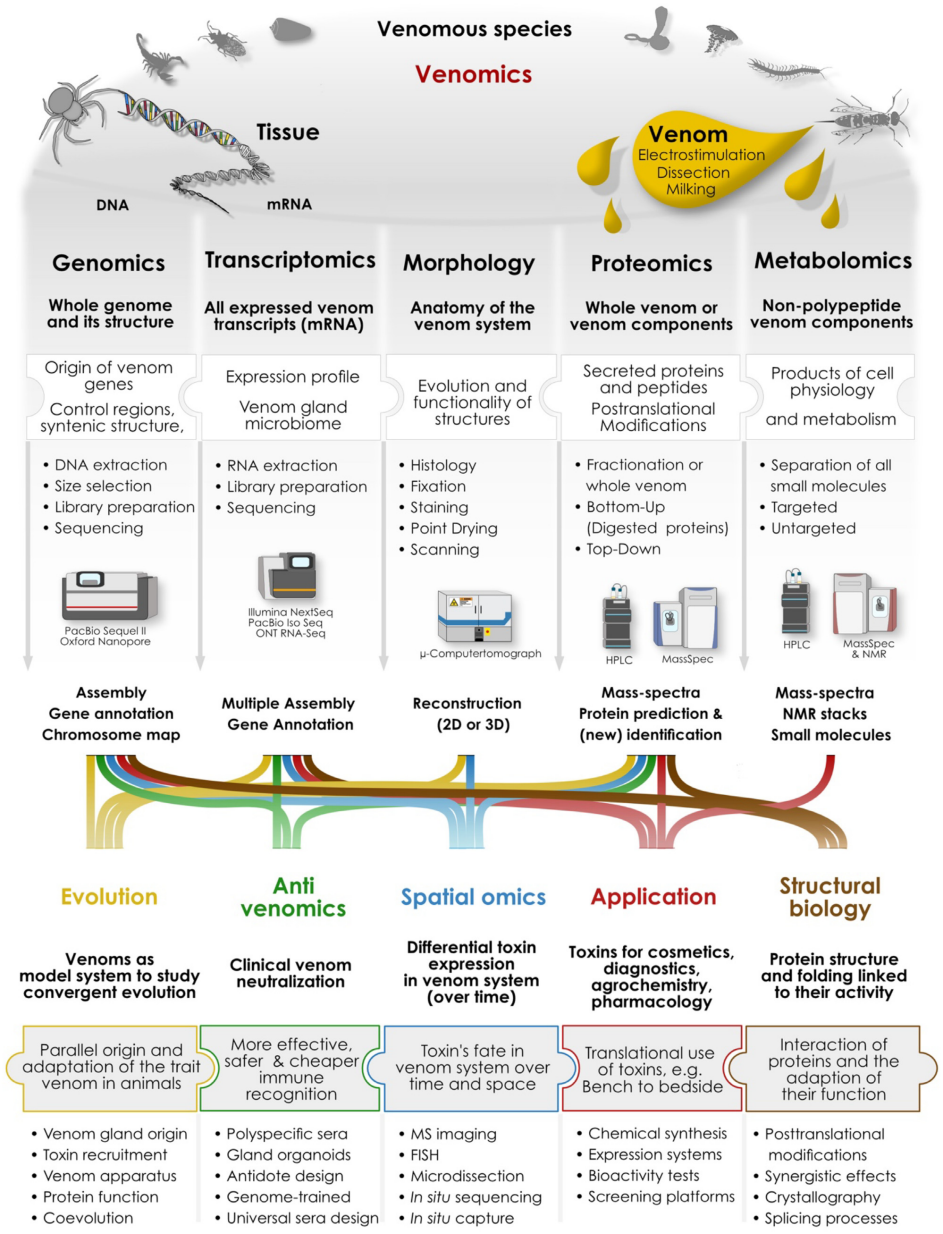
The major interdisciplinary research areas in venomics. The basic, interlinked, modern research fields in venomics are shown in the first row, and linked through simplified workflows with the final output(s). The main applied and evolutionary questions addressed are shown in the bottom, and integrated in the relevant topics. The flow diagrams that connect most research areas with each other illustrate the highly integrative nature of modern venomics. FISH: fluorescence in situ hybridization.

Fish venoms are often extracted from living or frozen specimens by dissecting their venom glands. Many fishes do not have distinct venom glands but clustered, venom-producing, secretory cells that end in a spine groove [37]. For those species, protocols were developed in which crude venoms are extracted through a syringe or by a forced sting into a sponge contained in a tube [38,39]. Chemical extraction from partial- and whole-body samples represents the predominant way of venom collection in many marine invertebrates including echinoderms and several cnidarians [40–43]. For cnidarians, however, alternative protocols that are based on chemically induced discharge have likewise been designed [44]. Cone snail venom can be collected by using live prey as lure or a predator as threat, which stimulates the cones to shoot their venom harpoon into microcentrifuge tubes [45,46]. Venoms of most arthropods such as centipedes, chelicerates, crustaceans, and insects are obtained by electrical, mechanical, or chemical stimulation of venom ejection or dissection of the venom system [47–52]. All these protocols have their pros and cons in terms of convenience and venom yield, but whenever possible, the most “natural” collection method should be preferred. For instance, electrostimulation is known to reveal differing venom profiles compared to manually collected venoms, calling for a cautionary interpretation of putative ecological roles of venoms without the support of further evidence (see, e.g., [53,54]). A comprehensive overview of major venom collection protocols is given in Supplementary Table S1. After collection, obtained samples of crude venom are often pre-filtered from tissue remains, then lyophilized and stored in freezers [34,55] for later analyses (see Fig. 3).

## Venom Metabolomics

### Metabolitic molecules are often neglected

Metabolic profiling of venom refers to targeted and untargeted analysis of its composition of small molecular weight compounds (metabolites). These are sugars, sugar alcohols, sugar phosphates, amino acids, lipids, and nucleotides, covering primary and secondary metabolism intermediates. Small molecules in biological systems act as reactants or products in the metabolic reactions or as signalling molecules for the initiation of certain biological processes and regulatory molecules of protein function. Recent studies have indicated a surprising richness of the venom metabolic profiles across species, which need to be further explored with respect to both its biological role and potential biotechnological impact [56]. So far, venom metabolic profiling has been carried out in a targeted way, searching for molecules that have been known toxins or of potential pharmaceutical interest. However, holistic quantitative analyses of the venom metabolic composition in various species, the parameters affecting it, how the metabolite profile is related to the protein content, and commonalities and differences in the venom metabolic profile between species have not been carried out yet. In this sense, metabolic profiling of venoms is still a rather young research field, requiring further investigation [56]. It is expected that at least some of these metabolites can act as regulatory molecules or direct metabolic intermediates of biological processes in the target species of venom-producing organisms [56–58].

In the case of snake venom, the information about small molecule composition remains largely qualitative rather than quantitative. Only in the past 15 years has the presence of tens to hundreds of small molecules been reported in these venoms [59,60]. Recent studies reported ∼200 metabolites [61] or ∼50 lipids [62] in snake venoms. On the basis of these and upcoming studies, citrate has been the most abundant molecule in snake venom. It has been considered that this is the case in all venoms because citrate can protect the animal from its own toxins. Recent studies in scorpions have added an additional aspect to the high abundance of citrate because the low pH contributes to the high pain that the prey feels from the bite [63]. Moreover, all 20 amino acids have been identified in snake venom, which is another aspect to be further explored. It is expected that the concerted action of many molecules actually affects prey or predator, rather than the specific activity of certain molecules. So far, venom metabolic profiles have been mainly analysed with respect to toxin content, as in the case of acylpolyamines. This group of small neurotoxins with a molecular weight <1 kDa that inhibit glutamatergic synapses are structurally characterized in several spider genera using nuclear magnetic resonance (NMR) spectroscopy and liquid chromatography–tandem mass spectrometry (LC-MS/MS) approaches [64,65], and subsequently identified also in snake venom [60]. It has been postulated that polyamines induce hypotension and direct paralysis, facilitating prey hunting. Following the optimized workflow of these initial studies, Schroeder et al. used an untargeted NMR- and LC-MS/MS–based metabolomics approach for widespread identification of thus far undescribed small molecules in venoms of >70 different spider species [66]. They identified small-molecular polyamines, neurotransmitters, nucleosides, amino acid derivatives, and organic acids. Known and novel low molecular mass compounds from spiders are provided in VenMS, a newly available database [67].

Metabolomics studies have also been carried out on insect venoms. The venom of several species of fire ants (genus *Solenopsis*) contains a characteristic group of piperidine alkaloids [68], in both cis and trans stereoisomers, the trans isomer being dominant as retrieved in gas chromatography–mass spectrometry (GC-MS) [69,70]. More recent studies have been conducted on honeybees [71, 72] and wasps [73] where untargeted and targeted LC–MS(/MS) analyses identified and quantified several organic acids, amines, amino acids, and carbohydrates. Comparison between the venoms among various species and strains of the same species could provide significant insight to the evolution of venom, the actual role of the molecules inside the venom, and the effect of the season, sex, predators, and preys in its composition.

## Proteome Analyses of Crude Venoms

To describe animal venoms, which are predominantly proteinaceous, state-of-the-art mass spectrometry (MS) instruments are used, even for small organisms that deliver minute amounts of venom [54,74]. MS-based venom proteomics are used for (i) general characterization of venom proteomes at the protein family level, (ii) partial or full sequencing of (purified) venom peptides and proteins, (iii) accurate mass determination of peptides and proteins either in crude venom (mass fingerprinting) or after purification, (iv) relative or absolute quantitation of venom proteins and peptides, (v) effective antivenom production (antivenomics), and (vi) 3D structure elucidation by hydrogen deuterium exchange-MS and/or cross-linking MS methods [28, 54, 75–79].

### Advantages and challenges of bottom-up and top-down approaches

In general, the methodological roadmap for any proteomic analysis in venom research is split into 2 major approaches: bottom-up and top-down proteomics [80–82] (Fig. 3). In a bottom-up experiment, intact polypeptides are cleaved by proteases (generally trypsin) and the resulting peptide fragments are analysed by tandem MS. Top-down approaches in contrast describe the native form of venom proteins without any prior degradation. Thereafter, internal fragmentation processes by built-in collision cells of the MS instrument allow for toxin identification, which are well covered in other reviews and therefore not further elaborated here [77,82].

Bottom-up proteomics, achieved by in-solution digestion and direct MS analysis without prior decomplexation (shotgun proteomics), allows for a fast qualitative overview but is hindered by the critical “protein inference problem” that often hinders the differentiation of the numerous toxin isoforms [83]. Therefore, a decisive factor for an extensive quantitative venom analysis involves usually an upstream decomplexation and/or purification (clean-up) of the crude venoms applying several complementary separation methods, either by liquid chromatography (LC), gel electrophoresis, or a combination of both [80]. The existing decomplexation protocols can be adapted to many different instrumental set-ups and provide a detailed quantitative overview to characterize manifold toxin families. Nevertheless, sample preparation is less suitable for high-throughput analyses because it requires large quantities of venom samples and is more prone to contamination that results in false-positive identification of venom peptides [81]. Furthermore, trypsin digestion often prevents the clear identification of different toxin variants, like isoforms, proteoforms, or complex multimer formations [84,85]. To bypass these limitations, a logical step is to eliminate the digestion step and directly analyse intact toxin proteins by tandem MS, in a top-down proteomic approach [86].

In top-down methods crude venom samples are directly loaded to a front-end LC system coupled to the MS instrument. This set-up enables intact toxin mass profiling (MS1) and resolves toxin proteoforms and native posttranslational modifications (PTMs), which are not detectable by bottom-up approaches [86]. To identify the toxin proteins, information by tandem MS (MS2) in data-dependent acquisition mode is acquired. Therefore, a specific peptide ion is delivered for fragmentation to obtain its MS/MS spectrum. The established workflow reduced the needed venom amount as well as operational time, and it is associated with a much lower contamination risk [87]. However, top-down venom proteomics requires a highly specific set-up of high-resolution MS instruments that are only available in specialized laboratories [88]. In the case of high molecular mass toxin proteins, top-down analysis remains challenging and only provides a few observable fragments in tandem MS owing to inefficient ionization by denaturing electrospray ionization (ESI) [89].

### Shortcomings in bottom-up and top-down approaches

Until today, most of the venom proteome studies use one of the well-established bottom-up strategies [54]. A shortcoming of this approach is the bias in protein quantification, arising from many experimental factors, such as instrumental set-up, applied protocols, or databases, which highly affects the protein characterization and prevents quantitative comparison between different studies [90]. This fundamental problem has general validity and also applies to the top-down approach, which is similarly influenced by a number of experimental parameters.

In addition to the various experimental factors, data interpretation and bioinformatic analysis are also important aspects [81,90]. The basic concept for search algorithms fall into 2 broad classes: database-depending and *de novo*. An increasing number of software and packages are now available for peptide/protein identification [91,92]. However, some tools remain challenging for inexperienced end-users owing to lack of appropriate documentation or poor GUIs and show a limited robustness for the output of the same proteomic dataset [93,94]. Experience in handling such proteomic software tools and in partially manual assessment of the data is therefore usually required to properly evaluate the analytical outputs. For all approaches, well-annotated genome and/or transcriptome data are an essential prerequisite to enhance the annotation performance of venom proteomes especially in understudied venomous organisms [95]. Although databases are still limited in terms of taxonomic coverage and do not include species-specific venom protein sequences, close evolutionary relationships within a particular taxonomic group allow the identification of protein families of even totally unexplored venom organisms, reflected by protein sequence homology [54]. However, identifying homologs of venom proteins from totally unexplored venomous taxa with few covered sistergroup species remains difficult and requires a variety of analysed and closely related species, which is possible, e.g., for snakes, but limited for larger taxons such as cnidarians and arthropods.

### Future perspectives for high-throughput venom proteomics

Owing to the limitations summarized above, the current gold standard and good practice for venom proteome analyses consists of application of both complementary proteomic approaches. An overarching future goal for venom proteomics studies is to improve the existing methods to allow faster and even more precise analyses of larger sample sets [54, 86]. A top-down protocol, overcoming some of the aforementioned limitations, was recently developed [96]. This approach enables rapid and detailed profiling of multiple individual venom samples, along with statistical correlation tests for different factors, allowing population-scale analyses for a better understanding of regional and intraspecific venom protein variations.

Nonetheless, for high molecular mass toxin proteins (>30 kDa), current top-down analyses run into technical limits [87,97]. A future application to overcome these limitations in terms of ionization could be native electrospray ionization (nESI). However, native MS requires a specific platform with extended mass range, which is again associated with a loss of speed due to more extensive sample preparation, making this type of analysis still unfavourable for high throughput [98,99].

The application of a hybrid element approach and molecular MS configuration is another powerful concept to decipher venom proteomes in their entirety. The parallel absolute quantification of ultra-high-performance liquid chromatography–separated intact sulfur-containing venom proteins by inductively coupled plasma triple quadrupole MS and ^32^S/^34^S isotope dilution analysis, combined with bottom-up and top-down molecular MS, allows for both the exact quantification and the identification of proteins [79]. Another upcoming MS-based method that offers molecular information on the spatial distribution of toxins and new insights into the biology of venoms, as well as their highly functionalized storage and delivery systems, is further discussed in section “Production of Venom Components.”

## Transcriptome Analyses of the Venom System

**Figure 4:**
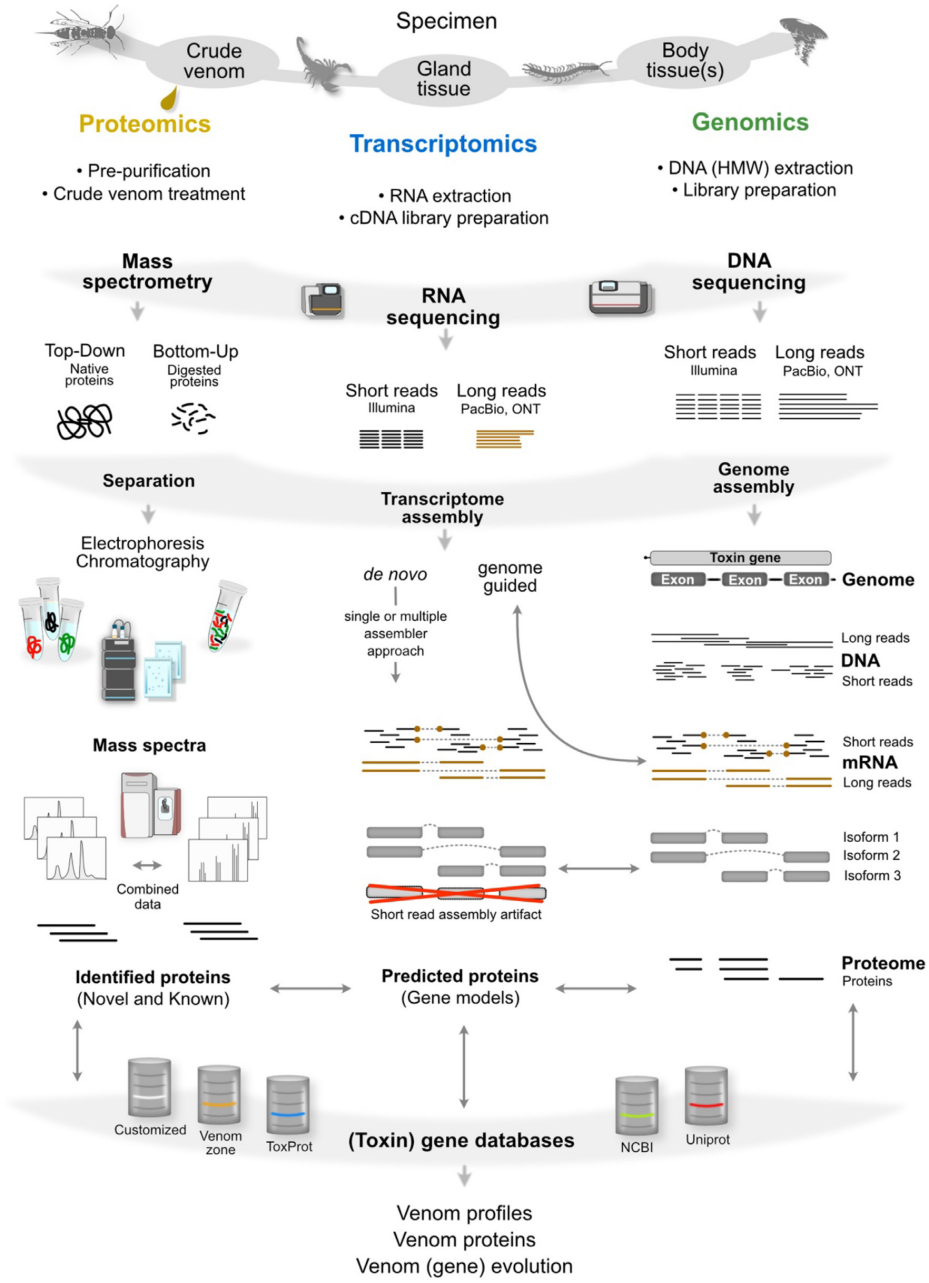
The integration of proteomics, transcriptomics, and genomics in venom research. The general workflow for proteomics is shown on the left. Transcriptome analysis steps are illustrated in the middle. Please note that for state-of-the-art genomics multiple RNA samples from both sexes and different tissues (not only venom glands) are sequenced to perform differential gene expression analyses and to predict gene models more precisely. The genome sequencing steps are condensed and focused on the RNA read mapping. For more details please refer to the references given in the text. cDNA: complementary DNA; HMW: high molecular weight; ONT: Oxford Nanopore Technologies; PacBio: Pacific Biosciences.

The recent advances in high-throughput proteomics to analyse novel venoms are also fostered by the fast development of next-generation nucleic acid sequencing technologies [54,74, 100, 101] (see Fig. 4). *De novo* venom protein analyses, as described above, depend on specific sequence databases of proteins to match masses of native or fragmented (novel) venom proteins. Because many venom proteins, especially of unstudied species, are unknown, high-throughput mRNA sequencing (RNA-seq) of venom glands is often coupled to the proteomics analysis to provide a custom sample-specific database. RNA-seq represents consequently an important and growing core-pillar of venomics to describe the expression of venom genes and proteins even in the smallest venom systems because the required RNA quantities for library preparation range from 100 ng down to 1 µg [102]. However, maybe even more importantly, RNA-seq allows the description of differentially expressed genes in venom-producing tissues, aiding in the identification of putative toxins and their possible origin and evolution from ancestral gene variants in body tissues [103–107]. These aspects are covered in sections “Spatial venomics” and “Significance of genomic data.” Diverse workflows of RNA-seq (also for venomics) have been addressed and reviewed previously [54,100, 108–112].

### Advantages and challenges of transcriptomics

Several venomous animals harbour such minute venom systems that several specimens must be pooled to obtain sufficient amounts of tissue material for RNA-seq. For some particularly small and difficult-to-rear organisms (e.g., remipedes, pseudoscorpions, smaller spiders) the sensitivity of transcriptomics is indeed the last resort to grasp an idea of their supposed venom compositions because crude venom is difficult to obtain [100]. The downside of the sensitivity of modern RNA-seq is that, even if carefully prepared, venom system tissues can be contaminated by other body tissues; in addition, they also contain transcripts of proteins with normal, non-venom-related functions [101]. The best practice is generally to avoid transcriptome-only studies, which should always be integrated with proteomic analyses—a strategy that is now commonly referred to as proteo-transcriptomics [100,101].

For many species a physiological normalization of the venom system, e.g., by milking specimens at the same time to synchronize the replenishment cycle of their gland tissues, is not applicable in the laboratory because milking, rearing, or keeping them alive in the laboratory is difficult [100]. Examples are small solitary bees, marine remipedes, small spiders, marine molluscs and other species. As a consequence, many studies describe venom transcripts and venom gene populations as a snapshot, without the statistical power of differential gene expression analyses with multiple replicates applied in ecological and clinical studies [113,114]. Increasing the sample size of the specimen pool could level heterogeneity by including a larger mix of different “wild-type” venom gland states.

### Novel RNA-seq strategies

The specificity of assembly algorithms implies that diverse assemblers predict venom protein transcripts very differently and that single-assembler approaches might underestimate transcript populations and isoforms [108,111,115,116]. As a consequence, the identification and prediction of proteins via MS might be affected when using these assemblies as specific databases. Recently developed *de novo* assembly packages for short-read data generated by Illumina sequencing platforms thus apply multiple-assembler strategies that combine different assemblers and output a merged assembly [117–119]. New versions even include the annotation steps in the automatized process. One downside is that these programs currently require advanced bioinformatics expertise and often a familiarity with either virtual or physical, often Linux-based, environments, such as Docker or Conda. One future direction is to transform these approaches to more usable mainstream solutions and to link these to genome data to perform genome-guided transcriptome assembly and to foster more hands-on training of venom researchers in bioinformatics. The commercial software packages such as Geneious or CLC Genomic Workbench can cover some bioinformatics aspects. However, they run with expensive subscriptions and are methodologically often less suitable. Henceforward, direct RNA sequencing with novel sequencing platforms, such as ONT Nanopore or PacBio IsoSeq, with long reads and improved accuracy, will be increasingly applied, minimizing artificial transcripts or gene predictions [110,120].

The sequenced snapshots of expressed mRNA protein precursor molecules from tissue of the venom systems reveal not only transcripts of toxins and other venom proteins but identify as well house-keeping genes that assist venom secretion. As a consequence, RNA-seq of multiple tissues (differential gene expression) in combination with genome data and spatial -omics techniques is an important tool to reconstruct cellular pathways and mechanisms through which venom proteins and toxins are processed and translated [110]. A future direction will be to apply single-cell RNA-seq (scRNA-seq) methods to differentiate expressed toxins in diverse gland cell populations to reveal spatial and temporal venom variations [114,121, 122]. Single-cell transcriptomics has been successfully applied in general to a variety of diverse animals including sponges, ctenophores, placozoans, cnidarians, planarians, nematodes, arthropods, ascidians, and vertebrates (see, e.g., [123]). This breakthrough method simultaneously measures gene expression from thousands of individual cells. Clustering cells that share similar expression profiles allows for the identification and characterization of cell types that can be even more nuanced than traditional morphological characterizations. Cnidarians are a phylum typified by their venom-producing cells called nematocytes, whose biochemical and structural components have been successfully identified using scRNA-seq analysis [123–126]. Not only is this method capable of answering essential biological questions related to venom, but it is also likely capable of being implemented in non-model organisms and should be further tested in other venomous taxa. Unlike the use of transgenics to generate reporter lines and then sorting positive cells to generate a cell type–specific transcriptome, virtually any non-model organism can now be explored at a cellular resolution. Beyond mRNA, other techniques can reveal genomic features at the cellular level. For example, ATAC-seq sequences portions of DNA to assess genome-wide chromatin accessibility and identifies key gene regulation mechanisms such as transcription factor binding sites [127]. Because ATAC-seq is highly sensitive it requires only minute amounts of chromatin and it can be used to sequence even single cells, allowing the integration of transcriptomics and epigenomics at cellular resolution [128]. Such insights into the gene regulation of venom-secreting cells are essential to understand the evolution and development of venom systems (see also section “Critical and future aspects on current bioassays”). A recent study on rattlesnakes used this (in venomics) relatively new approach to reveal that a complex genotype underlies a simple venom phenotype [129].

### Future perspectives of proteo-transcriptomics

Future directions for further developments of proteo-transcriptomics consist mainly in the development of more sophisticated and user-friendly data analysis strategies in combined, integrative interfaces. The most comprehensively assembled transcript libraries are generated with multiple-assembler pipelines including long-read RNA-seq data and are ideally guided by available genome data. The predicted gene models and annotated protein genes are then used to identify proteins, using the outputs of MS approaches in which bottom-up and top-down methods are applied to fragmented and native protein samples (see Fig. 4). An even more holistic design is achieved if complementary spatial transcriptomics (ST) and mass spectroscopy imaging (MSI) methods are applied (see section “Spatial venomics: non-targeted, high-throughput methods to visualize toxins”).

## Integrating Molecular Venomics with Functional Morphology

### Challenges in connecting morphology, function, and molecular data

Beyond classical compositional and structural toxin analyses by proteo-transcriptomic approaches, in recent years there has been a steadily growing interest in the connection of these data to morphology, to elucidate the localization and mechanisms of venom toxin production, storage, and delivery [36,130]. Obtaining information on the morphological aspects of venom systems provides mechanistic insight into how and where venom is produced and expelled, which is often not in a uniform manner [45, 122, 130, 131]. Thus the morphology of venom systems is crucial to understanding venom function [5]. Furthermore, integration of morphological and molecular aspects of a venom system—e.g., through information on the spatial distribution of toxins—can provide an important functional context for understanding both toxin function [45,132] and evolution [45,130], as well as the intricacies of the venom system itself [133].

Venom gland morphology is extremely variable: glands with a pronounced secretory function can have different numbers of cells, shapes and secretory modes [134]. Unicellular glands are mostly located in the epithelium of, e.g., aquatic vertebrates, annelids, and molluscs. Multicellular glands are usually located beneath the epithelium. In terms of shape, glands can be defined as globular (acinous) or tubular. Composite glands can result from the association of several acinous and/or tubular glands [134]. Below the cuticle of arthropods, sunken uni- or multicellular glands are present that possess a specialized canal cell, which develops a conducting canal lined by a cuticle [135]. In terms of secretion mode, 3 types can be distinguished. In apocrine secretion, secretory grana or a liquid secretion is released. However, this secretion also contains organelles and mitochondrial as well as nuclear proteins [136]. In merocrine secretion, parts of the gland cells are released with the secretion. In holocrine secretion, the whole cell is released (e.g., in mammal sebaceous glands). Because the loss of cell material in merocrine and apparently holocrine secretion is large, regenerative cells are present, e.g., in cnidocytes of Cnidaria or in the midgut of insects [134]. Thus, in different animal taxa glandular structures can range from single cells to large composite glands. Visualization as well as anatomical analysis methods have to be chosen according to the level of interest, ranging from ultrathin sectioning to analyse subcellular anatomy to micro-CT analysis to visualize general gland morphology [137,138]. Novel technological innovations towards 4D tomography, which includes dynamic data from samples that undergo change during scanning, might enhance our functional understanding of venom systems [139]. Nevertheless, integration of molecular data in the context of morphological or functional aspects is still challenging, and the classical venomics approaches (proteomics, transcriptomics, genomics), used to examine spatial information of toxin production in various insect-feeding species, only allow limited resolution [5,122, 140]. The glandular origin of the venoms in these studies was investigated by dissecting the secretory portions of the venom apparatus into a series of multiple segments and analysing respective sections by proteo-transcriptomic methods for variable toxin profiles [141–144]. Although macrodissection of venom glandular apparatus gives new insights into the biology of venoms, it has several drawbacks including the laborious preparation, low resolution, loss of morphological structures, and averaging effects across the section samples.

### Targeted methods for the inference of spatial toxin distribution

As outlined above, the difficulties derived from the intrinsic nature of some venomous organisms and from the technological limitations of most commonly applied analysis methods have hampered a comprehensive integration of functional, morphological, and molecular data. To obtain molecular information on the subcellular level, techniques such as in situ hybridization and immunocytochemistry have been used to map the spatial distribution of toxins and venom components directly on tissue sections [145,146]. These methods have demonstrated great potential in venom research, for instance, revealing previously unknown parts of the venom apparatus [133] or heterogeneity of toxin expression in venom glands [121,147]. However, these techniques allow only a few previously knowSTn targets to be mapped simultaneously, providing limited molecular information.

### Spatial venomics: non-targeted, high-throughput methods to visualize toxins

Advances in imaging technologies, proteomic analyses, and high-throughput sequencing have facilitated the development of non-targeted techniques, such as MSI and ST, termed under the name “spatial venomics.” MSI has become popular in recent years and as a non-targeted approach, which is ideally suited to interrogate the spatial distribution of multiple toxin proteins, peptides, or small molecules without prior knowledge of their identities [54]. The spatial resolution for different MSI instrumentation spans several orders of magnitude from 1 mm to 30 nm [148]. While several modes of ionization exist, matrix-assisted laser desorption/ionization (MALDI) remains the most appropriate for mapping proteinaceous toxins within venom gland systems. To date MSI has been used to explore the distribution of venom components in a variety of venomous organisms including cnidarians, arthropods, and reptiles [130,149–152]. The MSI workflow in all studies acquires individual mass spectra in a regular raster (usually ∼50 µm) across venom gland sections, which allows their distribution to be displayed on the basis of single toxins in a 2D density map. Recently, a new approach, named functional MSI (fMSI), allowed indirect detection of phospholipase A_2_ (PLA_2_) proteins by on-tissue enzymatic activity screening, which underlines the great potential of MSI for future *in situ* approaches [150,153].

ST is a novel technology that allows the visualization and quantitative analysis of whole transcriptomes, creating gene expression maps within individual histological sections [154,155]. Tissue cryosections are placed on glass slides that contain an array of poly-T capture probes uniquely identified by spatial barcodes that allow the determination of the origin of each mRNA molecule within the tissue. Therefore, ST allows the generation of complementary DNA (cDNA) libraries with accurate positional information for RNA-seq, adding a spatial dimension to transcriptome data that enables analyses of gene expression within a morphological context. It is thus an ideal technique to investigate poorly known or challenging venomous organisms because it allows the identification of toxin genes and their spatial expression patterns within the tissue, thus simultaneously characterizing the molecular composition of the venom and the morphological and functional organization of the venom-producing tissue. Additionally, the sensitivity and high spatial resolution of up to 55 µm (equivalent to 5–10 cells) of the ST array allows the study of very small venomous organisms while circumventing common obstacles encountered in bulk RNA-seq differential gene expression analyses. For instance, ST eliminates the need to pool small specimens, losing the statistical power of biological replicates, and avoids contamination from tissues not related to venom production [100,101]. Furthermore, ST can also be combined with single-cell RNA-seq [156], offering the possibility of simultaneously identifying different venom secretory cell types and their specific spatial location in the venom system.

These spatial non-targeted methods allow us to conduct data-driven exploratory analyses without preselecting known targets of interest and are excellent tools to investigate cell types and tissues whose organization and functions are not well understood [157], such as many animal venom systems. These 2 technologies add a spatial dimension to venomics, revealing genes and proteins associated with morphological features, providing essential functional information about venom systems, from the genetic to the phenotypic level, from the molecular composition of the venom to the morphological features of the delivery system.

## The Significance of Genomic Data

Despite the increasing availability of technologies for generating high-quality genomes, venomous animals are still under-represented in most databases and studies. In particular, comparative genomics studies on the origin and evolution of venoms are very sparse [103]. A major barrier that hinders comparative genomics is the reduced quantity of material obtainable from very small venomous organisms. However, developments in (ultra) low-input protocols may aid in overcoming this hurdle, using amplification techniques. Novel methodologies also allow sequencing of difficult genomes of predominantly small marine invertebrates (e.g., nematodes, molluscs, and others) that are characterized by extensive production of mucus (Mucopolysaccharides) and/or other inhibitory molecules (polyphenolic proteins) [158, 159]. These new methodologies are being exploited by a number of genome consortia that are connected under the umbrella of the Earth Biogenome Project [160], whose ultimate goal is to sequence, within the next decades, the genomes of all animal and plant species to better understand their evolution, ecology, adaptations, and interconnections, and to safeguard—as last resort digitally—the threatened biodiversity and bioresources on earth [160,161, 161]. Linked to these efforts, the numbers of published high-quality, chromosome-level genomes have already substantially increased, allowing for more comprehensive investigation of the origin and evolution of venom genes, predominantly from iconic groups such as snakes, spiders, and cone snails [129,162–171].

### Venom gene origin

**Figure 5:**
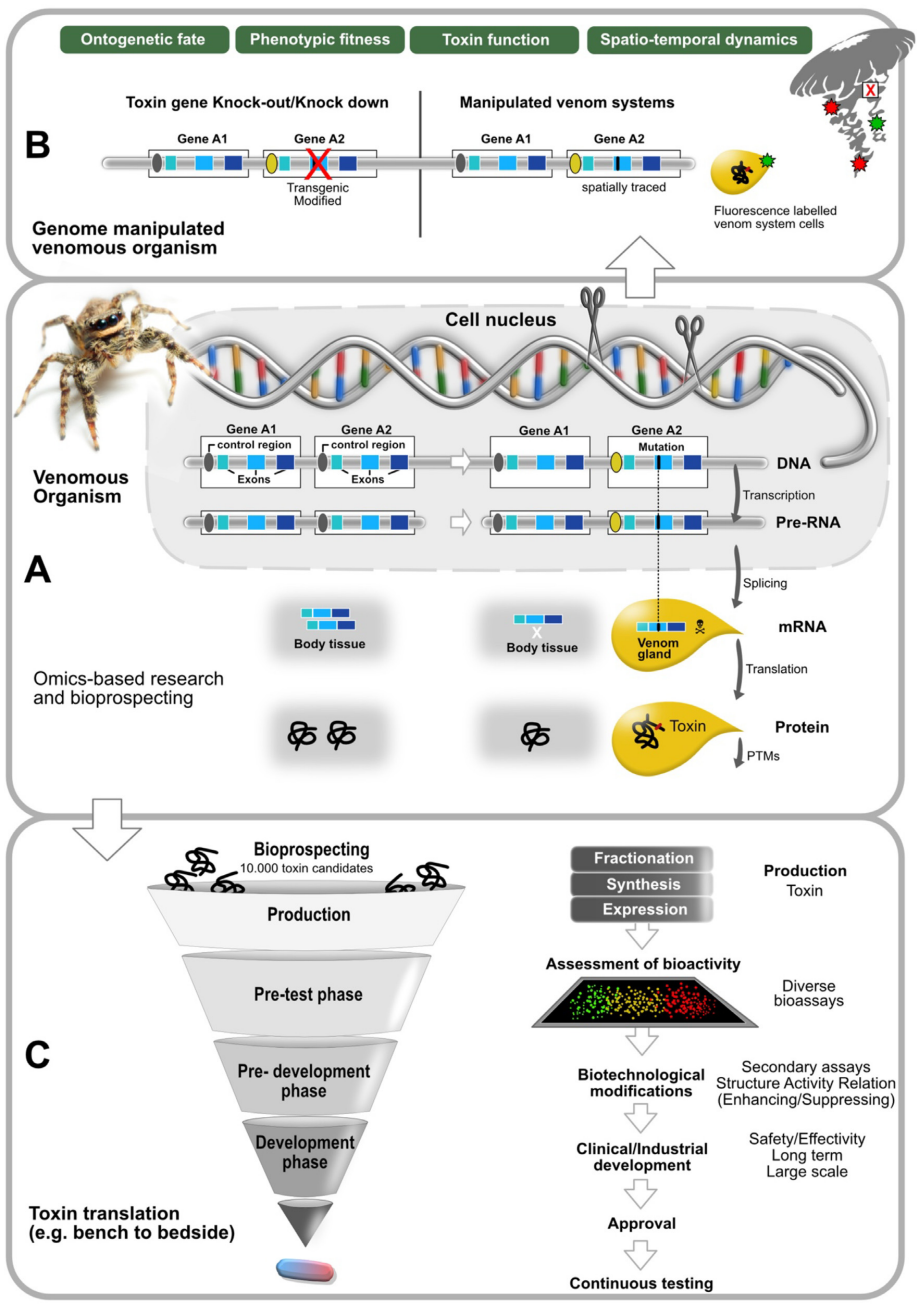
The integration of -omics-based research to improve translational research but also our basic understanding of venom and toxin gene evolution. (A) Shows the biological process from gene to protein; (B) illustrates genome editing aspects to investigate toxin evolution, function, adaptive value, spatio-temporal variability, and ontogenetic fate; (C) summarizes the major steps in translational research, from bioprospecting to application. PTMs: posttranslational modifications.

Genome data are an important reference material to assess the accuracy of transcripts and gene models obtained from RNA-seq data by genome-guided transcriptome assembly approaches. However, high-quality genomic data with good gene annotations are only obtained if multiple tissue samples are mapped on the genome and transcript-based gene predictions, improved by corresponding proteome data, are implemented [103,104] (see Fig. 4). Many available genomes lack a reliable gene annotation because they were automatically annotated [161,172]. Annotation with automated pipelines is prone to both false-positive and false-negative matches because venom genes belong in most cases to multi-gene families, often with high similarity of new toxin copies to their ancestral non-venom-related paralogs [173]. Genomic data are likewise of utmost importance for identifying ortholog genes (especially when short) and for comparing venom genes to their non-toxic homologs in individual genomes [174–176]. One future challenge is to improve the reliability and the speed of the process to predict genes in genomes. Without knowledge of the physical genomic location of a toxin-encoding gene, it is very difficult to identify its orthologs in other species. Genomes also provide information on exons and introns that is crucial to gene structure evolution (Fig. 5A). For instance, gene duplication often results in incomplete sets of exons that can be used to trace back duplication events [165,173] that are impossible to detect otherwise [177]. It has been shown recently that some genomic studies have made erroneous assumptions by overlooking orphan exons [174,177]. At the same time, intronic sequences can provide a more reliable phylogenetic signal when genes evolve under extremely strong positive selection [178]. For example, sometimes a toxin-encoding gene can evolve from a non-toxic gene by deletion or gaining of exons [179].

The evolutionary history of toxin genes is more realistically reconstructed if their exact genomic location is identified using unrelated, syntenically conserved flanking genes, followed by location of that same genomic region in the outgroup species’ genomes. After that, an exon screening (via BLAST or other sequence similarity tools) should take place to locate all related genes and pseudogenes in that region. A phylogenetic analysis of complete gene sequences subsequently helps to identify gene subclades. Previous knowledge of gene evolution can help to infer the most likely evolutionary history of a given gene [177,180–183]. Several helpful online and standalone software tools have been recently developed (e.g., SimpleSynteny, SynMap, AliTV [184–186]), however, they often rely on previously published *de novo* genomic annotations, which as explained above are particularly error-prone [172]. One direction for improvement of this step is to train the gene prediction with specific proteo-transcriptomic data from venom proteins (e.g., [104]. With the aforementioned genome sequencing initiatives we will soon be able to apply comparative genomics methods to detect the occurrence of convergent venom gene evolution in larger clades: the inclusion of many non-typical venom taxa is in fact crucial to infer general and lineage-specific patterns of gene evolution.

### Venom gene manipulation by knockdown and CRISPR

Advancements in available tools and techniques for genetic manipulations are currently increasing among non-model species, including venomous animals. For instance, parental and embryonic RNA interference (RNAi) are regularly used to investigate the developmental biology of the common house spider, *Parasteatoda tepidariorum* [187,188]. More advanced CRISPR-mediated mutagenesis has also been developed for some model venomous species, e.g., the jewel wasp, *Nasonia vitripennis* [189]; the honeybee, *Apis mellifera* [190]; and the red imported fire ant, *Solenopsis invicta* [191]. However, it is only in the cnidarian *Nematostella vectensis* that genetic manipulations, including knockdowns using morpholinos and short hairpin RNA, as well as CRISPR-mediated techniques [192, 193], have been used to address venom-related questions, such as elucidating the factors associated with the biogenesis of venom-secreting cells [122,131] (see Fig. 5B).

Transgenic approaches in *N. vectensis* have allowed the tracing of spatiotemporal dynamics as well as the localization of 2 distinct venom-secreting cells (nematocysts and gland cells [194,195]). Furthermore, specific toxins were found to be secreted in subpopulations of both cell types [194], adding a new level of complexity lacking in previous analyses. Furthermore, by incorporating fluorescent markers into the structural components of venom-secreting cells using CRISPR/Cas9 techniques followed by FACS sorting of different types of nematocytes, different types of stinging cells were isolated [196]. RNA sequencing of the isolated cells revealed numerous differentially expressed genes, including some transcription factors resulting from lineage-specific duplication and essential for proper cnidocyte differentiation [196].

While these techniques have been instrumental in elucidating the structural components of the venom system, the characteristics of toxin components remain largely unresolved. Particularly of interest is the ability to genetically manipulate toxin-encoding genes in animals and test the impact on the fitness of mutants. Examples of such studies may include the deletion of a functional toxin before exposing the mutant to native predators and prey to test whether defense and predation abilities are affected.

A further expansion of this approach would be deleting multiple different toxins followed by subsequent mutants’ crossings to produce individuals completely lacking venom. Additional assays could include knock into the animal's genome (“gene knockin”) additional toxin domains, to cause overexpression of a toxin, or introduce precise modifications of single nucleotides to recapitulate an ancestral venom profile. The recent development of organoids from snake venom glands represents a new opportunity to test *in vitro* genetic manipulations [121]. Although this technology will need further developments to be easily applied to other systems, it may provide opportunities to simultaneously knock down toxin genes or edit regulatory regions to perform functional studies.

## Production of Venom Components

### Challenges of isolation-based venom biodiscovery

Functional characterization of toxins isolated from venom can be conducted directly using the purified peptide or protein. However, the small size of many venomous species, in particular invertebrates, hinders the mechanical manipulation of the venom system and/or the collection of a sufficient amount of venom from a single specimen [6]. In these cases, an extraordinarily large number of specimens must be sampled to accumulate sufficient venom for the isolation of single compounds, raising ethical concerns [6, 100,197]. In these and other cases where toxin sequences can be identified only *in silico* (transcriptome or genome-based, see Fig. 4 and Section: Challenges in connecting morphology, function, and molecular data), methods of chemical synthesis and recombinant expression, which can produce milligram amounts of single venom components, are becoming increasingly important (Fig. 5).

### Chemical synthesis

Chemical synthesis is ideally suited for relatively short peptides (<50 residues) and requires prior knowledge about the peptide sequence, which must be obtained by MS or other methods (Edman degradation, novel NMR-based methods) on the isolated natural toxin or from genome or transcriptome sequencing. In many cases, additional knowledge about the disulfide pattern (as well as other PTMs) is required to ensure the correct native folding of the produced toxin.

While not suited for larger venom proteins, this approach has been instrumental in the functional and structural characterization of toxin peptides. The most common method applied is solid-phase peptide synthesis, which can produce quite large amounts of peptide (generally milligrams or grams, but kilogram yields are possible in industrial settings). Advantages of chemical synthesis include the ability to incorporate, e.g., unnatural amino acids, D-amino acids, reporter groups, and unusual PTMs (such as brominated tryptophan) that are impossible to produce by recombinant production, as well as the regio-selective formation of disulfide bonds and cyclization [199].

### Recombinant production

With an increasing number of both prokaryotic and eukaryotic expression systems that support the production of post-translationally modified proteins, recombinant production of toxins is also becoming more accessible. Given that most toxins are endogenously produced in the endoplasmic reticulum of the host and that PTMs can play crucial roles for toxin activity [200–202], eukaryotic host cells for recombinant production generally provide the best chance of producing functional toxins. Most common eukaryotic host systems used for toxin production include the yeast *Pichia pastoris*, insect cells, and a variety of mammalian cell lines such as HEK293 and CHO. Although prokaryotic, the bacterial host *Escherichia coli* has been used to express thousands of toxins [203,204] and can yield milligram amounts of toxin in a standard laboratory setting. However, it is prone to a major drawback, the inability to add common PTMs such as glycosylation, C-terminal amidation, and hydroxylation. The availability of a variety of systems, including specialized strains, can allow for the production of disulfide-bound toxins in *E. coli* (see below). Regardless of the specific expression host, recombinant expression offers the advantage of incorporation of affinity purification tags and the ability to easily produce a large number of variants for functional testing and to obtain proteins that are substantially larger than those commonly made by chemical synthesis.

### Future perspectives of toxin production

*In vitro* refolding of chemically synthesized peptides is often inefficient, especially for peptides containing ≥3 disulfide bridges. On the basis of sequence homology with characterized toxins, the disulfide pattern may well be deduced and thus allow for directed folding strategies. However, for novel, previously uncharacterized sequences, recombinant production may be the best suited or unique option, and as such has been subject to remarkable developments in recent years. To allow disulfide bond formation in *E. coli*, a variety of methods and strains already exist and many have been used for toxin production [205–210]. Recently, new systems have been introduced [211] and the ability to produce thousands of disulfide-bonded animal toxins in *E. coli* has been demonstrated in important studies from the Vincentelli lab [204,212,213]. Hundreds of cystine-dense peptides containing ≤5 disulfide bonds have recently been produced in HEK293 for, e.g., structural characterization [214]. Moreover, the same expression system has been used for surface display, which allowed screening of thousands of toxin peptide sequences to identify strong peptide interactors for specific targets [215]. This work demonstrated the potential of cystine-dense peptides to function as binders for transmembrane targets that are otherwise difficult to inhibit. Finally, cell-free synthesis approaches that have been used sporadically to produce venom toxins [216,217] have a great potential for further developments. It is imperative to scale up such techniques to fulfil the potential of the many new sequences now available. The infrastructure needed to produce thousands of peptides is often not available in a typical (academic) laboratory setting. Such undertakings will therefore require larger publicly funded consortia and/or closer collaborations between academia and industry than currently exist.

## Applied and Translational Venom Research

After sufficient quantities of crude venom or venom fractions are obtained and/or suitable amounts of a single compound are produced (see sections “Collection of Venomous Organisms” ) their applicative potential can be assessed through a variety of bioassays. This translational perspective of animal venoms has always been a major driver of venom research. However, a large gap remains between the plethora of described animal venom compounds and the much fewer approved drugs, bioinsecticides, and pharmacological and cosmeceutical products based on animal toxins [2,15]. Most candidates are dropped during the trial phase, while the few remaining ones have to be adapted biotechnologically to enhance or improve their properties. Both the testing and the optimization steps are expensive and time-consuming, being a major hurdle for applicative developments. The insights from the aforementioned -omics methods now allow a far more efficient and targeted bioprospecting that also increases the chances of realistically identifying promising candidates and reducing the number of unsuccessful candidates. An equally important aspect of bioassays is that they allow interactions between predator and prey to be tested, e.g., venom resistance and ecological factors such as differing prey that might influence venom complexity [140]. However, in this section we focus now predominantly on applied aspects of bioassays.

### Bioassays in pharmacology

**Figure 6.**
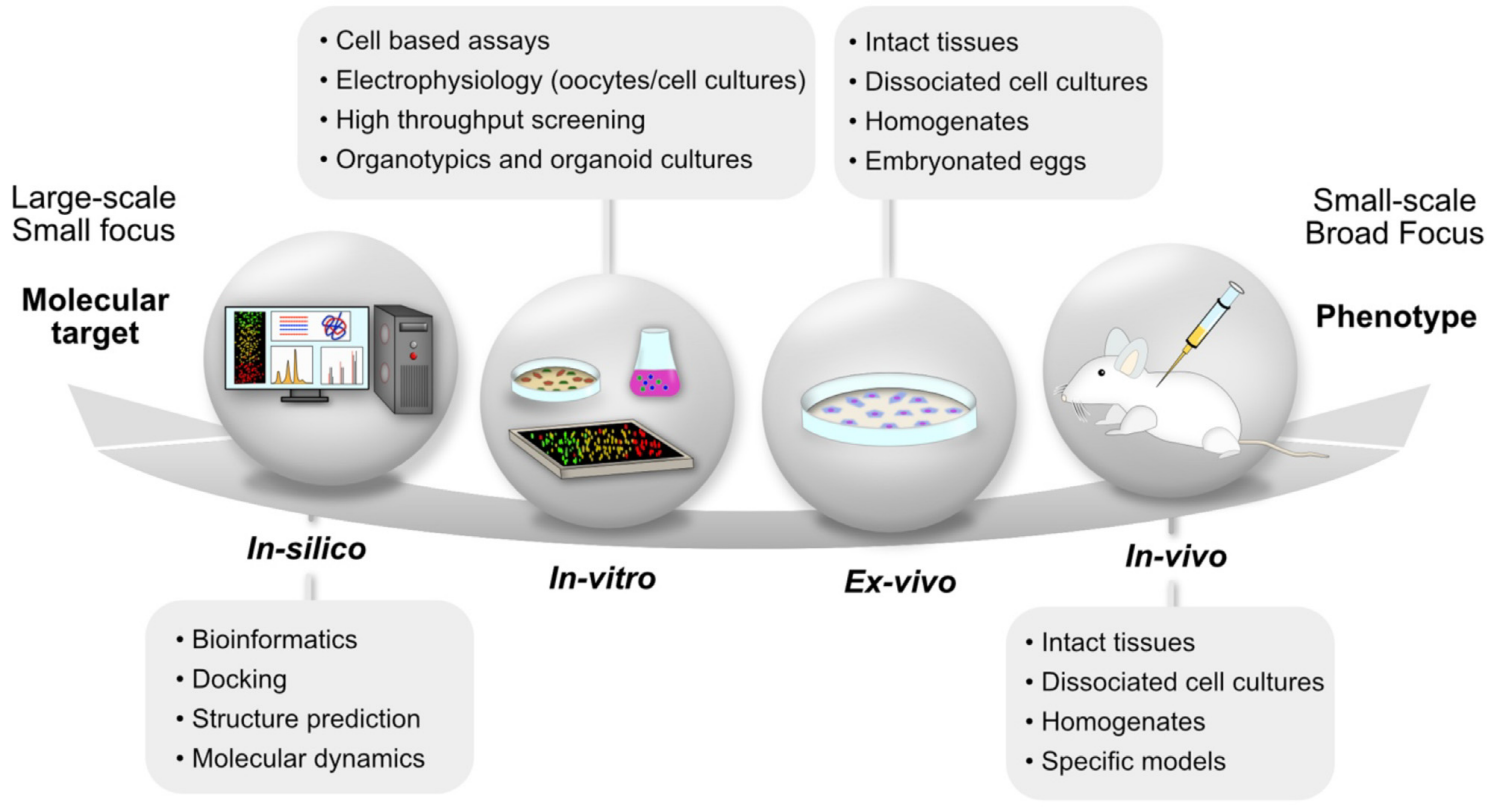
Approaches to study the activity of venom components span from *in vivo, ex vivo*, and *in vitro* to *in silico* methods. This allows the characterization of a broad spectrum of physiological effects, from whole-organism phenotype to molecular target.

Bioactivity assay systems have been developed over many years to characterize the mechanism of action and pharmacological properties of venoms and have been constantly improved to reveal novel targets [218–220]. In particular, the complexity of venoms is mirrored by an ever-increasing number of bioassays developed to characterize their structural, functional, and pharmacological properties. These bioassays span from *in vivo* phenotypic screens to *ex vivo* and *in vitro* models, as well as *in silico* analyses (Fig. 6). These approaches are mostly pursued from 2 complementary perspectives: (i) Basic biological characterization of a given venom leads to description of behavioural phenotypes and the identification of underlying cellular and molecular targets. (ii) Target-specific assays may be used to identify venoms and toxins interacting with the molecular target(s) of interest [221,222].

Injection into an organism *in vivo* can mimic many aspects of naturally occurring bites or stings, thereby reflecting the complexity of physiological and behavioural phenotypes [220]. To understand ecological interaction *in vivo* assays are thus an important tool, despite the drawback that bioactivity of single components is masked by the use of whole venom [140]. Whole-organism phenotypes and *in vivo* assays offer a particularly powerful approach when combined with the use of transgenic animal and transient knockdown/expression systems to test putative mechanisms of actions of a venom [194,223].

***In vivo* assays**

Despite *in vivo* assays on vertebrates posing ethical limitations, being labour intensive, and often not being scalable to high-throughput approaches [222], murine models are widely used in basic and applied venom research for the characterization of effective and/or lethal doses (ED_50_, LD_50_). *In vivo* assays remain especially important to better understand arms races between predator toxicity and natural prey resistance [224], which has especially been described in more detail for rattlesnakes and their prey, such as squirrels [8,9,225,226]. The effects of venom from jellyfish (*Chrysaora sp*.) were, e.g., analysed in zebrafish (*Danio regio*) as one of the toxinological model organisms, which revealed toxicological mechanisms, such as hemorrhagin in eyes and hyperpleasia or hypertrophy on other organs [227]. *In vivo* assays also remain a prerequisite for the clearance of novel therapeutic agents by most regulatory agencies [228]. High-throughput chemical screens are performed using zebrafish embryos and chemical libraries, taking advantage of several available transgenic lines and disease models. Other potential organisms such as *Drosophila* have also been used in venom-based research with promising outcomes [229].

***Ex vivo* assays**

The complexity of *in vivo* approaches can be reduced by experimenting on representative *ex vivo* tissues. Tissues extracted from mice, frogs, electric eels, chicken, and other organisms were instrumental for laying some of the most fundamental cornerstones of basic physiology studies of venom research [222]. *Ex vivo* methods reduce and refine animal use because multiple samples can be established from a single killed animal. In addition, they offer a more precise control of experimental conditions, allow convenient access for microscopy and biophysical probes, and facilitate the study of venom-induced effects on specific cell types even on subcellular structures and organelles. Similar to *in vivo*, transgenic or transient transfection/transduction may help to elucidate molecular mechanisms of action. However, freshly isolated tissues from animals may not be well suited for high-throughput analysis or for studying the toxin function at a single-protein level [222,223].

***In vitro* assays**

Broad functional studies can be performed *in vitro* in cell lines, which allows precise system compositions, efficient pharmacological access, and genetic manipulation for knockout/down, knockin, and mutagenesis, as well as the use of reporter systems [230, 231]. Immortalized cell lines provide valuable insights into the therapeutic potentials of animal venoms and their components as drug candidates. In addition, primary cells obtained from oocytes of the South African clawed frog *Xenopus laevis* allow exogenous expression of functional ion channels for electrophysiological analysis to evaluate their interactions with venoms and toxins [232,233]. Miniaturization and sensitivity are ever increased to characterize minimal amounts of venom components. Multiwell-plate assays have facilitated high-throughput screening of venom components against cells, enzymes, receptors, and ion channels, many of which are approved drug targets [222,234]. *In vitro* biophysical methods offer the ability to manipulate both toxins and receptors at a molecular level and record the resulting effects with high spatial and temporal precision [235]. *In vitro* studies of venom cannot replace *in vivo* experimentation owing to the inability to reflect the full physiological complexity; however, they are important to reduce and refine animal use by providing mechanistic insights to allow focused and informative *in vivo* experiments [236].

***In silico* assays**

Modelling of toxin interaction with cell-membrane receptors *in silico* has emerged as a powerful novel approach for drug discovery [237] that requires detailed structural information of both the toxin and its receptor protein. Structures of numerous toxins derived from animal venom were determined using X-ray crystallography or NMR spectroscopy in the 1970s and 1980s [238,239]. In contrast, nearly 80% of all membrane proteins with known structures were determined only in the past decade, owing to the “resolution revolution” in electron-microscopy technology and the development of advanced crystallographic techniques [240], which has provided numerous structures of venom peptides in complex with their cell-membrane receptors [241–244]. Atomistic simulations of toxin-receptor interactions currently rely on 2 complementary methods, namely, docking and molecular dynamics [245], which may provide realistic representations of the system under study when combined [246]. Molecular dynamics trajectories can capture intricate details such as ion permeation events, binding/unbinding of the toxin, conformational changes of the receptor, and various protein-lipid and protein-solvent interactions at the atomic level [247–249]. The ever-increasing computing power available for research facilities establishes *in silico* approaches as central parts of venomic analysis pipelines. AI-driven structure predictions provide increasingly high-quality structural models and will gain importance for elucidation of toxin receptor interactions and accelerate the discovery of new promising venom-based therapeutic lead structures [250].

### Critical and future aspects of current bioassays

The door to powerful high-throughput assays available in other biological sciences has been opened by the refinement and miniaturization of test models in combination with recombinant and/or synthetic toxin production, as well as organoid venom-glands [121]. Increasingly, this allows broad screening of large numbers of toxins for action on a focussed biological target. Alternatively, a limited number of toxins may be screened on a large number of cell types or organisms, providing fast access to highly biological active toxins with the potential for novel and surprising applications. Indeed, *in vivo* large-scale toxin testing for phenotypic changes even on a whole-organism level such as flies, fishes, or nematodes can be performed in a high-throughput screening manner [221]. *In vitro* high-throughput electrophysiology in mammalian cells and *Xenopus* oocytes is also gaining importance [222]. *Ex vivo*, automatized “high-content screening” microscopy offers a versatile approach for testing many toxins, many cell types, or even both together. The automatization allows for simultaneous high-throughput acquisition of a large array of readouts such as various morphological features in combination with multiple immunocytochemical stainings on a single-cell basis [234,251]. Interestingly, classical pharmaceutical *in vitro* screening platforms with highly sensitive target-optimized cell assays such as fluorometric imaging plate reader (FLIPR), amplified luminescent proximity homogeneous assay (ALPHAscreen), and homogeneous time-resolved fluorescence (HTRF) screens, which are the workhorses for large-scale screening in the pharmacological industry, have so far only scarcely been applied to venom and its components [252,253]. It is anticipated that these methodologies, combined with microfluidic approaches, will propel the biological characterization of venoms and their toxins.

### Pharmaceutical applications

Venom compounds have a wide spectrum of pharmacological applications, including analgesic, anti-inflammatory, antimicrobial, and anti-cancer activities that have been used as prototypes for drug design and therapeutic agents and are used in a variety of therapeutical settings [2,218, 254–257]. Currently 11 toxin-based molecules have been approved by the US Food and Drug Administration (FDA) or the European Medicines Agency (EMA) and are on the market [2,15]. These venom-derived drugs are used for the treatment of hypertension, acute coronary syndromes, coagulation during surgery, chronic pain, type 2 diabetes, and perioperative bleeding, while many others are currently in clinical trials or in preclinical development. The original molecules were discovered predominantly in snakes (captopril, enalapril, tirofiban, eptifibatide, batroxobin, and cobratide), lizards (exenatide and lixisenatide), and several marine and terrestrial invertebrates from cone snails to leeches (e.g., ziconotide, bivalirudin, and desirudin) [15, 218,258–260]. However, critically it has to be noted that the whole process from bioprospecting to the final development of a compound for pharmaceutical applications remains challenging (Fig. 5C). In the following sections, we discuss challenges and highlight biological and ecological traits of venomous species that could greatly improve the effectiveness of this process.

***Targeting pain***

Severe pain is often one of the main symptoms of envenomation, especially in defensive venom, where toxins are instrumental in triggering aversive responses. This ability made venom toxins fundamental tools to investigate the physiology of nociception, which involves a number of receptors located in the peripheral nervous system, including the voltage-gated Na_v_, K_v_, and Ca_v_ channels and the ligand-gated transient receptor potential (TRP) channel, acid-sensing ion channel (ASIC), and P2X in the primary afferent neurons. AMPA (α-amino-3-hydroxy-5-methyl-4-isoxazolepropionic acid receptor), NMDA (glutamate-gated cation channels), NET (norepinephrine transporter), and GPCRs (G-protein-coupled receptors), together with Na_v_ and Ca_v_, affect modulation of pain at the spinal level [261]. Generally, agonists of these channels in nature elicit pain and trigger aversive responses [262], while antagonist toxins are extremely promising as analgesic drugs and indeed their efficacy as antinociceptives has been demonstrated by multiple studies in murine models. This is the case of toxins from the sea anemone *Heteractis crispa* that act as selective TRPV1 modulators and show analgesic effects in acute and chronic pain models in mice without causing hyperthermia, a common adverse effect of other TRPV1 antagonists [263]. On the contrary, crotalphine from the South American rattlesnake induces a potent and long-lasting analgesic effect in mice by activating and thus desensitizing the ankyrin-type TRPA1, which plays a critical role in the pathogenesis of pain and inflammation [264]. Several spider, snake, and sea anemone–derived toxins, including the well-characterized mambalgins, inhibit the activation of ASICs and are involved in different pain conditions [265–267]. A wide range of venom toxins target the voltage-gated Na_v_ channels, which are crucial in electrical signalling and neuromuscular function. Activators induce rigid paralysis and pain, while inhibitors are able to elicit spastic paralysis and analgesia, in both cases with a remarkable predatory and defensive effectiveness. Among them, the inhibitory cysteine knot (ICK) peptides, produced by spiders, scorpions, and cone snails, have been particularly studied [268,269]. Some ICK peptides also act as blocker of Ca_v_ channels including the cone snail o-conotoxin MVIIA (Prialt [ziconotide]), an FDA-approved analgesic for spinal administration in severe chronic pain [270]. Relatively few classes of toxins target GPCRs, including conotoxins, which are active against visceral and post-surgery pain through different mechanisms involving GABA_B_ and k-opioid receptors, NMDA, and NET [271,272]. Others are snake and spider toxins that modulate P2X and AMPA receptors to reduce inflammatory pain [273]. Overall, a variety of venom toxins have a great potential to be developed into novel analgesics that are able to block pain at its source [274].

***Anticancer applications***

Anticancer properties of animal toxins, which manipulate signalling cascades controlling cell death and tumour growth, are promising therapeutics [256,275–279]. In particular, peptides from spiders and octopus and the crude venom of various snake species (cobras and vipers) have recently been reported to specifically target human melanoma, often with minimal effects on healthy fibroblast cells [278–282]. Other anticancer activities of animal venoms highlight their potentials by inhibiting the proliferation and invasion of cancer cells, through cell cycle arrest and/or induction of apoptosis, as well as by revealing the affected signalling pathways [256, 275,283,284]. However, potent venoms with anticancer activities many times raise concerns regarding their toxicity in healthy, non-targeted cells and tissues [285]. These could be overcome by directly targeting tumour cells (e.g., nanoparticle-based delivery systems). In addition, combination approaches use venom or the active compound coupled with existing chemotherapeutic agents at a low dose [276,285,286]. However, toxic effects that emerge by the combination still need to be evaluated along with the observed anticancer or other therapeutic potential.

***Immunomodulation***

The potential immunomodulating abilities of venoms and toxins have also started to receive attention [281,287,288]. Immunosuppressive activity has been demonstrated in snake crude venoms. In particular, the activity of the red-bellied black snake *Pseudechis porphyriacus* venom might translate to therapeutic applications for T-cell–associated conditions including rheumatoid arthritis and inflammatory bowel disease [289]. Venom components from the rattlesnake *Crotalus durissus terrificus* specifically diminish T-cell proliferation and IL-2 production [290]. They induce a shift in the colonic microenvironment from proinflammatory to anti-inflammatory in mouse models of induced colitis [291]. These effects have been linked to the action of several specific toxins, belonging to different classes, including PLA2, cysteine-rich secretory proteins (CRISPs), metalloproteases, serine proteases, and L-amino acid oxidases (L-AAOs). In addition, many invertebrate venoms have been used by traditional medicine in different cultures to treat, among others, autoimmune diseases, from bees to scorpion [292,293]. Studies have confirmed that the 2 major bee venom components, melittin and apamin, regulate, respectively, T_H_2-cell–mediated responses [294] and the production of monocytes and macrophages [293]. On the other hand, both margatoxin from *Centruroides margaritatus* scorpions [295] and the *Stichodactyla* toxin (ShK) from the anemone *Stichodactyla helianthus* are able to selectively block the K_v_1.3 channel, a key component in autoimmune disease progression, highly expressed in effector memory T cells [296].

***Antimicrobial activity***

In the context of the current antibiotic crisis and the scarcity of therapeutic alternatives for the treatment of bacterial infections caused by multi-resistant bacteria or to treat viral infections, the search for new therapeutic alternatives is 1 persisting challenge. Antimicrobial peptides (AMPs) derived from animal venoms are biologically active cationic, anionic, or amphipathic peptides of <100 amino acid residues with a wide structural but stable range (α-helices, β-sheets, extended structures, or disordered loops) [297,298]. In this sense, AMPs and other metabolites obtained from animal venoms are a future solution to develop a new generation of synthetic antimicrobial molecules with improved antibacterial and antiviral activity, safety, and a broader spectrum of activity [297,299]. There are multiple examples of approved or promising AMPs described from various taxa such as serrulin or androctonin from scorpions, melittin and derivatives from bees, or L-AAO from snakes, to mention a few [300–303] (see Supplementary Tables S2–S4).

### Pore-forming toxins in sensing applications

**Figure 7.**
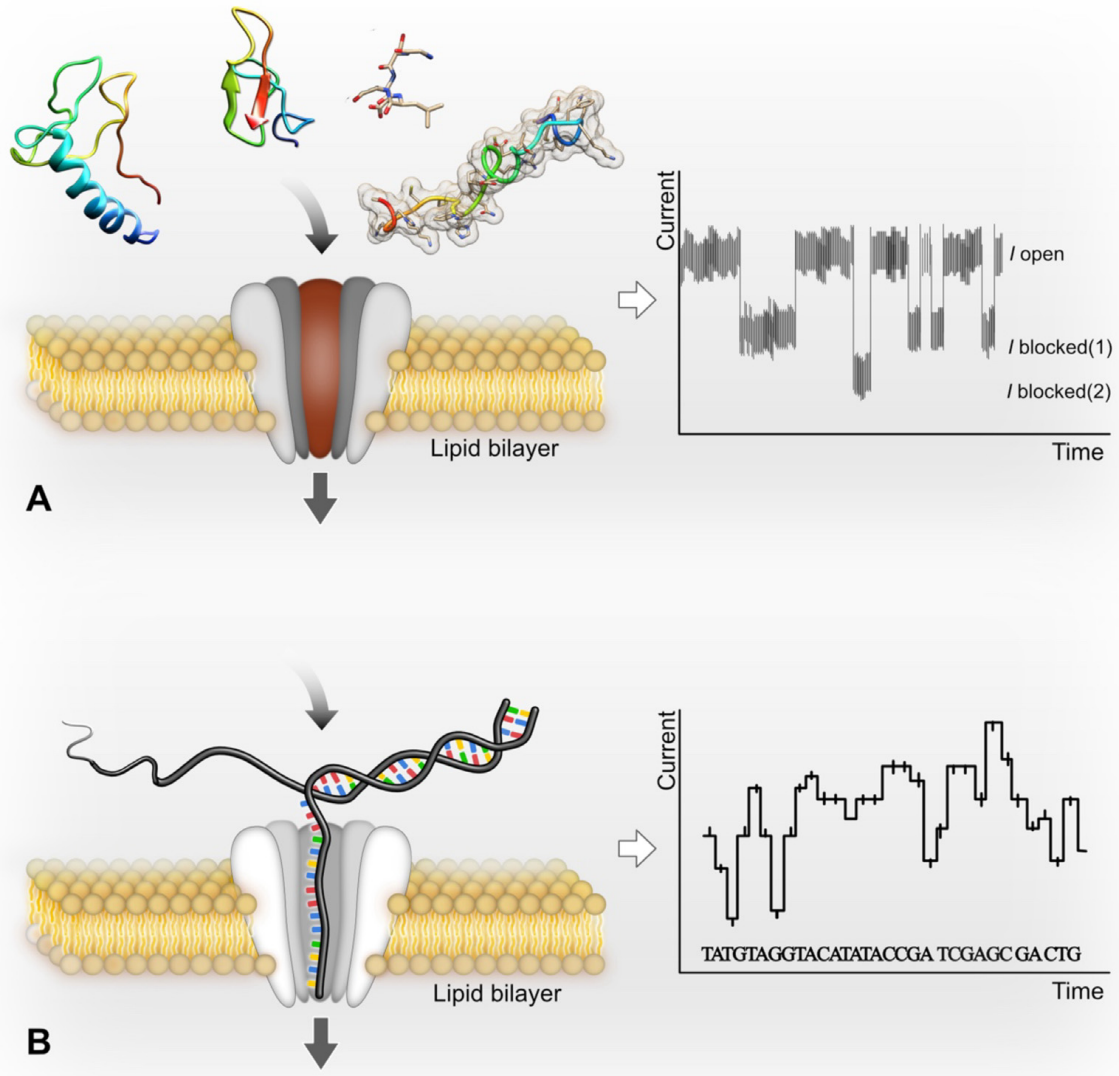
Schematic representation of nanopore biosensing. **(A)** Nanopore biosensing uses minute changes in electric current caused by the translocation of an analyte through the pore; each analyte is characterized by the percentage of current blockage and its duration. **(B)** The most widely used application of nanopore biosensing is DNA/RNA sequencing. The present trace is adapted from [312].

One of the most interesting recent applications of venom proteins is their use in nanopore biosensing, which allows detection of various small molecules, peptides, proteins, DNA and RNA, sequencing, and analysis of enzymatic reactions at the single-molecule level (Fig. 7A). Although prokaryotic channels or pore-forming proteins are most commonly used for nanopore biosensing [304], cytolytic venom proteins are also very attractive candidates owing to some advantageous properties [305]. These include channel stability, ease of insertion into artificial hydrophobic supports [306], and the ability to alter channel size through mutations that affect oligomerization [307]. Although the current nanopore sequencing set-up of Oxford Nanopore Technologies involves a prokaryotic transport channel [308], it is foreseeable that venom protein channels will be developed for use in MinION® devices, whether for long-read sequencing or other biosensing applications (Fig. 7B).

Using the classical patch-clamp method, channels formed by a toxin from the sea anemone *Actinia fragacea* have been used so far to detect DNA, peptides, proteins, and small molecules [309–311]. However, nanopore biosensing may require more or less extensive mutagenesis of protein residues to enable analyte capture or translocation. Prior structural and biochemical knowledge is therefore paramount for the adaptation of venom proteins for biosensing experiments. It is important to note that nanopore-based identification and sequencing is performed at the single-molecule level, which means that it can enable the discovery of new or rare (macro)molecules and create opportunities for the development of highly sensitive diagnostic devices.

### Agrochemical applications

The main approach to control pest species in agricultural and public health contexts relies on chemical pesticides. However, the continuous use of specific classes of insecticides has inevitably led to resistance in various pest species. In addition, current pesticides have a devastating impact on biodiversity [14,313–317] and have often raised concerns regarding human safety; owing to improved health legislation, many previously successful insecticides have been de-registered [318].

Altogether, these circumstances led to a renewed interest in the development of novel, eco-friendly bioinsecticides. Animal venoms, especially from predators that feed on insects, may be extremely promising for identifying novel natural insecticides, with strict species-specific action. The venom-derived insecticidal compounds tested to date have revealed a rich repertoire of bioactive compounds that specifically target ion channels of prey insects [14,319–321].

Novel spider peptides derived from the African and Australian Theraphosidae spiders, as well as the African *Augacepahuls ezendami*, have shown insecticidal capabilities for further development [322–324]. In line with this research approach, in 2017 the US-based company Vestaron launched the first peptide-based pesticide based on a knottin from a funnel web spider, thus validating the immense potential of animal venom-derivatives as bioinsecticides. The innovative aspect of this knottin is that it is sprayed on plants and then orally taken in by pest species. This makes conventional genetic modifications of plants by incorporating toxin genes in their genomes obsolete, and the creation of recently more critically seen gene modified organisms is avoided [14].

### Diagnostics

As a result of extensive toxin investigations mostly from the late 1980s, several *in vitro* diagnostic tests were developed, commercialized, and adopted as routine applications in hematology laboratories to be used for assessing hemostatic disorders. Many hemostatic parameters such as fibrinogen breakdown products, activation/inhibition of various clotting factors, protein C activation, von Willebrand factor–related disorders, and lupus anticoagulants can be assayed by using snake venom proteins, mostly proteinases [327, 328]. These tests have some advantages over other common assays with their unique mechanisms of action. For example, snake venom thrombin-like enzymes are generally not inhibited by thrombin inhibitors such as heparin, allowing the test to be performed with samples containing these inhibitors [325]. Detailed information about this topic can be obtained from cited references.

A more recent venom-based diagnostic tool was developed from a species of scorpion, *Leiurus quinquestriatus* (death stalker). One of its major venom components, chlorotoxin, which blocks chlorine channels, can also bind to matrix metalloprotease-2 (MMP-2), which is specifically upregulated on the membrane of cancer cells but not in normal cells. This unique feature has led to a diagnostic reagent, so-called “tumor paint,” which can be used for monitoring tumors. Chlorotoxin peptide is labelled with a fluorescent cyanine dye, which when subsequently bound to cancer cells selectively helps to visualize the borders of tumor tissue precisely. This is particularly useful in treating brain tumors because it is critical to be as precise as possible when excising tumors during surgery to prevent irreparable brain damage. Chlorotoxin is additionally being evaluated in clinical trials as an *in vivo* diagnostic imaging agent for various cancers, including glioma [218,329], and recently was granted a fast-track designation from the US FDA for pediatric brain tumors. We predict that with more detailed knowledge on toxins and their distribution and developmental fate within the venomous organisms further promising candidates with specific activities suitable for diagnostic applications will be identified.

### Envenomation therapy: antivenoms (in a nutshell)

Animal envenomation by several key taxa such as spiders, scorpions, and snakes is a major public health concern worldwide; however, most dramatic are effects from snake bites. Millions of individuals are at risk owing to their geographic location, which is inhabited by various lethal snakes, especially in Africa, the Middle East, India, Mexico, and South America [330]. Approximately 1.8 million people are annually bitten by snakes, of which 138,000 people die as a result of envenoming while up to 500,000 snake bite survivors experience permanent physical or psychological disabilities worldwide [19,28]. As a consequence, the World Health Organization included snakebite envenoming in the list of category A Neglected Tropical Diseases [331] and developed a strategy to reduce mortality and disability by 50% before 2030 [332].

Antivenom is the only specific and effective therapy for victims of envenomation. The active compounds reported are whole immunoglobulins G, their F(ab’)_2_ or Fab fragments extracted and purified from the hyperimmune plasma of large animals (mostly horse) and prepared by their immunization with a single venom or a mixture of several of them [333,334]. Unfortunately, we have a current, serious crisis in antivenom availability in such most endangered regions, like sub-Saharan Africa and tropical and subtropical Asia [335]. It is determined by cost, frequent scarcity, and poor distribution because only a few countries are the antivenom manufacturers (and only 3 in Europe). In addition, it may require a cold-chain for transport and storage, which is problematic for rural areas of low-to-middle income countries (LMIC). Additionally, some major antivenom manufacturers (Syntex, Behringwerke, and Sanofi Pasteur) have stopped antivenom production over the past two decades for commercial reasons, creating a noticeable deficit of antivenom in the countries that they previously supplied, especially in Africa [19]. Even Europe faces current antivenom shortages, due to the low financial sustainability of their production and lack of compliance to good manufacturing practice (GMP) regulations. Further, recent analyses revealed the lack of comparative information on available antivenoms against European vipers (see Supplementary Table S5 and cited references for more details).

Several promising new technologies have been presented in recent years for the manufacturing of therapeutic antibodies on an industrial scale as antivenoms. Some of these molecules include monoclonal antibodies, scfv (single-chain fraction variable fragments), and nanobodies among others that could form the basis for future treatments [334, 336]. However, to develop these new antivenomics platforms the most detailed knowledge of venom composition is crucial. The herein-discussed methods and future perspectives facilitate an unprecedented understanding of the ecology and biology of venomous animals and their venoms, which allows in consequence the production of more effective antivenoms.

## Conclusions

- Fast advancements in genomics, transcriptomics, and proteomics technologies increase our knowledge of convergently evolved venoms across the tree of life.
- A more detailed knowledge on toxins and their distribution and developmental fate within the venomous organisms will reveal new insights on their evolutionary origins while also identifying compounds with novel bioactivity and targets.
- Venom toxins possess a great translational potential, with applications in the therapeutic, diagnostic, agrochemical, and biosensing fields. More detailed biological insights on venomous species facilitate a more targeted identification of new promising candidates with specific activities suitable for known and novel applications.
- In particular, in the context of the current antibiotic crisis and the scarcity of therapeutic alternatives for the treatment of multidrug-resistant bacterial and viral infections, the search for new therapeutics is 1 persisting challenge that could be addressed by venom research.
- Owing to their devastating impact on biodiversity and concerns for human safety, there is great interest in replacing conventional pesticides with eco-friendly bioinsecticides. Animal venoms, especially from predators that feed on insects, may be extremely promising for identifying novel natural insecticides, with strict species-specific action.
- The whole process from bioprospecting to the final development of a compound for translational applications remains challenging. The approaches here outlined combining multiple aspects of animal venoms, including the biological and ecological traits of venomous species, would greatly improve the effectiveness of this process.

## Data Availability

Not applicable.

### Abbreviations

AMP: antimicrobial peptide; AMPA: α-amino-3-hydroxy-5-methyl-4-isoxazolepropionic acid receptor; ASIC: acid-sensing ion channel; BLAST: Basic Local Alignment Search Tool; cDNA: complementary DNA; CRISPR: clustered regularly interspaced short palindromic repeats; ESI: electrospray ionization; FDA: Food and Drug Administration; GPCR: G-protein-coupled receptors; GUI: graphical user interface; ICK: inhibitory cysteine knot; L-AAO: L-amino acid oxidase; LC-MS/MS: liquid chromatography–tandem mass spectrometry; mRNA: messenger RNA; MS: mass spectrometry; MSI: mass spectrometry imaging; NET: norepinephrine transporter; NMDA: glutamate-gated cation channels; NMR: nuclear magnetic resonance; PLA_2_: phospholipase A_2_; PTM: posttranslational modification; scRNA-seq: single-cell RNA-sequencing; TRP: transient receptor potential.

## Competing Interests

The authors declare that they have no competing interests.

## Funding

This work is funded by the European Cooperation in Science and Technology (COST, www.cost.eu) and based upon work from the COST Action CA19144 – European Venom Network (EUVEN, see https://euven-network.eu/). This review is an outcome of EUVEN Working Group 2 (“Best practices and innovative tools in venomics”) led by B.M.v.R. As coordinator of the group Animal Venomics until end 2021 at the Institute for Insectbiotechnology, JLU Giessen, B.M.v.R. acknowledges the Centre for Translational Biodiversity Genomics (LOEWE-TBG) in the programme “LOEWE – Landes-Offensive zur Entwicklung Wissenschaftlich-ökonomischer Exzellenz” of Hesse's Ministry of Higher Education, Research, and the Arts. B.M.v.R. and I.K. further acknowledge funding on venom research by the German Science Foundation to B.M.v.R. (DFG RE3454/6-1). A.C., A.V., and G.Z. were supported by the European Union's Horizon 2020 Research and Innovation program through Marie Sklodowska-Curie Individual Fellowships (grant agreements No. A.C.: 896849, A.V.: 841576, and G.Z.: 845674). M.P.I. is supported by the TALENTO Program by the Regional Madrid Government (2018-T1/BIO-11262). T.H.'s venom research is funded by the DFG projects 271522021 and 413120531. L.E. was supported by grant No. 7017-00288 from the Danish Council for Independent Research (Technology and Production Sciences). N.I. acknowledges funding on venom research by the Research Fund of Nevsehir Haci Bektas Veli University (project Nos. ABAP20F28, BAP18F26). M.I.K. and A.P. acknowledge support from GSRT National Research Infrastructure structural funding project INSPIRED (MIS 5002550). G.A. acknowledges support from the Slovenian Research Agency grants P1-0391, J4-8225, and J4-2547. G.G. acknowledges support from the Institute for Medical Research and Occupational Health, Zagreb, Croatia. E.A.B.U. is supported by a Norwegian Research Council FRIPRO-YRT Fellowship No. 287462.

## Authors' Contributions

Lead, major conceptualization, first draft, graphics, and illustration by B.M.v.R.; all authors wrote the main text and edited the final manuscript. Except for the first author, authors are listed alphabetically with respect to the last name. All authors have read and agreed to the published version of the manuscript.

## Supplementary Material

giac048_GIGA-D-22-00023_Original_Submission

giac048_GIGA-D-22-00023_Revision_1

giac048_Response_to_Reviewer_Comments_Original-Submission

giac048_Reviewer_1_Report_Original_Submission Mark J. Margres -- 2/17/2022 Reviewed

giac048_Reviewer_2_Report_Original_Submission Matthew Holding -- 2/21/2022 Reviewed

giac048_Reviewer_3_Report_Original_Submission Jason Macrander, Ph. D. -- 3/8/2022 Reviewed

giac048_Supplemental_File
