# Supplementary material for: Modern venomics—Current insights, novel methods, and future perspectives in biological and applied animal venom research
Source: Gigascience. 2022 May 18;11:giac048. doi: 10.1093/gigascience/giac048 (PMC9155608; doi:10.1093/gigascience/giac048)
Supplement: giac048_GIGA-D-22-00023_Original_Submission [file giac048_giga-d-22-00023_original_submission.pdf]

GigaScience

Modern venomics – Current insights, novel methods and future perspectives in biological and applied animal venom research

--Manuscript Draft--

**Manuscript Number:**	GIGA-D-22-00023
**Full Title:**	Modern venomics – Current insights, novel methods and future perspectives in biological and applied animal venom research
**Article Type:**	Review
**Funding Information:**	European Cooperation in Science and Technology (CA19144) — Dr Maria Vittoria Modica
**Abstract:**	Venoms have evolved over 100 times in all major animal groups and their components, known as toxins, have been fine-tuned over millions of years into highly effective biochemical weapons. Except for a few better studied taxasuch as snakes, scorpions or spiders, many evolutionary questions are still disputed regarding this toxin arsenal, for example how venom genes originate, how venom contributes to the fitness of venomous species, and which modifications at the genomic, transcriptomic and protein level drive their evolution. More recently, venom compounds have become a source of inspiration for various translational research aspects. We highlight here recent advances and new strategies in modern venomics by combining the evolutionary and the applied perspective on this topic. We discuss how recent technological innovations and multi-omic methods dramatically improved research on venomous animals. The study of genomes and their modifications through CRISPR and knockdown technologies will increase our understanding of how toxins evolve and which functions they have in the different ontogenetic stages during the development of venomous animals. Mass spectrometry imaging combined with spatial transcriptomics, in situ hybridisation techniques, and modern computer tomography gives us further insights into the spatial distribution of toxins in the venom system and the function of the venom apparatus. All these evolutionary and biological insights contribute to identify more efficiently venom compounds, which can be then synthesized or produced in adapted systems to test their bioactivity. Finally, we critically discuss recent agrochemical, pharmaceutical, therapeutic, and diagnostic aspects of venoms.
**Corresponding Author:**	Bjoern Marcus von Reumont<br>University of Gießen<br>GERMANY
**Corresponding Author Secondary Information:**	
**Corresponding Author's Institution:**	University of Gießen
**Corresponding Author's Secondary Institution:**	
**First Author:**	Bjoern Marcus von Reumont
**First Author Secondary Information:**	
**Order of Authors:**	Bjoern Marcus von Reumont
	Gregor Anderluh
	Agostinho Antunes
	Naira Ayvazyan
	Dimitris Beis
	Figen Caliskan
	Ana Crnkovic
	Maik Damm

Powered by Editorial Manager® and ProduXion Manager® from Aries Systems Corporation

	Sebastien Dutertre
	Lars Ellgard
	Goran Gajski
	Hannah German
	Beata Halassy
	Benjamin-Flolrian Hempel
	Tim Hucho
	Nasit Igci
	Maria P Ikonomopoulou
	Izhar Kabat
	Maria I Klapa
	Ivan Koludarov
	Jeroen Kool
	Tim Lueddecke
	Rhiad Ben Mansour
	Maria Vittoria Modica
	Yehu Moran
	Ayse Nalbantsoy
	Maria Eugenia Pachon-Ibanez
	Alexios Panagiotopoulos
	Eitan Reuveny
	Javier Sanchez Cespedes
	Andy Sombke
	Joachim M SUrm
	Eivind ANdreas Baste Undheim
	Aida Verdes
	Giulia Zancolli
**Order of Authors Secondary Information:**	
**Additional Information:**	
**Question**	**Response**
Are you submitting this manuscript to a special series or article collection?	No; No
**Experimental design and statistics**<br><br>Full details of the experimental design and statistical methods used should be given in the Methods section, as detailed in our Minimum Standards Reporting Checklist. Information essential to interpreting the data presented should be made available in the figure legends.	Yes; Yes

Powered by Editorial Manager® and ProduXion Manager® from Aries Systems Corporation

Have you included all the information requested in your manuscript?	
**Resources**<br><br>A description of all resources used, including antibodies, cell lines, animals and software tools, with enough information to allow them to be uniquely identified, should be included in the Methods section. Authors are strongly encouraged to cite Research Resource Identifiers (RRIDs) for antibodies, model organisms and tools, where possible.<br><br>Have you included the information requested as detailed in our Minimum Standards Reporting Checklist?	Yes; Yes
**Availability of data and materials**<br><br>All datasets and code on which the conclusions of the paper rely must be either included in your submission or deposited in publicly available repositories (where available and ethically appropriate), referencing such data using a unique identifier in the references and in the "Availability of Data and Materials" section of your manuscript.<br><br>Have you have met the above requirement as detailed in our Minimum Standards Reporting Checklist?	Yes; Yes

Powered by Editorial Manager® and ProduXion Manager® from Aries Systems Corporation

Manuscript Click here to access/download;Manuscript;vonReumont_etal_ModernVeno

Review

Modern venomics – Current insights, novel methods and future perspectives in biological and applied animal venom research

Bjoern M von Reumont [1,2], Gregor Anderluh [3], Agostinho Antunes [4,5], Naira Ayvazyan [6], Dimitris Beis[7], Figen Caliskan[8], Ana Crnković[3], Maik Damm[9], Sebastien Dutertre[10], Lars Ellgaard[11], Goran Gajski[12], Hannah German[13], Beata Halassy[14], Benjamin-Florian Hempel[15], Tim Hucho[16], Nasit Igci[17], Maria P. Ikonomopoulou[18, 19], Izhar Karbat[20], Maria I. Klapa[21], Ivan Koludarov[1], Jeroen Kool[13], Tim Lüddecke[2,22], Riadh Ben Mansour[23], Maria Vittoria Modica[24], Yehu Moran[25], Ayse Nalbantsoy[26], María Eugenia Pachón Ibáñez[27,28], Alexios Panagiotopoulos[21,29], Eitan Reuveny[20], Javier Sánchez Céspedes[27,28], Andy Sombke[30], Joachim M. Surm[25], Eivind Undheim[31, 32], Aida Verdes[33], Giulia Zancolli[34,35]

[1] Justus Liebig University Giessen, Institute for Insectbiotechnology, Heinrich Buff Ring 26-32, 35396 Giessen, Germany, bmvr@reumont.net

[2] LOEWE Centre for Translational Biodiversity Genomics, Senckenberg Frankfurt, Senckenberganlage 25, 60235 Frankfurt, Germany

[3] Department of Molecular Biology and Nanobiotechnology, National Institute of Chemistry, 1000 Ljubljana, Slovenia, gregor.anderluh@ki.si; ana.crnkovic@ki.si

[4] CIIMAR/CIMAR, Interdisciplinary Centre of Marine and Environmental Research, University of Porto, Terminal de Cruzeiros do Porto de Leixões, Av. General Norton de Matos, s/n, 4450–208 Porto, Portugal, aantunes@ciimar.up.pt

[5] Department of Biology, Faculty of Sciences, University of Porto, Rua do Campo Alegre, 4169-007, Porto, Portugal.

[6] Orbeli Institute of Physiology of NAS RA, Orbeli ave. 22, 0028, Yerevan, Armenia

[7] Developmental Biology, Centre for Clinical, Experimental Surgery and Translational Research, Biomedical Research Foundation Academy of Athens, Athens 11527, Greece. dbeis@bioacademy.gr

[8] Department of Biology, Faculty of Science and Letters, Eskisehir Osmangazi University, TR-26040 Eskisehir, Turkey. fcalis@ogu.edu.tr

[9] Technische Universität Berlin, Department of Chemistry, Straße des 17. Juni 135, 10623 Berlin, Germany, maik.damm@tu-berlin.de

[10] IBMM, Univ Montpellier, CNRS, ENSCM, 34095 Montpellier, France sebastien.dutertre@umontpellier.fr

[11] Department of Biology, University of Copenhagen, DK-2200 Copenhagen, Denmark, lellgaard@bio.ku.dk

[12] Institute for Medical Research and Occupational Health, Mutagenesis Unit, Ksaverska cesta 2, 10000 Zagreb, Croatia, ggajski@imi.hr

[13] Amsterdam Institute of Molecular and Life Sciences, Division of BioAnalytical Chemistry, Faculty of Science, Vrije Universiteit Amsterdam, De Boelelaan 1085, 1081HV Amsterdam, The Netherlands, j.kool@vu.nl

[14] University of Zagreb, Centre for Research and Knowledge Transfer in Biotechnology, Trg Republike Hrvatske 14, 10000, Zagreb, Croatia, bhalassy@unizg.hr

[15] BIH Center for Regenerative Therapies BCRT, Charité - Universitätsmedizin Berlin, Augustenburger Platz 1, 13353 Berlin, Germany, benjamin.hempel@charite.de

[16] Translational Pain Research, Department of Anesthesiology and Intensive Care Medicine, Faculty of Medicine and University Hospital Cologne, University of Cologne, 50931 Cologne, Germany, tim.hucho@uk-koeln.de

[17] Nevsehir Haci Bektas Veli University, Faculty of Arts and Sciences, Department of Molecular Biology and Genetics, 50300, Nevsehir, Turkey, igcinasit@yahoo.com.tr

[18] Madrid Institute for Advanced Studies in Food, Madrid, E28049, Spain, maria.ikonomopoulou@imdea.org

[19] The University of Queensland, St Lucia, QLD 4072, Australia

[20] Department of Biomolecular Sciences, Weizmann Institute of Science, Rehovot 76100, Israel, izhar.karbat@weizmann.ac.il

[21] Metabolic Engineering and Systems Biology Laboratory, Institute of Chemical Engineering Sciences, Foundation for Research & Technology Hellas (FORTH/ICE-HT), Patras GR-26504, Greece, mklapa@iceht.forth.gr, alexispan556677@gmail.com

[22] Department of Bioresources, Fraunhofer Institute for Molecular Biology and Applied Ecology, 35392, Gießen, Germany, tim.lueddecke@outlook.com

[23] Department of Life Sciences, Faculty of Sciences, Gafsa University, Campus Universitaire Siidi Ahmed Zarrouk, 2112 Gafsa, Tunisia, riadh.benmansour@fsgf.rnu.tn

[24] Dept. of Biology and Evolution of Marine Organisms (BEOM), Stazione Zoologica Anton Dohrn, Via Po 25c, I-00198 - Roma, Italy, mariavittoria.modica@szn.it

[25] Department of Ecology, Evolution and Behavior, Alexander Silberman Institute of Life Sciences, Faculty of Science, The Hebrew University of Jerusalem, Jerusalem 9190401, Israel. yehu.moran@mail.huji.ac.il; joachim.surm@mail.huji.ac.il

[26] Department of Bioengineering, Faculty of Engineering, Ege University, 35100 Bornova, Izmir, Turkey, analbantsoy@gmail.com, ayse.nalbantsoy@ege.edu.tr

[27] Unit of Infectious Diseases, Microbiology, and Preventive Medicine, Virgen del Rocío University Hospital, Institute of Biomedicine of Seville, Seville, Spain, jsanchez-ibis@us.es, mpachon-ibi@us.es

[28] CIBER de Enfermedades Infecciosas, Instituto de Salud Carlos III, Madrid, Spain

[29] Animal Biology Division, Department of Biology, University of Patras, Patras, GR-26500, Greece

[30] Department of Evolutionary Biology, University of Vienna, Djerassiplatz 1, 1030 Vienna, Austria, andy.sombke@gmx,de

[31] Centre for Biodiversity Dynamics, Department of Biology, Norwegian University of Science and Technology, 7491 Trondheim, Norway

[32] University of Oslo, Centre for Ecological and Evolutionary Synthesis, Postboks 1066 Blindern 0316 Oslo, Norway, e.a.b.undheim@ibv.uio.no

[33] Department of Biodiversity and Evolutionary Biology, Museo Nacional de Ciencias Naturales, José Gutiérrez Abascal 2, 28006, Madrid, Spain, aida.verdes@mncn.csic.es

[34] Department of Ecology and Evolution, University of Lausanne, 1015 Lausanne, Switzerland, giulia.zancolli@gmail.com

[35] Swiss Institute of Bioinformatics, 1015 Lausanne, Switzerland

Corresponding author: BMvR, bmvr@reumont.net

Abstract

Venoms have evolved over 100 times in all major animal groups and their components, known as toxins, have been fine-tuned over millions of years into highly effective biochemical weapons. Except for a few better studied taxasuch as snakes, scorpions or spiders, many evolutionary questions are still disputed regarding this toxin arsenal, for example how venom genes originate, how venom contributes to the fitness of venomous species, and which modifications at the genomic, transcriptomic and protein level drive their evolution. More recently, venom compounds have become a source of inspiration for various translational research aspects. We highlight here recent advances and new strategies in modern venomics by combining the evolutionary and the applied perspective on this topic. We discuss how recent technological innovations and multi-omic methods dramatically improved research on venomous animals. The study of genomes and their modifications through CRISPR and knockdown technologies will increase our understanding of how toxins evolve and which functions they have in the different ontogenetic stages during the development of venomous animals. Mass spectrometry imaging combined with spatial transcriptomics, *in situ* hybridisation techniques, and modern computer tomography gives us further insights into the spatial distribution of toxins in the venom system and the function of the venom apparatus. All these evolutionary and biological insights contribute to identify more efficiently venom compounds, which can be then synthesized or produced in adapted

systems to test their bioactivity. Finally, we critically discuss recent agrochemical, pharmaceutical, therapeutic, and diagnostic aspects of venoms.

Keywords

Venom, Modern Venomics, Genomics, Spatial -omics, Evolution, Translational research, Bioassays, Envenomation, Antivenom, Toxin production

1. Background - Why venoms matter

Venomous animals fascinate and affect humankind from time immemorial and influence - often unnoticed - many cultural, ecological, and economical aspects of our life [1,2]. Venom is such an effective adaptation ensuring the fitness of many species, that it has evolved independently over 100 times, across all major animal lineages, where it is predominately used for defense or predation [3–5]. Venomous species play key roles in interaction networks in almost all natural habitats, ensuring the stability of food-webs. We are just starting to understand many of these relationships, through recent advances in the knowledge of the biology of many venomous animals, the ecological implications of such a complex trait as venom, and its dynamic composition [5–7].

Venom is predominantly used in interspecific interaction, including both predation (such as in spiders, scorpions, centipedes, snakes) and defence (typical examples include bees, sea urchins and fishes) [5]. In each lineage, coevolution has refined venom components—often presumed to be through an arms race process—make them highly effective disruptors of physiological processes. The remarkable target specificity of many venom compounds stimulated early interest in their potential uses for applied and translational research. As a

result, molecules from a few selected taxa such as cone snails, snakes, spiders, and scorpions have been characterized in complex studies aiming at exploring their bioactivity over the course of decades [1,2]. Today, toxins are used in a variety of translational sectors including therapeutics, sustainable bioinsecticides in agrochemistry and clinical markers in diagnostics [1,2,8–10], see Figure 1.

The integrative research area in which all these aspects of animal venoms are studied is nowadays coined modern venomics [11]. However, venoms come not only as a cure, they also cast shadows. Clinical effects of envenomations are frequently untreated since effective and cheap antivenoms, even for the most notorious snakes, spiders, scorpions and bees, are often lacking. This is one of the urgent humanitarian challenge, especially in countries where envenomations are frequent [12–17]. Moreover, many venomous neozoic species that invade new ecosystems facilitated by climate change pose not only threats to humans but also to native species and livestock [18,19].

Here we summarize current challenges and approaches on the most relevant theoretical, basic, and applied research disciplines of modern venomics (see Figure 1). In addition, we highlight future directions and most promising innovations in methods, technology and platforms that can contribute to animal venom research. The structure of this review reflects the typical workflow of venom studies, from the collection of venomous organisms all the way towards the applied research, with the aim to be easily used as a blueprint for future venomics studies and a roadmap towards new methodological perspectives.

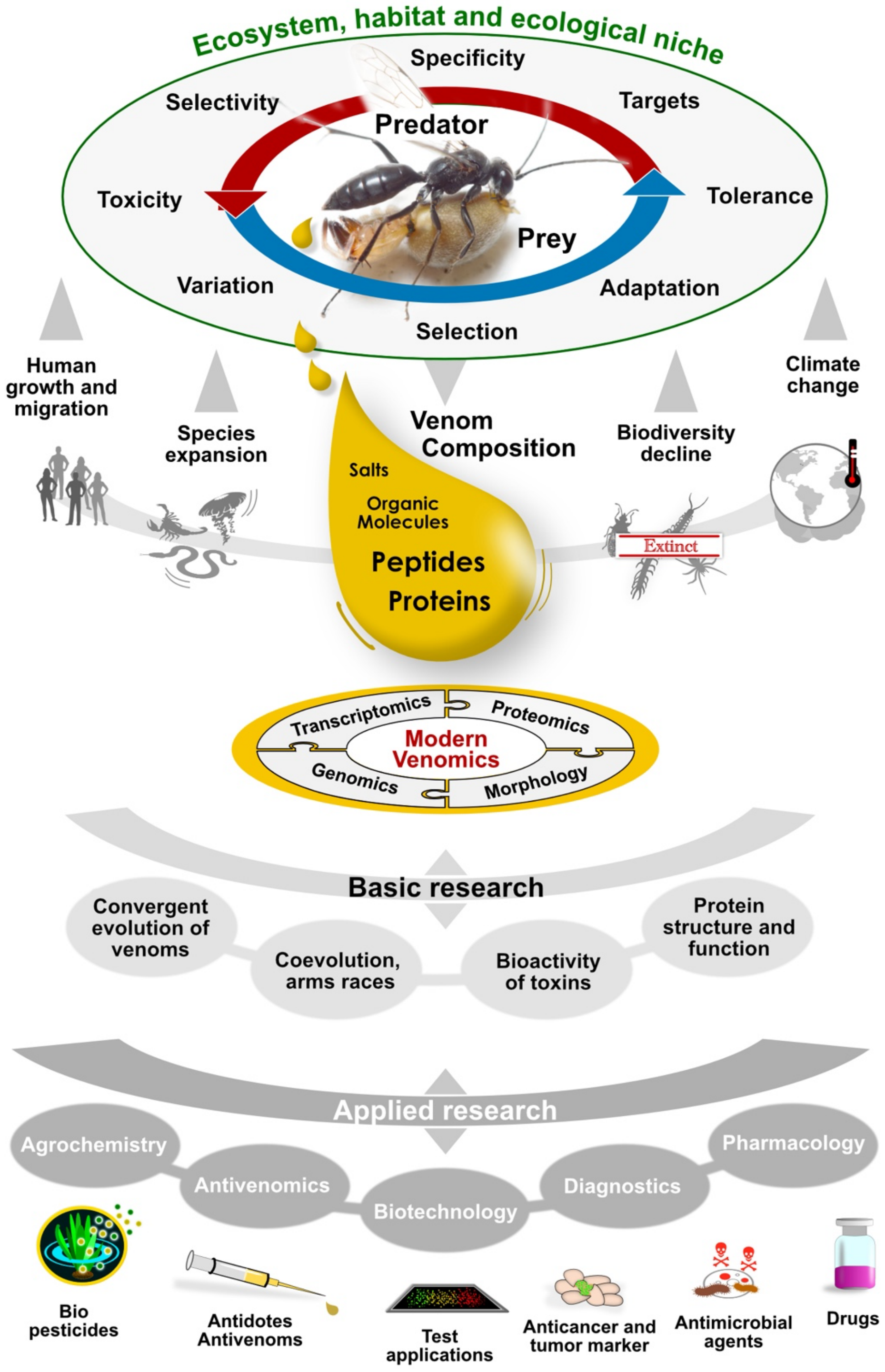

Figure 1. The importance and impact of venom as an evolutionary trait. The biology and ecology of venomous species prompt diversity of venoms, which are constituted of highly specific toxin components that were adaptively produced over time. Predator-prey interactions are major evolutionary forces that often trigger arms races of venom toxicity and resistance. Extrinsic factors that affect venomous species and their interaction with humans include species expansion or decline (linked to the biodiversity crisis and climate change) but also the increasing human growth and migration. The basic venom research investigates why and how venoms and toxin genes evolve based on modern 'omics' methods. Translational research exploits these basic studies for developing various applications, ranging from pharmacology (e.g. anti-pain and anti-cancer drugs, diagnostic markers, antivenom development) to agrochemistry (pesticides, antiparasitic compounds for crop and livestock protection) and biotechnology (e.g. nanopore sensing).

2. Collection of venomous organisms

2.1 Taxonomic expertise on venomous animals

Most studies on venomous animals start by sampling, identifying and collecting specimens of venomous species. Until recently, studies almost exclusively focused on taxa that were harmful to humans, such as snakes, spiders, and scorpions, driven partially by the need to mitigate the effects of envenomations [1,5,6]. The increasing collection of so far understudied species, particularly invertebrates, raises particular attention to a general, persisting impediment that affects all branches of modern zoology. In fact, for many animal groups taxonomic expertise has been declining for decades, precluding the precise assessment of global biodiversity and its trends [20–22]. New strategies to maintain and nurture taxonomic expertise in a biodiversity-driven biodiscovery approach have relevant impact on the field of venomics, especially since venom composition can vary between even closely related species [23].

2.2 Legal collection aspects

A long overdue awareness of equally shared bioresources and responsible collection of species emphasizes old and new legal aspects linked to field work. Naturally, researchers obtain official collection permissions for fieldwork linked to the collection locality and conservation status of the target species. More demanding, however, are the rather novel rules established by the international agreement on 'Access and Benefit Sharing, ABS' of the Convention of Biological Diversity. This agreement aims to standardize a legal framework for the access, transfer, utilization and benefits of genetic resources in a fair and equitable way for the providing country [24]. The resulting Nagoya protocol is currently enforced in

131 countries worldwide [25] leading on one hand to obvious benefits, such as a legal framework that prevents biopiracy and protects biodiversity, scientists and traditional medicines in the countries of origin, but also to technocratic hurdles which often hinder collaborative research and in particular translational applications [26–28]. These issues should be more explicitly addressed in the framework of the critical debate around the Nagoya protocol and its implementation.

2.3 Various venom systems require different methods to obtain crude venom

The tremendously diverse venom systems in most animals and the complex anatomy of their venom apparatus [5] require different approaches to collect crude venom. A well-known venom collection method is the milking of front-fanged snakes, where the animals are forced to bite through a thin membrane and release their venom into a clean glass vessel. In contrast, rear-fanged snakes are usually injected with pilocarpine to increase salivation and the released venom is collected manually from the fangs [29]. Similar pilocarpine-based methods have been established for venomous lizards, mammals and amphibians [29–31].

Fish venoms are often extracted from living or frozen specimens by dissecting their venom glands. Many fishes do not have distinct venom glands but clustered, venom producing, secretory cells that end in a spine groove [32]. For those species, protocols were developed in which crude venoms are extracted through a syringe or by a forced sting into a sponge contained in a tube [33,34]. Chemical extraction from partial- and whole-body samples represents the predominant way of venom collection in many marine invertebrates including echinoderms and several cnidarians [35–38]. For cnidarians, however, alternative protocols that are based on chemically induced discharge were likewise designed [39]. Cone snail venom can be collected by using live prey as lure or a predator as threat, which stimulates the

cones to shoot their venom harpoon into microcentrifuge tubes [40,41]. Venoms of most arthropods such as centipedes, chelicerates, crustaceans and insects are obtained by electrical, mechanical or chemical stimulation of venom ejection or dissection of the venom system, [42–47]. All these protocols have their pros and cons in terms of convenience and venom yield, but whenever possible, the most "natural" collection method should be preferred. For instance, electrostimulation is known to reveal differing venom profiles compared to manually collected venoms, calling for a cautionary interpretation of putative ecological roles of venoms without the support of further evidence, see e.g. [48,49]. A comprehensive overview of major venom collection protocols is given in Supplementary Table 1. After collection, obtained venom samples are usually pre-purified, then lyophilized and stored in freezers [29,50]. The compositions of these crude venom samples can be subsequently analysed using proteomic, metabolomic and transcriptomic methods, see Figure 2.

3. Venom metabolomics

3.1 Metabolitic molecules are often neglected

Metabolic profiling of venom refers to targeted and untargeted analysis of its composition of small molecular weight compounds. It is expected that at least some of these metabolites can act as regulatory molecules or direct metabolic intermediates of biological processes in the target species of venom-producing organisms [51–53]. However, holistic quantitative analyses of the venom metabolic composition in various species, the parameters affecting it, how the metabolite profile is related to the protein content, and commonalities and differences in the venom metabolic profile between species have not been carried out yet. In this sense, metabolic profiling of venoms is still a rather young research field [51] but gradually picking up. Current studies have indicated a surprising richness of the venom metabolic profile across species,

which need to be further explored with respect to both its biological role and potential biotechnological impact, e.g. [51].

In the case of snake venom, the information about small molecule composition remains largely qualitative rather than quantitative. While targeted studies of small molecules in snake venoms date back to 1936, it is only in the last 15 years that the presence of tens to hundreds of small molecules was reported in these venoms [54,55]. Recent studies reported ~ 200 metabolites [56] or ~50 lipids [57] in snake venoms. Small molecules can be main venom components, as in the case of acylpolyamines, a group of small neurotoxins with a molecular weight less than 1 kDa that inhibit glutamatergic synapses, structurally characterized in several spider genera using nuclear magnetic resonance (NMR) spectroscopy and liquid chromatography-tandem mass spectrometry (LC-MS/MS) approaches [58,59], and subsequently identified also in snake venom [55]. It has been postulated that polyamines induce hypotension and direct paralysis, facilitating prey hunting. Following the optimized workflow of these initial studies, Schroeder *et al.* used an untargeted NMR- and LC-MS/MS-based metabolomics approach for widespread identification of thus far undescribed small molecules in venoms of over 70 different spider species [60]. They identified small-molecular polyamines, neurotransmitters, nucleosides, amino acid derivatives and organic acids. Known and novel low molecular mass compounds from spiders are provided in VenMS, a newly available database [61].

Metabolomics studies have also been carried out on insect venoms. The venom of several species of fire ants (genus *Solenopsis*) contains a characteristic group of piperidine alkaloids [62], in both cis and trans stereoisomers, the trans isomer being dominant as retrieved in gas chromatography-mass spectrometry (GC-MS) [63,64]. More recent studies have been conducted on honeybees [65,66] and wasps [67] where untargeted and targeted LC-MS(/MS) analyses identified and quantified several organic acids, amines, amino acids and carbohydrates.

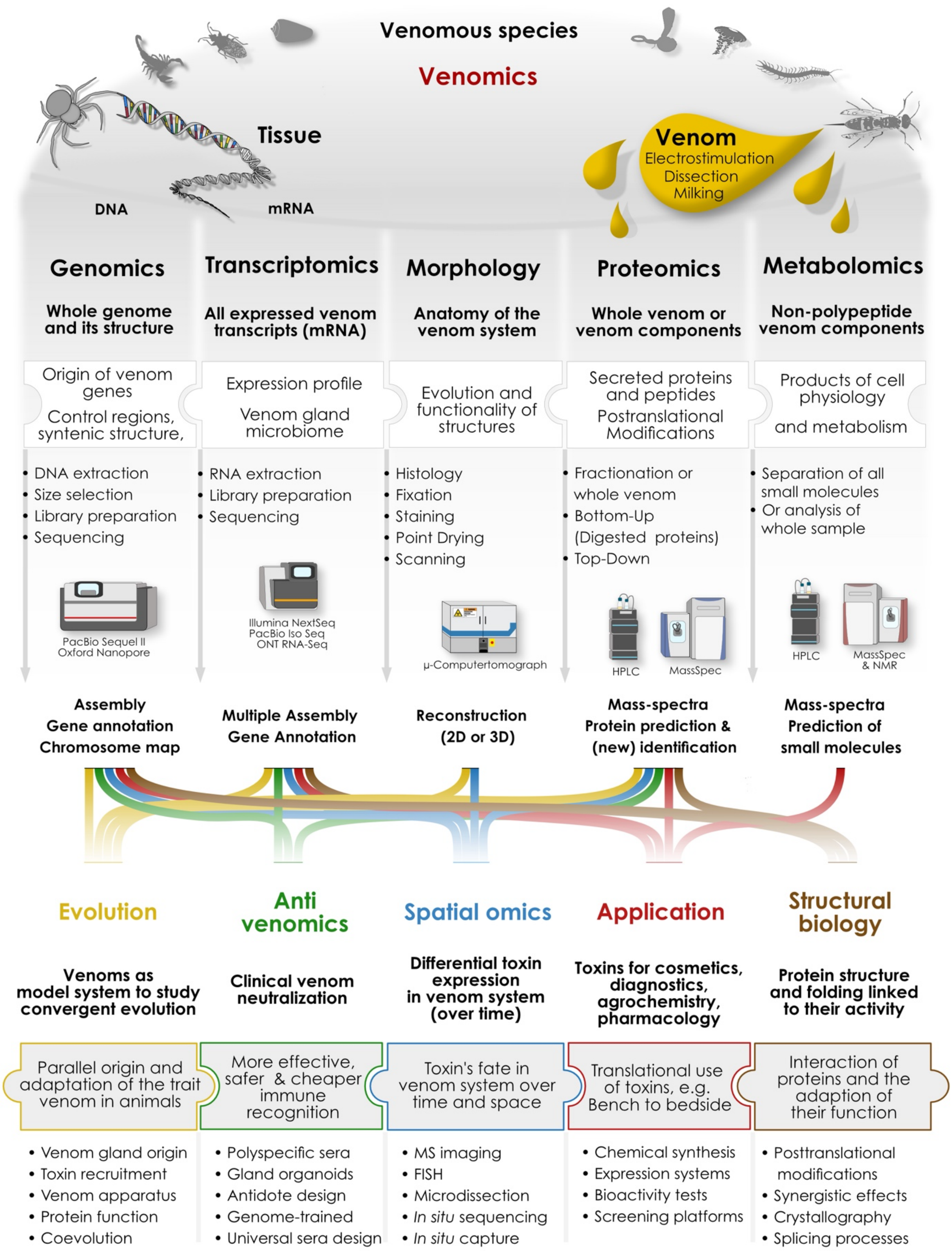

Figure 2: The major interdisciplinary research areas in venomics. The basic, interlinked, modern research fields in venomics are shown in the first row, and linked through simplified workflows with the final output(s). The main applied and evolutionary questions addressed are shown in the bottom, and integrated in the relevant topics. The flow diagrams that connect most research areas with each other illustrate the highly integrative nature of modern venomics.

4. Proteome analyses of crude venoms

Since animal venoms are predominantly of proteinaceous nature, nowadays state-of-the-art mass spectrometry (MS) instruments are used to describe venom proteomes, even in small organisms which deliver minute amounts of venom [49,68]. MS-based venom proteomics are used for i) general characterization of venom proteomes at the protein family level, ii) partial or full sequencing of (purified) venom peptides and proteins, iii) accurate mass determination of peptides and proteins either in crude venom (mass fingerprinting) or after purification, iv) relative or absolute quantitation of venom proteins and peptides, v) effective antivenom production (antivenomics), and vi) 3D structure elucidation by hydrogen deuterium exchange-MS and/or cross-linking MS methods [23,49,69–73].

4.1 Advantages and challenges of bottom-up and top-down approaches

In general, the methodological roadmap for any proteomic analysis in venom research is split into two major approaches: bottom-up and top-down proteomics [74–76] (Figure 2). In a bottom-up experiment, intact polypeptides are cleaved by proteases (generally trypsin) and the resulting peptide fragments are analysed by tandem MS. Top-down approaches in contrast describe the native form of venom proteins without any prior degradation. Thereafter, internal fragmentation processes by built-in collision cells of the MS instrument allow for toxin identification, which are well covered in other reviews and therefore not further elaborated here [71,76].

Bottom-up proteomics, achieved by in-solution digestion and direct MS analysis without prior decomplexation (shotgun proteomics), allows for a fast qualitative overview, but suffers from the critical ‘protein inference problem’ that often hinders the differentiation of the numerous toxin isoforms [77]. Therefore, a decisive factor for an extensive quantitative

venom analysis involves usually an upstream decomplexation and/or purification (clean-up) of the crude venoms applying several complementary separation methods, either by liquid chromatography (LC), gel electrophoresis, or a combination of both [74]. The existing decomplexation protocols can be adapted to many different instrumental setups and provide a detailed quantitative overview to characterize manifold toxin families. Nevertheless, sample preparation is less suitable for high-throughput analyses since it requires large quantities of venom samples and is more prone to contamination that results in false-positive identification of venom peptides [75]. Furthermore, trypsin digestion often prevents the clear identification of different toxin variants, like isoforms, proteoforms or complex multimer formations [78,79]. To bypass these limitations, a logical step is to eliminate the digestion step and directly analyze intact toxin proteins by tandem mass spectrometry, in a top-down proteomic approach [80].

In top-down methods crude venom samples are directly loaded to a front-end LC system coupled to the MS instrument. This setup enables for intact toxin mass profiling (MS1) and resolves toxin proteoforms and native posttranslational modifications (PTMs), that are not detectable by bottom-up approaches [80]. In order to identify the toxin proteins, information by tandem MS (MS2) in data-dependent acquisition (DDA) mode is acquired. Therefore, a specific peptide ion is delivered for fragmentation to obtain its MS /MS spectrum. The established workflow reduced the needed venom amount as well as operational time, and it is associated to a much lower contamination risk [81]. However, top-down venom proteomics requires a highly specific setup of high-resolution MS instruments that are only available in specialized laboratories [82]. In the case of high molecular mass toxin proteins, top-down analysis remains challenging and only provides few observable fragments in tandem MS due to inefficient ionization by denaturing electrospray ionization (ESI) [83].

4.2 Shortcomings in bottom-up and top-down approaches

Until today, most of the venom proteome studies use one of the well-established bottom-up strategies [49]. A shortcoming of this approach is the bias in protein quantification, arising from many experimental factors, such as instrumental setup, applied protocols, or databases, which highly affects the protein characterization and prevents quantitative comparison between different studies [84]. This fundamental problem has general validity and applies also to the top-down approach, which is similarly influenced by a number of experimental parameters.

In addition to the various experimental factors, data interpretation and bioinformatic analysis are also important aspects [75,84]. The basic concept for search algorithms fall into two broad classes: database-depending and *de novo*. A growing number of software and packages are now available for peptide/protein identification [85,86]. However, some tools remain challenging for inexperienced end-users due to lack of appropriate documentation or poor graphical user interfaces, and show a limited robustness for the output of the same proteomic dataset [87,88]. Experience in handling such proteomic software tools and in partially manual assessment of the data is therefore usually required to properly evaluate the analytical outputs. For all approaches, well-annotated genome and/or transcriptome data are an essential prerequisite to enhance the annotation performance of venom proteomes especially in understudied venomous organisms [89]. Although databases are still limited in terms of taxonomic coverage and do not include species-specific venom protein sequences, close evolutionary relationships within a particular taxonomic group allows to identify protein families of even totally unexplored venom organisms, reflected by protein sequence homology [49].

4.3 Future perspectives for high-throughput venom proteomics

Due to the limitations summarized above, the current gold standard and good practice for venom proteome analyses consists of application of both complementary proteomic approaches. An overarching future goal for venom proteomics studies is to improve the existing methods to allow faster and even more precise analyses of larger sample sets [49,80]. A top-down protocol, overcoming some of the aforementioned limitations, was recently developed [90]. This approach enables rapid and detailed profiling of multiple individual venom samples, along with statistical correlation tests for different factors, allowing population-scale analyses for a better understanding of regional and intraspecific venom protein variations.

Nonetheless, for high molecular mass toxin proteins (>30 kDa), current top-down analyses run into technical limits [81,91]. A future application to overcome these limitations in terms of ionization could be native electrospray ionization (nESI). However, native MS requires a specific platform with extended mass range, which is again associated with a loss of speed due to more extensive sample preparation, making this type of analysis still unfavourable for high-throughput [92,93].

The application of a hybrid element approach and molecular MS configuration is another powerful concept to decipher venom proteomes in its entirety. The parallel absolute quantification of µHPLC-separated intact sulfur-containing venom proteins by inductively coupled plasma (ICP) triple quadrupole MS and $^{32}S/^{34}S$ isotope dilution analysis, combined with bottom-up and top-down molecular MS, allow for both the exact quantification and the identification of proteins [73]. Another upcoming MS-based method that offers molecular information on the spatial distribution of toxins and new insights into the biology of venoms as well as their highly functionalized storage and delivery systems, is further discussed in section 8.

5. Transcriptome analyses of the venom system

The recent advances in high-throughput proteomics to analyse novel venoms are also fostered by the fast development of next generation nucleic acid sequencing technologies [49,68,94,95], see Figure 3. *De novo* venom protein analyses, as described above, depend on specific sequence databases of proteins to match masses of native or fragmented (novel) venom proteins. As many venom proteins, especially of unstudied species, are unknown, high-throughput mRNA sequencing (RNA-seq) of venom glands is often coupled to the proteomics analysis to provide a custom-based sample-specific database. RNA-seq represents consequently an important and growing core-pillar of venomics to describe the expression of venom genes and proteins even in the smallest venom systems as the required RNA quantities for library preparation range from 100 ng down to 1 ng [96]. Diverse workflows of RNA-seq (also for venomics) have been addressed and reviewed previously [49,94,97–101].

5.1 Advantages and challenges of transcriptomics

Several venomous animals harbour such minute venom systems that it is required to pool several specimens to obtain sufficient amounts of tissue material for RNA-seq. For some particularly small and difficult to rear organisms (remipedes, pseudoscorpions, smaller spiders etc.) the sensitivity of transcriptomics is indeed the last resort to grasp an idea of their supposed venom compositions because crude venom is difficult to obtain [94]. The downside of the sensitivity of modern RNA-seq is that, even if carefully prepared, venom system tissues can be contaminated by other body tissues; in addition, they also contain transcripts of proteins with normal, non-venom related functions [95]. The best practice is generally to avoid transcriptome-only studies, which should always be integrated with proteomic analyses - a strategy that is now commonly referred to as proteo-transcriptomics [94,95].

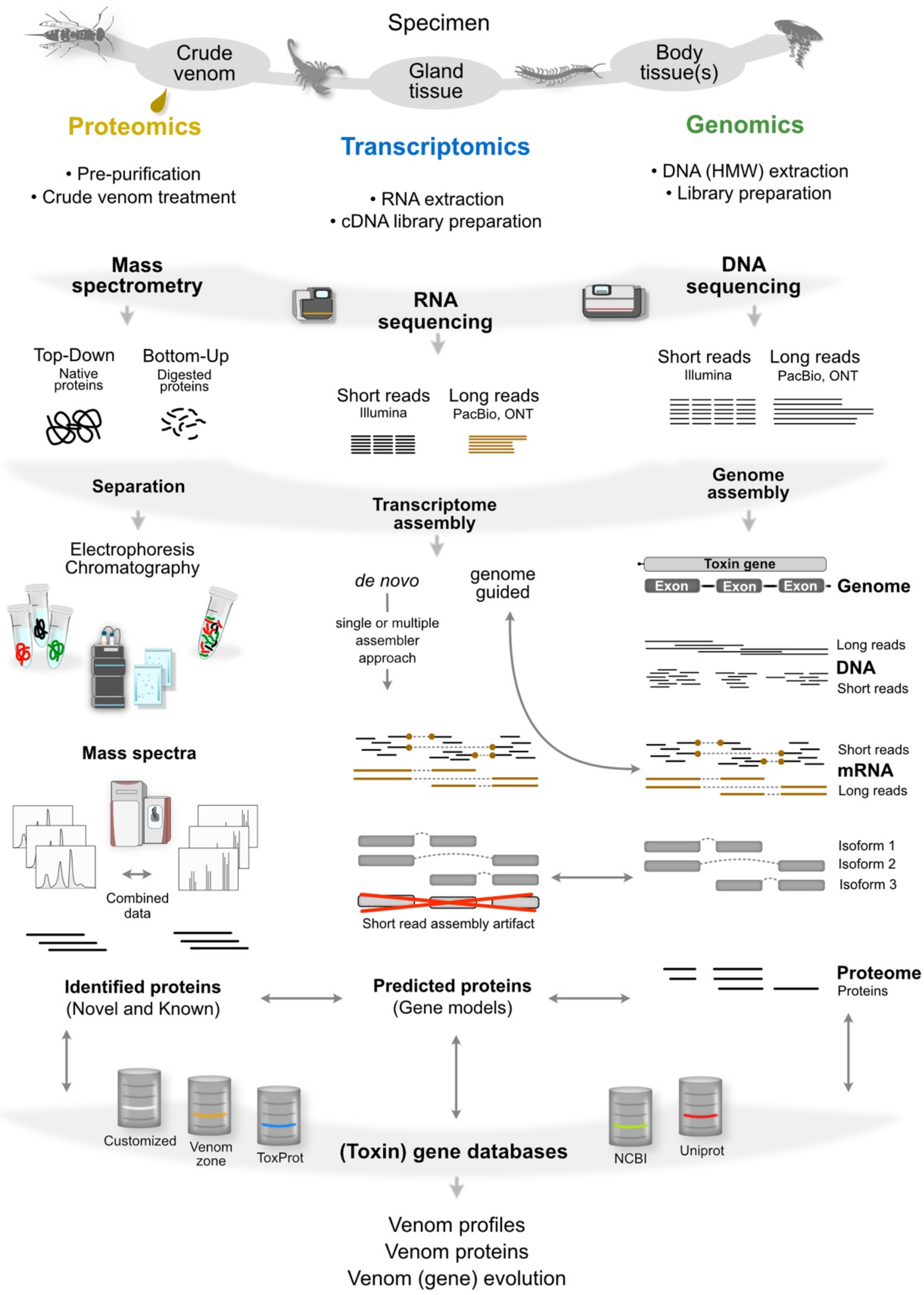

Figure 3: The integration of proteomics, transcriptomics and genomics in venom research.

For many species a physiological normalization of the venom system, for example by milking specimens at the same time to synchronize the replenishment cycle of their gland tissues, is

not applicable in the laboratory because milking, rearing or keeping them alive in the laboratory is difficult [94]. Examples are small solitary bees, marine remipedes, small spiders, marine molluscs and other species. As a consequence, many studies describe venom transcripts and venom gene populations as a snapshot, without the statistical power of differential gene expression analyses with multiple replicates applied in ecological and clinical studies [102,103]. Increasing the sample size of the specimen pool could level heterogeneity by including a larger mix of different 'wild-type' venom gland states.

5.2 Novel RNA-seq strategies

The specificity of assembly algorithms implies that diverse assemblers predict venom protein transcripts very differently and that single assembler approaches might underestimate transcript populations and isoforms [97,100,104,105]. As a consequence, the identification and prediction of proteins via MS might be affected when using these assemblies as specific databases. Recently developed *de novo* assembly packages for short read data generated by Illumina sequencing platforms thus apply multiple-assembler strategies that combine different assemblers and output a merged assembly [106–108]. New versions even include the annotation steps in the automatized process. One downside is that these programs currently require advanced bioinformatics expertise and often a familiarity with either virtual or physical, often Linux-based, environments, such as Docker or Conda. One future direction is to transform these approaches to more usable mainstream solutions and to link these to genome data to perform genome-guided transcriptome assembly and to foster more hands-on training of venom researchers in bioinformatics. Henceforward, direct RNA sequencing with novel sequencing platforms, such as ONT Nanopore or PacBio IsoSeq, with long reads and improved accuracy, will be increasingly applied, minimizing artificial transcripts or gene predictions [99,109].

The sequenced snapshots of expressed mRNA protein precursor molecules from tissue of the venom systems reveal not only transcripts of toxins and other venom proteins, but identify as well house-keeping genes that functionally guarantee the venom secretion. As a consequence, RNA-seq in combination with genome data and spatial -omics techniques is an important tool to reconstruct cellular pathways and mechanisms through which venom proteins and toxins are processed and translated [99]. A future direction will be to apply single cell RNA-seq (scRNA-seq) methods to differentiate expressed toxins in diverse gland cell populations to reveal spatial and temporal venom variations [103,110,111]. Single cell transcriptomics has been successfully applied to a variety of diverse animals including sponges, ctenophores, placozoans, cnidarians, planarians, nematodes, arthropods, ascidians, and vertebrates [112]. This breakthrough method simultaneously measures gene expression from thousands of individual cells. Clustering cells that share similar expression profiles allows for the identification and characterization of cell types that can be even more nuanced than traditional morphological characterizations. Cnidarians are a phylum typified by their venom-producing cells called nematocytes, whose biochemical and structural components have been successfully identified using scRNA-seq analysis [112–115]. Not only is this method capable of answering essential biological questions related to venom, but it is also capable of being implemented in non-model organisms. Unlike the use of transgenics to generate reporter lines and then sorting positive cells to generate a cell type specific transcriptome, virtually any non-model organism can now be explored at a cellular resolution. Beyond mRNA, other techniques can reveal genomic features at the cellular level. For example, ATAC-seq sequences portions of DNA to assess genome-wide chromatin accessibility and identifies key gene regulation mechanisms such as transcription factor binding sites [116]. Because ATAC-seq is highly sensitive it requires only minute amounts of chromatin and it can be employed to sequence even single cells, allowing the integration of transcriptomics and epigenomics at

cellular resolution [117]. Such insights into the gene regulation of venom secreting cells are essential to understand the evolution and development of venom systems (See also section 9.2).

5.3 Future perspectives of proteo-transcriptomics

Future directions for further developments of proteo-transcriptomics consist mainly in the development of more sophisticated and user-friendly data analysis strategies in combined, integrative interfaces. The most comprehensively assembled transcript libraries are generated with multiple-assembler pipelines including long read RNA-seq data and are ideally guided by available genome data. The predicted gene models and annotated protein genes are then used to identify proteins, using the outputs of mass spectrometry approaches in which bottom-up and top-down methods are applied to fragmented and native protein samples, see Figure 3. An even more holistic design is achieved if complementary spatial transcriptomics and mass spectrometry imaging methods are applied, see section 6.3.

6. Integrating molecular venomics with functional morphology

6.1 Challenges in connecting morphology, function, and molecular data

Beyond classical compositional and structural toxin analyses by proteo-transcriptomic approaches, in recent years there has been a steadily growing interest in the connection of these data to morphology, to elucidate the localization and mechanisms of venom toxins production, storage, and delivery [31,118]. Venom gland morphology is extremely variable: glands with a pronounced secretory function can have different numbers of cells, shapes and secretory modes [119]. Unicellular glands are mostly located in the epithelium of e.g., aquatic vertebrates, annelids and molluscs. Multicellular glands are usually located beneath the

epithelium. In terms of shape, glands can be defined as globular (acinous) or tubular. Composite glands can result from the association of several acinous and/or tubular glands [119]. Below the cuticle of arthropods, sunken uni- or multicellular glands are present that possess a specialized canal cell, which develops a conducting canal lined by a cuticle [120]. In terms of secretion mode, three types can be distinguished. In apocrine secretion, secretory grana or a liquid secretion is released. However, this secretion also contains organelles and mitochondrial as well as nuclear proteins [121]. In merocrine secretion, parts of the gland cells are released with the secretion. In holocrine secretion, the whole cell is released (e.g., in mammal sebaceous glands). As the loss of cell material in merocrine and apparently holocrine secretion is large, regenerative cells are present, e.g., in cnidocytes of Cnidaria or in the midgut of insects [119]. Thus, in different animal taxa glandular structures can range from single cells to large composite glands. Visualisation as well as anatomical analysis methods have to be chosen according to the level of interest, ranging from ultrathin sectioning to analyse subcellular anatomy to micro-CT analysis to visualize general gland morphology [122,123]. Novel technological innovations towards 4D tomography, which includes dynamic data from samples that undergo change during scanning, might enhance our functional understanding of venom systems [124]. Nevertheless, integration of molecular data in context of morphological or functional aspects are still challenging and the classical venomics approaches (proteomics, transcriptomics, genomics), used to examine spatial information of toxin production in various insect-feeding species, only allow limited resolution [5,111,125]. The glandular origin of the venoms in these studies were investigated by dissecting the secretory portions of the venom apparatus into a series of multiple segments and analysing respective sections by proteo-transcriptomic methods for variable toxin profiles [126–129]. Although macrodissection of venom glandular apparatus gives new insights into the biology

of venoms, it has several drawbacks including the laborious preparation, low resolution, loss of morphological structures and averaging effects across the section samples.

6.2 Targeted methods for the inference of spatial toxin distribution

As outlined above, the difficulties derived from the intrinsic nature of some venomous organisms and from the technological limitations of most commonly applied analysis methods, have hampered a comprehensive integration of functional, morphological, and molecular data. To obtain molecular information on the subcellular level, techniques such as in situ hybridization (ISH) and immunocytochemistry (ICC) have been used to map the spatial distribution of toxins and venom components directly on tissue sections [130,131]., These methods have demonstrated great potential in venom research, for instance, revealing previously unknown parts of the venom apparatus [132] or heterogeneity of toxin expression in venom glands [110,133]. However, these techniques allow to map only a few previously known targets simultaneously, providing limited molecular information.

6.3 Spatial Venomics: Non-targeted, high-throughput methods to visualize toxins

Advances in imaging technologies, proteomic analyses and high-throughput sequencing have facilitated the development of non-targeted techniques, such as mass spectrometry imaging (MSI) and spatial transcriptomics (ST), termed under the name ‘spatial venomics’. MSI has become popular in recent years and as a non-targeted approach, which is ideally suited to interrogate the spatial distribution of multiple toxin proteins, peptides or small molecules without prior knowledge of their identities [49]. The spatial resolution for different MSI instrumentation spans several orders of magnitude from 1 mm to 30 nm [134]. While several modes of ionization exist, MALDI remains the most appropriate for mapping proteinaceous

toxins within venom gland systems. To date MSI has been used to explore the distribution of venom components in a variety of venomous organisms including cnidarians, arthropods, and reptiles [118,135–137]. The MSI workflow in all studies acquires individual mass spectra in a regular raster (usually ~50 μm) across venom gland sections, which allow to display their distribution based on single toxins in a two-dimensional density map. Recently, a new approach, named functional MSI (fMSI), allowed to indirectly detect phospholipase A_2 (PLA_2) proteins by on-tissue enzymatic activity screening, which underlines the great potential of MSI for future *in-situ* approaches [136,138].

ST is a novel technology that allows the visualization and quantitative analysis of whole transcriptomes, creating gene expression maps within individual histological sections [139,140]. Tissue cryosections are placed on glass slides that contain an array of poly-T capture probes uniquely identified by spatial barcodes that allow to determine the origin of each mRNA molecule within the tissue. Therefore, ST allows to generate cDNA libraries with accurate positional information for RNA-seq, adding a spatial dimension to transcriptome data that enables analyses of gene expression within a morphological context. It is thus an ideal technique to investigate poorly known or challenging venomous organisms since it allows to identify toxin genes and their spatial expression patterns within the tissue, thus simultaneously characterizing the molecular composition of the venom and the morphological and functional organization of the venom producing tissue. Additionally, the sensitivity and high spatial resolution of up to 55 μm (equivalent to 5-10 cells) of the ST array, allows the study of very small venomous organisms while circumventing common obstacles encountered in bulk RNA-seq differential gene expression analyses. For instance, ST eliminates the need to pool small specimens losing the statistical power of biological replicates, and avoids contamination from tissues not related to venom production [94,95]. Furthermore, ST can also be combined with single-cell RNA-seq [141] offering the

possibility to simultaneously identify different venom secretory cell types and their specific spatial location in the venom system.

These spatial non-targeted methods allow us to conduct data-driven exploratory analyses without preselecting known targets of interest and are excellent tools to investigate cell types and tissues whose organization and functions are not well understood [142], such as many animal venom systems. These two technologies add a spatial dimension to venomics, revealing genes and proteins associated with morphological features, providing essential functional information about venom systems, from the genetic to the phenotypic level, from the molecular composition of the venom to the morphological features of the delivery system.

7. The significance of genomic data

Despite increasing availability of technologies for generating high-quality genomes, venomous animals are still under-represented in most databases and studies. In particular, comparative genomics studies on the origin and evolution of venoms are very sparse [143]. A major barrier that hinders comparative genomics is the reduced quantity of material obtainable from very small venomous organisms. However, developments in (ultra) low input protocols may aid in overcoming this hurdle, using amplification techniques. Novel methodologies also allow sequencing of difficult genomes of predominantly small marine invertebrates that are characterized by extensive production of mucus and/or other inhibitory molecules (e.g. nematodes, molluscs and others). These new methodologies are being exploited by a number of genome consortia that are connected under the umbrella of the Earth Biogenome Project [144], whose ultimate goal is to sequence, within the next decades, the genomes of all animal and plant species to better understand their evolution, ecology, adaptations and interconnections, and to safeguard - as last resort digitally - the threatened

biodiversity and bioresources on earth [144,145,145]. Linked to these efforts, the numbers of published high-quality, chromosome level genomes has already substantially risen, allowing for more comprehensive investigation of the origin and evolution of venom genes, predominantly from iconic groups such as snakes, spiders and cone snails [146–153].

7.1 Venom gene origin

Genome data is an important reference material to assess the accuracy of transcripts and gene models obtained from RNA-seq data by genome-guided transcriptome assembly approaches. However, high quality genomic data with good gene annotations are only obtained if multiple tissue samples are mapped on the genome and transcript-based gene predictions, improved by corresponding proteome data, are implemented [143,154], see Figure 3. Many available genomes lack a reliable gene annotation because they were automatically annotated [145,155]. Annotation with automated pipelines is prone to both false positive and false negative matches since venom genes belong in most cases to multi-gene families, often with high similarity of new toxin copies to their ancestral non venom-related paralogs [156]. Genomic data is likewise of utmost importance to identify ortholog genes (especially when short) and to compare venom genes to their non-toxic homologs in individual genomes. One future challenge is to improve the reliability and the speed of the process to predict genes in genomes. Without knowledge of the physical genomic location of a toxin encoding gene, it is very difficult to identify its orthologs in other species. Genomes also provide information on exons and introns that are crucial to gene structure evolution (Figure 4 A). For instance, gene duplication often results in incomplete sets of exons that can be used to trace back duplication events [149,156] that are impossible to detect otherwise [157]. At the same time, intronic sequences can provide a more reliable phylogenetic signal when genes evolve under

extremely strong positive selection [158]. For example, sometimes a toxin encoding gene can evolve from a non-toxic gene by deletion or gaining of exons [159].

The evolutionary history of toxin genes is more realistically reconstructed if their exact genomic location is identified using unrelated, syntenically conserved flanking genes, followed by location of that same genomic region in the outgroup species' genomes. After that, an exon screening (via BLAST or other sequence similarity tools) should take place to locate all related genes and pseudogenes in that region. A phylogenetic analysis of complete gene sequences subsequently helps to identify gene sub-clades. Previous knowledge of gene evolution can help to infer the most likely evolutionary history of a given gene [157,160–163,163]. Several helpful online and standalone software tools have been recently developed (e.g. SimpleSynteny, SynMap, AliTV [164–166]), however, they often rely on previously published *de novo* genomic annotations, which as explained above are particularly error-prone [155]. One direction for improvement of this step is to train the gene prediction with specific proteo-transcriptomic data from venom proteins (e.g. [154]. With the afore-mentioned genome sequencing initiatives we will soon be able to apply comparative genomics methods to detect the occurrence of convergent venom gene evolution in larger clades: the inclusion of many non-typical venom taxa is in fact crucial to infer general and lineage-specific patterns of gene evolution.

7.2 Venom gene manipulation by knock down and CRISPR

Advancements in available tools and techniques for genetic manipulations are currently growing among non-model species, including venomous animals. For instance, parental and embryonic RNA interference (RNAi) are regularly used to investigate the developmental biology of the common house spider, *Parasteatoda tepidariorum* [167,168]. The more

advanced CRISPR-mediated mutagenesis has been also developed for some model venomous species, for examples the jewel wasp, *Nasonia vitripennis* [169], the honeybee, *Apis mellifera* [170], and the red imported fire ant, *Solenopsis invicta* [171]. However, only in the cnidarian *Nematostella vectensis* genetic manipulations, including knockdowns using morpholinos and shRNA, as well as CRISPR-mediated techniques [172,173], have been employed to address venom-related questions, such as elucidating the factors associated with the biogenesis of venom-secreting cells [111,174], see Figure 4 B.

Transgenic approaches in *N. vectensis* have allowed the tracing of spatiotemporal dynamics as well as the localization of two distinct venom-secreting cells (nematocysts and gland cells [175,176]. Furthermore, specific toxins were found to be secreted in subpopulations of both cell types [175], adding a new level of complexity lacking in previous analyses. Further, by incorporating fluorescent markers into the structural components of venom-secreting cells using CRISPR/Cas9 techniques followed by FACS sorting of different types of nematocytes, different types of stinging cells were isolated [177]. RNA sequencing of the isolated cells revealed numerous differentially expressed genes, including some transcription factors resulting from lineage-specific duplication and essential for proper cnidocyte differentiation [177].

While these techniques have been instrumental in elucidating the structural components of the venom system, the characteristics of toxin components remain largely unresolved. Particularly of interest is the ability to genetically manipulate toxin-encoding genes in animals and test the impact on the fitness of mutants. Examples of such studies may include the deletion of a functional toxin before exposing the mutant to native predators and prey, to test whether defense and predation abilities are affected.

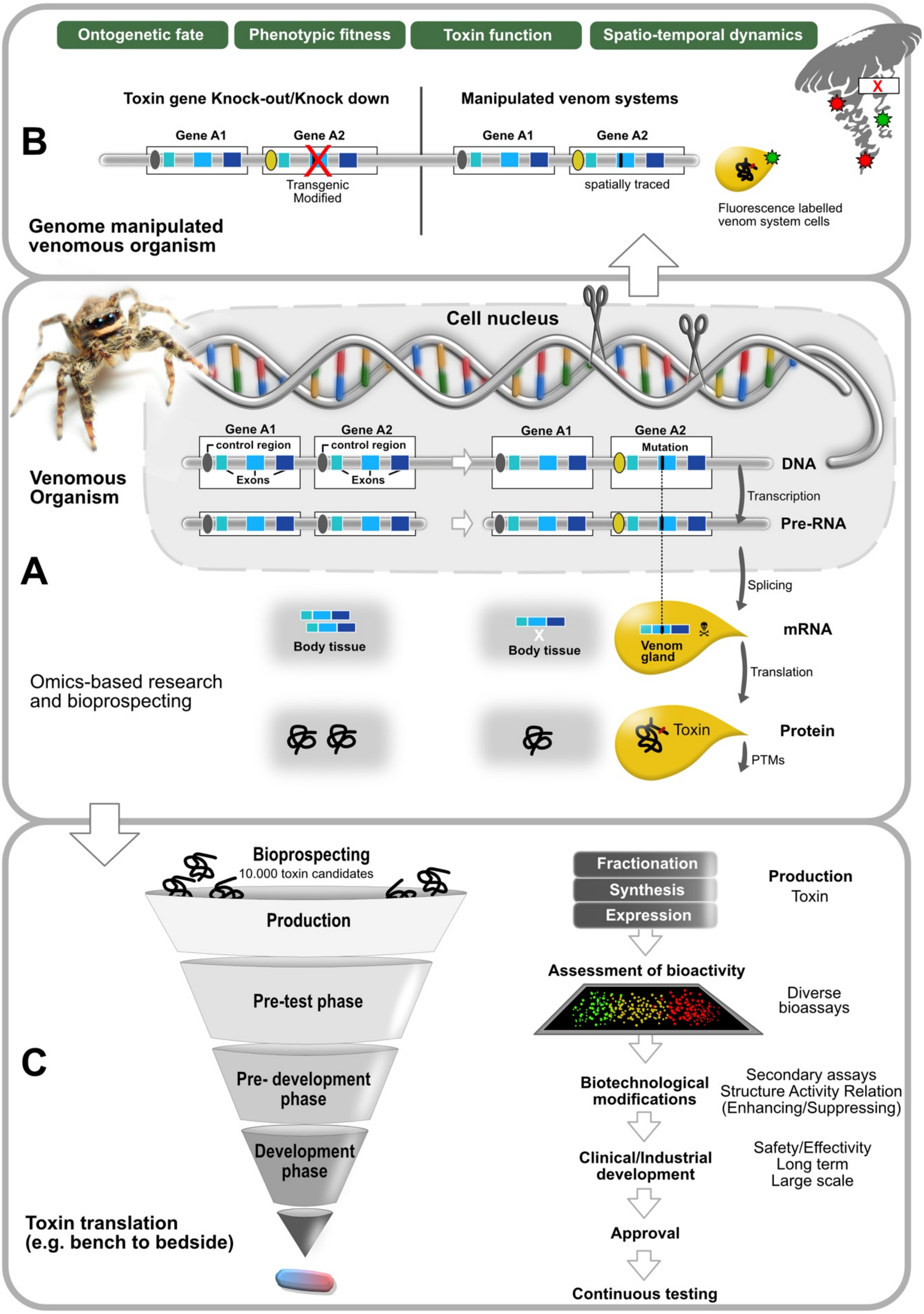

Figure 4: The integration of -omics based research to improve translational research but also our basic understanding of venom and toxin gene evolution. A) Shows the biological process from gene to protein; B) illustrates genome editing aspects

to investigate toxin evolution, function, adaptive value, spatio-temporal variability and ontogenetic fate.; C) Summarizes the major steps in translational research, from bioprospecting to application. PTMs = Posttranslational modifications.

A further expansion of this approach would be deleting multiple different toxins followed by subsequent mutants' crossings to produce individuals completely lacking venom. Additional assays could include knock into the animal's genome ('gene knockin') additional toxin domains, to cause overexpression of a toxin, or introduce precise modifications of single nucleotides to recapitulate an ancestral venom profile. The recent development of organoids from snake venom glands represent a new opportunity to test *in vitro* genetic manipulations [110]. Although this technology will need further developments to be easily applied to other systems, it may provide opportunities to simultaneously knock down toxin genes or edit regulatory regions to perform functional studies.

8. Production of venom components

8.1 Challenges of isolation-based venom biodiscovery

Functional characterization of toxins isolated from venom can be conducted directly using the purified peptide or protein. However, the small size of many venomous species, in particular invertebrates hinders the mechanical manipulation of the venom system and/or the collection of a sufficient amount of venom from a single specimen [6]. In these cases, an extraordinarily large number of specimens must be sampled to accumulate sufficient venom for the isolation of single compounds, raising ethical concerns [6,94,178]. In these and other cases where toxin sequences can be identified only *in silico* (Transcriptome or genome-based, see figure 3 and 6.1), methods of chemical synthesis and recombinant expression, which can produce milligram amounts of single venom components, are becoming increasingly important (Figure 4).

8.2 Chemical synthesis

Chemical synthesis is ideally suited for relatively short peptides (<50 residues) and requires prior knowledge about the peptide sequence, which must be obtained by MS or other methods (Edman degradation, novel NMR-based methods) on the isolated natural toxin or from genome or transcriptome sequencing. In many cases, additional knowledge about the disulfide pattern (as well as other PTMs) is required to ensure the correct native folding of the produced toxin.

While not suited for larger venom proteins, this approach has been instrumental in the functional and structural characterization of toxin peptides. The most common method applied is solid-phase peptide synthesis that can produce quite large amounts of peptide (generally mg or g, but kg yields are possible in industrial settings). Advantages of chemical synthesis include the ability to incorporate e.g., unnatural amino acids, D-amino acids, reporter groups, and unusual PTMs (such as brominated tryptophan) that are impossible to produce by recombinant production, as well as the regio-selective formation of disulfide bonds and cyclization [180].

8.3 Recombinant production

With an increasing number of both prokaryotic and eukaryotic expression systems that support the production of post-translationally modified proteins, recombinant production of toxins is also becoming more accessible. Given that most toxins are endogenously produced in the endoplasmic reticulum of the host and that PTMs can play crucial roles for toxin activity [181–183], eukaryotic host cells for recombinant production generally provide the best chance of producing functional toxins. Most common eukaryotic host systems used for toxin production include the yeast *Pichia pastoris*, insect cells and a variety of mammalian cell lines such as HEK293 and CHO. Although prokaryotic, the bacterial host *Escherichia coli* has been used to express thousands of toxins [184,185], and can yield mg amounts of toxin in a standard

laboratory setting. However, it is prone to a major drawback, the inability to add common PTMs such as glycosylation, C-terminal amidation and hydroxylation. The availability of a variety of systems, including specialized strains can allow for the production of disulfide-bound toxins in *E. coli* (see below). Regardless of the specific expression host, recombinant expression offers the advantage of incorporation of affinity purification tags, the ability to easily produce a large number of variants for functional testing and to obtain proteins that are substantially larger than those commonly made by chemical synthesis.

8.4 Future perspectives of toxin production

In vitro refolding of chemically synthesized peptides is often inefficient, especially for peptides containing three or more disulfide bridges. Based on sequence homology with characterized toxins, the disulfide pattern may well be deduced and thus allow for directed folding strategies. However, for novel, previously uncharacterized sequences, recombinant production may be the best suited or unique option, and as such has been subject to remarkable developments in recent years. To allow disulfide bond formation in *E. coli*, a variety of methods and strains already exist and many have been employed for toxin production [186–191]. Recently, new systems have been introduced [192] and the ability to produce thousands of disulfide-bonded animal toxins in *E. coli* has been demonstrated in important studies from the Vincentelli lab [185,193,194]. Hundreds of cystine-dense peptides containing up to five disulfide bonds have recently been produced in HEK293 for e.g. structural characterization [195]. Moreover, the same expression system has been used for surface display, which allowed screening of thousands of toxin peptide sequences to identify strong peptide interactors for specific targets [196]. This work demonstrated the potential of cystine-dense peptides to function as binders for transmembrane targets that are otherwise difficult to inhibit. Finally, cell-free synthesis approaches that have been used sporadically to produce venom toxins [197,198] have a great

potential for further developments. It is imperative to scale up such techniques to fulfill the potential of the many new sequences now available. The infrastructure needed to produce thousands of peptides is often not available in an average (academic) lab setting. Such undertakings will therefore require larger publicly funded consortia and/or closer collaborations between academia and industry than currently existing.

9. Applied and translational venom research

After sufficient quantities of crude venom or venom fractions are obtained and/or suitable amounts of a single compound are produced (see sections 2 and 10) their applicative potential can be assessed through a variety of bioassays. This translational perspective of animal venoms has always been a major driver of venom research. However, a large gap remains between the plethora of described animal venom compounds and the much fewer approved drugs, bioinsecticides, pharmacological and cosmeceutical products based on animal toxins [2,10]. Most candidates are dropped during the trial phase, while the few remaining ones have to be adapted biotechnologically to enhance or improve their properties. Both the testing and the optimization steps are expensive and time-consuming, being a major hurdle for applicative developments. The insights from the beforehand discussed -omics methods allow now a far more efficient and targeted bioprospecting that increases also the chances to realistically identify promising candidates and to reduce the number of unsuccessful candidates.

9.1 Bioassays in pharmacology

Bioactivity assay systems have been developed over many years to characterize the mechanism of action and pharmacological properties of venoms, and have been constantly improved to reveal novel targets [199–201]. In particular, the complexity of venoms is

mirrored by an ever-increasing number of bioassays developed to characterize their structural, functional, and pharmacological properties. These bioassays span from *in-vivo* phenotypic screens, to *ex-vivo* and *in-vitro* models as well as *in-silico* analyses (Figure 5). These approaches are mostly pursued from two complementary perspectives: 1) Basic biological characterization of a given venom leads to description of behavioural phenotypes and the identification of underlying cellular and molecular targets. 2) Target-specific assays may be used to identify venoms and toxins interacting with the molecular target(s) of interest [202,203].

Injection into an organism *in-vivo* can mimic many aspects of naturally occurring bites or stings, thereby reflecting the complexity of physiological and behavioural phenotypes [201]. Whole-organism phenotypes and *in-vivo* assays offer a particularly powerful approach when combined with the use of transgenic animal and transient knock-down/expression systems to test putative mechanisms of actions of a venom [175,204].

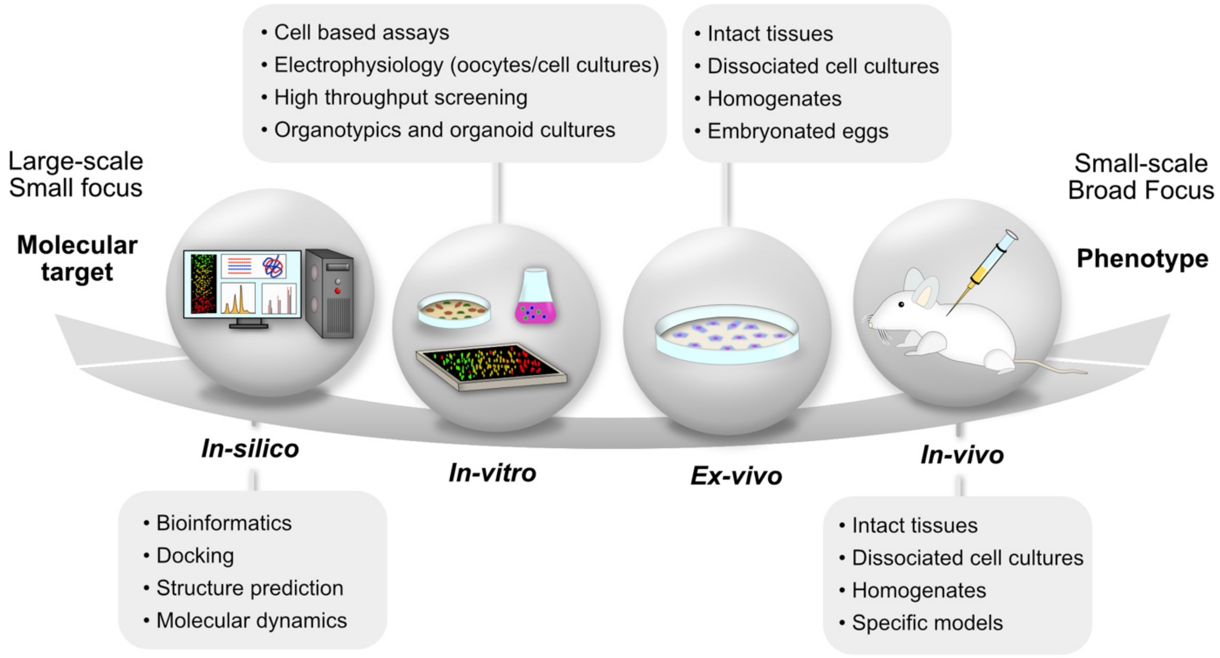

Figure 5. Approaches to study the activity of venom components span from *in-vivo, ex-vivo, in-vitro,* to *in-silico* methods. This allows the characterization of a broad spectrum of physiological effects, from whole organism phenotype to molecular target.

In-vivo assays

Despite *in vivo* assays on vertebrates pose ethical limitations, are labourintensive, and often not scalable to high-throughput approaches [203], murine models are widely used in basic and applied venom research for the characterization of effective and/or lethal doses (ED_{50}, LD_{50}). These assays also remain a prerequisite for the clearance of novel therapeutic agents by most regulatory agencies [205]. High-throughput chemical screens are performed using zebrafish embryos and chemical libraries, taking advantage of several available transgenic lines and disease models. Other potential organisms such as *Drosophila* have also been used in venom-based research with promising outcomes [206].

Ex-vivo assays

The complexity of *in vivo* approaches can be reduced by experimenting on representative *ex-vivo* tissues. Tissues extracted from mice, frogs, electric eels, chicken, and other organisms, were instrumental for laying some of the most fundamental cornerstones of basic physiology studies of venom research [203]. *Ex vivo* methods reduce and refine animal use since multiple samples can be established from a single sacrificed animal. In addition, they offer a more precise control of experimental conditions, allow convenient access for microscopy and biophysical probes, and facilitate the study of venom-induced effects on specific cell types even on subcellular structures and organelles. Similar to *in-vivo*, transgenic or transient transfection/transduction may help to elucidate molecular mechanisms of action. However,

freshly isolated tissues from animals may not be well suited for high-throughput analysis or for studying the toxin function at a single protein level [203,204].

***In vitro* assays**

Broad functional studies can be performed *in vitro* in cell lines, which allows precise system compositions, efficient pharmacological access, genetic manipulation for knock-out/down, knock-in and mutagenesis as well as the use of reporter systems [207,208].]. Immortalised cell lines provide valuable insights into the therapeutic potentials of animal venoms and their components as drug candidates. In addition, primary cells obtained from oocytes of the South African clawed frog *Xenopus laevis* allow exogenous expression of functional ion channels for electrophysiological analysis to evaluate their interactions with venoms and toxins [209,210]. Miniaturization and sensitivity are ever increased to characterize minimal amounts of venom components. Multiwell-plate assays have facilitated high-throughput screening of venom components against cells, enzymes, receptors, and ion channels, many of which are approved drug targets [203,211]. *In-vitro* biophysical methods offer the ability to manipulate both toxins and receptors at a molecular level and record the resulting effects with high spatial and temporal precision [212]. *In-vitro* studies of venom cannot replace *in-vivo* experimentation due to the inability to reflect the full physiological complexity, however, they are important to reduce and refine the animal use by providing mechanistic insights to allow focused and informative *in vivo* experiments [213].

***In silico* assays**

Modelling of toxin interaction with cell-membrane receptors *in silico* has emerged as a powerful novel approach for drug discovery [214] which requires detailed structural

information of both the toxin and its receptor protein. Structures of numerous toxins derived from animal venom were determined using X-ray crystallography or NMR spectroscopy in the 1970s and 1980s [215,216]. In contrast, nearly 80% of all membrane proteins with known structures were determined only in the past decade, owing to the "resolution revolution" in electron-microscopy technology and the development of advanced crystallographic techniques [217], which has provided numerous structures of venom peptides in complex with their cell-membrane receptors [218–221]. Atomistic simulations of toxin-receptor interactions currently rely on two complementary methods, namely, docking and molecular dynamics [222], which may provide realistic representations of the system under study when combined [223]. Molecular dynamics trajectories can capture intricate details such as ion permeation events, binding/unbinding of the toxin, conformational changes of the receptor, and various protein-lipid and protein-solvent interactions at the atomic level [224–226]. The ever-growing computing power available for research facilities establishes *in-silico* approaches as central parts of venomic analysis pipelines. AI-driven structure predictions provide increasingly high-quality structural models and will gain importance for elucidation of toxin receptor interactions and accelerate the discovery of new promising venom-based therapeutic lead structures [227].

Critical and future aspects on current bioassays

In- silico approaches cannot substitute experimental research, but rather complement it by providing powerful predictions to direct subsequent *in vitro, ex vivo,* and *in vivo* experiments. Refinement and miniaturization of test models in combination with recombinant and/or synthetic toxin production as well as organoid venom-glands [110] opens the door for powerful high throughput assays. *In vivo* animal high-throughput screening (HTS) can be carried out using model organisms such as flies, fishes, or nematodes [202]. *Ex-vivo*, high content

screening (HCS) microscopy has been established as an interesting novel single cell analysis of highly complex cell mixtures [211,228]. *In-vitro* high-throughput electrophysiology in mammalian cells and *Xenopus* oocytes is also gaining importance [203]. Interestingly, classical pharmaceutical *in-vitro* screening platforms such as fluorometric imaging plate reader (FLIPR), amplified luminescent proximity homogeneous assay (ALPHAscreen) and homogeneous time resolved fluorescence (HTRF) screens have so far only scarcely been applied to venom and its components [229,230]. It is anticipated that these methodologies combined with microfluidic approaches will be highly beneficial for venom research.

9.2 Pharmaceutical applications

Venom compounds have a wide spectrum of pharmacological applications, including analgesic, anti-inflammatory, antimicrobial and anti-cancer activities that have been used as prototypes for drug design and therapeutic agents and are utilised in a variety of therapeutical settings [2,199,231–234]. Currently 11 toxin-based molecules have been approved by the US Food and Drug Administration (FDA) or the European Medicines Agency (EMA), and are on the market [2,10]. These venom-derived drugs are used for the treatment of hypertension, acute coronary syndromes, coagulation during surgery, chronic pain, type 2 diabetes and perioperative bleeding, while many others are currently in clinical trials or in preclinical development. The original molecules were discovered predominantly in snakes (captopril, enalapril, tirofiban, eptifibatide, batroxobin, and cobratide), lizards (exenatide and lixisenatide) and several marine and terrestrial invertebrates from cone snails and leeches (e.g., ziconotide, bivalirudin and desirudin) [10,199,235–237]. However, critically it has to be noted that the whole process from bioprospecting to the final development of a compound for pharmaceutical applications remains challenging (Figure 4, C). In the following sections, we

discuss challenges and highlight biological and ecological traits of venomous species which could greatly improve the effectiveness of this process.

Targeting pain

Severe pain is often one of the main symptoms of envenomation, especially in defensive venom where toxins are instrumental in triggering aversive responses. This ability made venom toxins fundamental tools to investigate the physiology of nociception, which involves a number of receptors located in the peripheral nervous system, including the voltage-gated Na_v, K_v and Ca_v channels, and the ligand-gated transient receptor potential (TRP) channel, acid-sensing ion channel (ASIC) and P2X in the primary afferent neurons. AMPA (α-amino-3-hydroxy-5-methyl-4-isoxazolepropionic acid receptor), NMDA (glutamate-gated cation channels), NET (norepinephrine transporter) and GPCRs (G-protein-coupled receptors), together with Na_v and Ca_v, affect modulation of pain at the spinal level [238]. Generally, agonists of these channels in nature elicit pain and trigger aversive responses [239], while antagonist toxins are extremely promising as analgesic drugs and indeed their efficacy as antinociceptives has been demonstrated by multiple studies in murine models. This is the case of toxins from the sea anemone *Heteractis crispa* that act as selective TRPV1 modulators and show analgesic effects in acute and chronic pain models in mice without causing hyperthermia, a common side effect of other TRPV1 antagonists [240]. On the contrary, crotalphine from the South American rattlesnake induces a potent and long-lasting analgesic effect in mice by activating and thus desensitizing the ankyrin-type TRPA1, which plays a critical role in the pathogenesis of pain and inflammation [241]. Several spider, snake and sea anemone-derived toxins, including the well-characterized mambalgins, inhibit the activation of ASICs and areinvolved in different pain conditions [242–244]. A wide range of venom toxins target the voltage gated Na_v channels, which are crucial in electrical signalling and

neuromuscular function. Activators induce rigid paralysis and pain, while inhibitors are able to elicit spastic paralysis and analgesia, in both cases with a remarkable predatory and defensive effectiveness. Among them, the inhibitory cysteine knot (ICK) peptides, produced by spiders, scorpions, and cone snails, have been particularly studied [245,246]. Some ICK peptides also act as blocker of Ca_v channels including the cone snail o-conotoxin MVIIA (Prialt), an FDA-approved analgesic for spinal administration in severe chronic pain [247]. Relatively few classes of toxins target GPCRs, including conotoxins that are active against visceral and post-surgery pain through different mechanisms involving $GABA_B$ and k-opioid receptors, NMDA and NET [248,249]. Others are snake and spider toxins that modulate P2X and AMPA receptors to reduce inflammatory pain [250]. Overall, a variety of venom toxins have a great potential to develop novel analgesics that are able to block pain at its source [251].

Anticancer applications

Anticancer properties of animal toxins which manipulate signalling cascades controlling cell death and tumour growth, are promising therapeutics [233,252–256]. In particular, peptides from spiders and octopus and the crude venom of various snake species (cobras and vipers) have recently been reported to target specifically human melanoma often with minimal effects on healthy fibroblast cells [255–259]. Other anticancer activities of animal venomshighlight their potentials by inhibiting the proliferation and invasion of cancer cells, through cell cycle arrest and/or induction of apoptosis, as well as by revealing the affected signalling pathways [233,252,260,261]. However, potent venoms with anticancer activities many times raise concerns regarding their toxicity in healthy, non-targeted cells and tissues [262]. These could be overcome by directly targeting tumour cell (e.g., nanoparticle-based delivery systems). In addition, combination approaches, using venom or the active compound

coupled with existing chemotherapeutic agents at a low dose [253,262,263]. However, toxicities that emerge by the combination still need to be evaluated along with the observed anticancer or other therapeutic potential.

Immunomdulation

The potential immunomodulating abilities of venoms and toxins have also started to receive attention [258,264,265]. Immunosuppressive activity has been demonstrated in snake crude venoms. In particular, the activity of the Red-Bellied Black Snake *Pseudechis porphyriacus* venom might translate to therapeutic applications for T cell-associated conditions including rheumatoid arthritis and inflammatory bowel disease [266]. Venom components from the rattle snake *Crotalus durissus terrificus* diminish specifically T cell proliferation and IL-2 production [267]. They induce a shift in the colonic microenvironment from proinflammatory to anti-inflammatory in mouse models of induced colitis reveals [268]. These effects have been linked to the action of several specific toxins, belonging to different classes, including PLA2, cysteine rich secretory proteins (CRISPs), metalloproteases, serine proteases, L-amino acid oxidases (L-AAOs). In addition, many invertebrate venoms have been employed by traditional medicine in different cultures to treat, among others, autoimmune diseases, from bees to scorpion (Hwang et al., 2015; Ortiz et al., 2015). Recent studies have confirmed that the two major bee venom components, melittin and apamin, regulate respectively Th2 cell-mediated responses (An et al., 2018) and the production of monocytes and macrophages (Kim et al., 2012). On the other hand, both margatoxin from *Centruroides margaritatus* scorpions and the stichodactyla toxin (ShK) from the anemone *S. helianthus* are able to selectively block the $K_V1.3$ channel (Ortiz et al., 2015), a key component in autoimmune disease progression, highly expressed in effector memory T cells (Jimenez et al., 2017).

Antimicrobial activity

In the context of the current antibiotic crisis and the scarcity of therapeutic alternatives for the treatment of bacterial infections caused by multi-resistant bacteria or to treat viral infections, the search for new therapeutic alternatives is one persisting challenge. Antimicrobial peptides (AMPs) derived from animal venoms are biologically active cationic, anionic, or amphipathic peptides of less than 100 amino acid residues with a wide structural but stable range (alpha-helices, beta-sheets, extended structures, or disordered loops) [269,270]. In this sense, AMPs and other metabolites obtained from animal venoms are the future to develop a new generation of synthetic antimicrobial molecules with improved antibacterial, antiviral activity, safety, and broader spectrum of activity [269,271]. There are multiple examples of approved or promising AMPs described from various taxa such as Serrulin or Androctonin from scorpions, Melittin and derivatives from bees or L-AAO from snakes to mention a few [272–275], see Supplementary Tables S2-S4.

9.4 Pore forming toxins in sensing applications

One of the most interesting recent applications of venom proteins is their use in nanopore biosensing, which allows detection of various small molecules, peptides, proteins, DNA and RNA, sequencing, and analysis of enzymatic reactions at the single-molecule level (Figure 6, A). Although prokaryotic channels or pore-forming proteins are most commonly used for nanopore biosensing [276], cytolytic venom proteins are also very attractive candidates due to some advantageous properties [277]. These include channel stability, ease of insertion into artificial hydrophobic supports [278], and the ability to alter channel size through mutations that affect oligomerization. [279]. Although the current nanopore sequencing setup of Oxford Nanopore Technologies involves a prokaryotic transport channel [280], it is foreseeable that

venom protein channels will be developed for use in minION devices, whether for long-read sequencing or other biosensing applications (Figure 6, B).

Using the classical patch-clamp method, channels formed by a toxin from the sea anemone *Actinia fragacea* have been used so far to detect DNA, peptides, proteins, and small molecules [281–283]. However, nanopore biosensing may require more or less extensive mutagenesis of protein residues to enable analyte capture or translocation. Prior structural and biochemical knowledge is therefore paramount for the adaptation of venom proteins for biosensing experiments. It is important to note that nanopore-based identification and sequencing is performed at the single-molecule level, which means that it can enable the discovery of new or rare (macro)molecules and create opportunities for the development of highly sensitive diagnostic devices.

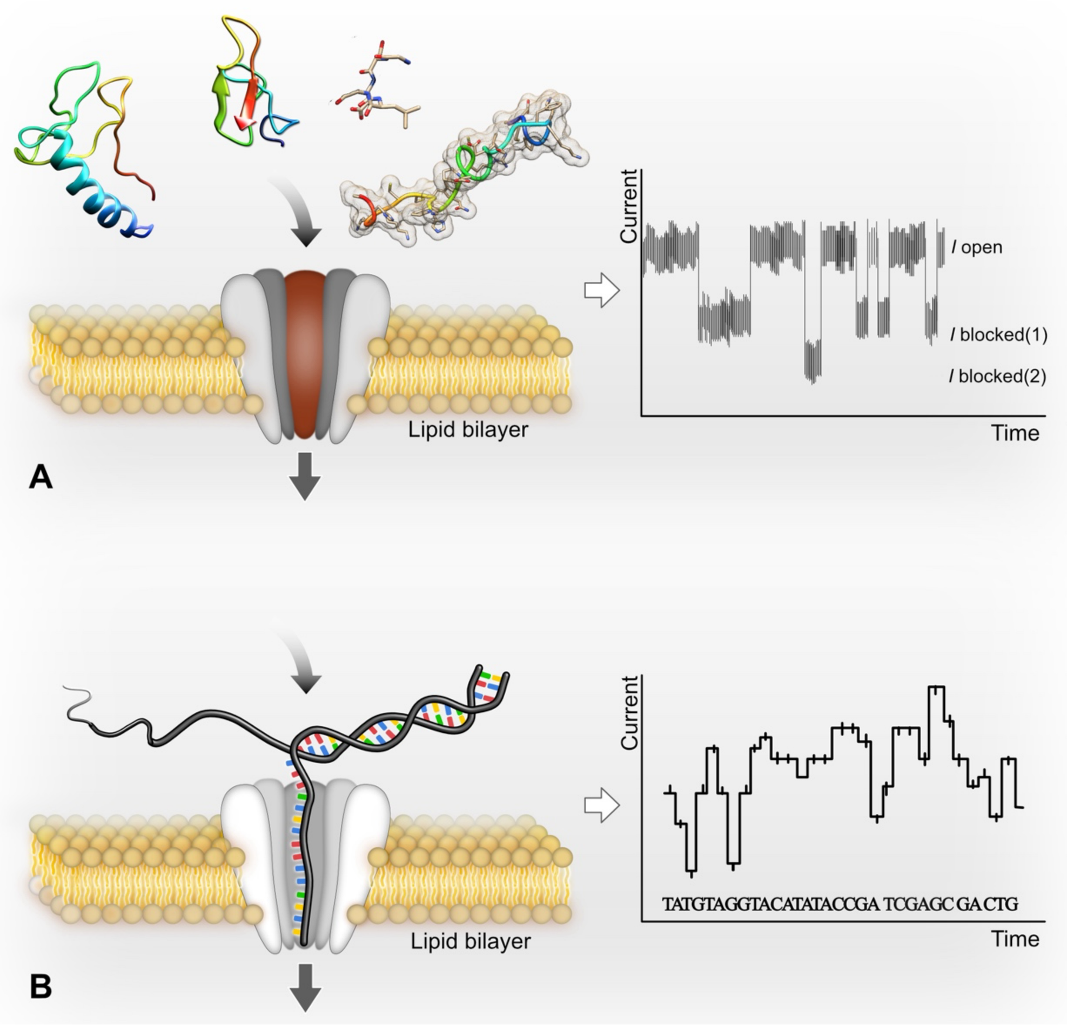

Figure 6. Schematic representation of nanopore biosensing. **(A)** Nanopore biosensing uses minute changes in electric current caused by the translocation of an analyte through the pore; each analyte is characterized by the percentage of current blockage and its duration. **(B)** The most widely used application of nanopore biosensing is DNA/RNA sequencing. The current trace is adapted from [284].

9.5 Agrochemical applications

The main approach to control pest species in agricultural and public health contexts relies on chemical pesticides. However, the continuous use of specific classes of insecticides has inevitably led to resistance in various pest species. In addition, current pesticides have a devastating impact on biodiversity [9,285–289] and have often raised concerns regarding human safety: due to improved health legislations many previously successful insecticides were de-registered (King and Hardy, 2013).

Altogether, these circumstances led to a renewed interest for the development of novel, eco-friendly bioinsecticides. Animal venoms, especially from predators that feed on insects, may be extremely promising for identifying novel natural insecticides, with strict species-specific action. The venom-derived insecticidal compounds tested to date have revealed a rich repertoire of bioactive compounds that specifically target ion channels of prey insects [9,290,291].

Novel spider peptides derived from the African and Australian Theraphosidae spiders as well as the African *Augacepahuls ezendami* have shown insecticidal capabilities for further development [292–294]. In line with this research approach, in 2017 the US-based company Vestaron launched the first peptide-based pesticide based on a knottin from a funnel web spider, thus validating the immense potential of animal venom-derivatives as bioinsecticides. The innovative aspect of this knottin is that it is sprayed on plants and then orally taken in by pest species. This makes genetic modifications of plants obsolete after which they express the toxin genes that were incorporated into their genome [9].

9.6 Diagnostics

As a result of the extensive toxin investigations mostly from the late 1980's, several *in vitro* diagnostic tests were developed, commercialized and adopted as routine applications in hematology laboratories to be used for assessing hemostatic disorders [295–297]. Many hemostatic parameters such as fibrinogen breakdown products, activation/inhibition of various clotting factors, protein C activation, von Willebrand factor related disorders and lupus anticoagulants can be assayed by using snake venom proteins, mostly proteinases [297,298]. These tests have some advantages over other common assays with their unique mechanisms of action. For example, snake venom thrombin-like enzymes are generally not inhibited by thrombin inhibitors such as heparin, allowing to perform the test with the samples containing these inhibitors [295]. Detailed information about this topic can be obtained from cited references.

A more recent venom-based diagnostic toolwas developed from a species of scorpion, *Leiurus quinquestriatus* (death stalker). One of its major venom components, chlorotoxin which blocks chlorine channels, can also bind to matrix metalloprotease-2 (MMP-2), thatis specifically upregulated on the membrane of cancer cells, but not in normal cells. This unique feature has led to a diagnostic reagent, so-called “tumor paint” which can be used for monitoring tumors. Chlorotoxin peptide is labelled with a fluorescent cyanine dye, which when subsequently bound to cancer cells selectively helps to visualize the borders of tumor tissue precisely. This is particularly useful in treating brain tumors, as it is critical to be as precise as possible when exercising tumors during surgery to prevent irreparable brain damage. Chlorotoxin is additionally being evaluated in clinical trials as an *in vivo* diagnostic imaging agent for various cancers, including glioma [199,299] and recently granted a fast-track designation from USA Food and Drug Administration (FDA) for pediatric brain tumors. We predict that with more detailed knowledge on toxins and their distribution and developmental fate within the

venomous organisms further promising candidates with specific activities suitable for diagnostic applications will be identified.

9.7 Envenomation therapy: Antivenoms (in a nutshell)

Animal envenomation by several key taxa such as spiders, scorpions and snakes is a major public health concern worldwide, however, most dramatic are effects from snake bites. Millions of individuals are at risk due to their geographic location, which is inhabited by various lethal snakes, especially in Africa, the Middle East, India, Mexico, and South America [300]. Approximately 1.8 million people are annually bitten by snakes of which 138,000 people die due to envenoming while up to 500,000 snake bite survivors suffer from permanent physical or psychological disabilities worldwide [14,23]. As a consequence the World Health Organization included snakebite envenoming to the list of category A Neglected Tropical Diseases [301] and developed a strategy to reduce mortality and disability by 50% before 2030 [302].

Antivenom is the only specific and effective therapy for victims of envenomation. The active compounds reported are whole immunoglobulins G, their $F(ab')_2$ or Fab fragments extracted and purified from the hyperimmune plasma of large animals (mostly horse) and prepared by their immunization with a single venom or a mixture of several of them [303,304]. Unfortunately, we have a current, serious crisis in antivenom availability in such most endangered regions, like sub-Saharan Africa and tropical and sub-tropical Asia [305]. It is determined by cost, often scarce, and poor distribution because only a few countries are the antivenom manufacturers (and only three in Europe). In addition, it may require a cold-chain for transport and storage, which is problematic for rural areas of low-to-middle income countries (LMIC). Additionally, some major antivenom manufacturers (Syntex, Behringwerke and Sanofi Pasteur) have stopped antivenom production over the past two decades for

commercial reasons, creating a noticeable deficit of antivenom in the countries that they previously supplied, especially in Africa [14]. Even Europe faces current antivenom shortages, due to the low financial sustainability of their production and lack of compliance to good manufacturing practice (GMP) regulations. Further, recent analyses revealed the lack of comparative information on available antivenoms against European vipers [306–309] (e.g. Lamb *et al.*, 2017; Kurtović *et al.*, 2021), see Supplementary Tables S5 and cited references for more details.

Several promising new technologies have been presented in recent years for the manufacturing of therapeutic antibodies on an industrial scale as antivenoms. Some of these molecules include Mab (monoclonal antibodies), scfv (single chain fraction variable fragments) and nanobodies among others that could form the basis for future treatments [304,310]. However, to develop these new antivenomics platforms the most detailed knowledge of venom composition is crucial. The herein discussed methods and future perspectives facilitate an unprecedented understanding of the ecology and biology of venomous animals and their venoms which allows in consequence the production of more effective antivenoms.

Conclusions

- Fast advancements in genomics, transcriptomics and proteomics technologies increase our knowledge of convergently evolved venoms across the tree of life.
- A more detailed knowledge on toxins and their distribution and developmental fate within the venomous organisms will reveal new insights on their evolutionary origins while also identifying compounds with novel bioactivity and targets.
- Venom toxins possess a great translational potential, with applications in the therapeutic, diagnostic, agrochemical, and biosensing fields. More detailed biological

insights on venomous species facilitate a more targeted identification of new promising candidates with specific activities suitable for knownand novel applications.

- In particular, in the context of the current antibiotic crisis and the scarcity of therapeutic alternatives for the treatment of multidrug-resistant bacterial and viral infections, the search for new therapeutics is one persisting challenge that could be addressed by venom research.
- Due to their devastating impact on biodiversity and concerns for human safety, there is great interest in replacing conventional pesticides with eco-friendly bioinsecticides. Animal venoms, especially from predators that feed on insects, may be extremely promising for identifying novel natural insecticides, with strict species-specific action.
- The whole process from bioprospecting to the final development of a compound for translational applications remains challenging. The approaches here outlined combining multiple aspects of animal venoms, including the biological and ecological traits of venomous species, would greatly improve the effectiveness of this process.

Declarations

Additional Files

Supplementary Tables 1-5

Data availability

Not applicable.

Competing interests

The authors declare that they have no competing interest.

Funding

BMvR was funded by the Centre for Translational Biodiversity Genomics (LOEWE-TBG) in the programme "LOEWE – Landes-Offensive zur Entwicklung Wissenschaftlich-ökonomischer Exzellenz" of Hesse's Ministry of Higher Education, Research, and the Arts as coordinator of the group Animal Venomics initiated by Andreas Vilcinskas. BMvR and IK further acknowledge funding on venom research by the German Science Foundation to BMvR (DFG RE3454/6-1). AC, AV and GZ were supported by the European Union’s Horizon 2020 Research and Innovation program through Marie Sklodowska-Curie Individual Fellowships (grant agreements No. AC: 896849, AV: 841576, and GZ: 845674). MPI is supported by the TALENTO Program by the Regional Madrid Government (2018-T1/BIO-11262). TH’s venom research is funded by the DFG projects 271522021 and 413120531. LE was supported by grant #7017-00288 from the Danish Council for Independent Research (Technology and Production Sciences). NI acknowledges funding on venom research by the Research Fund of Nevsehir Haci Bektas Veli University (project numbers: ABAP20F28, BAP18F26). MIK and AP acknowledge support from GSRT National Research Infrastructure structural funding project INSPIRED (MIS 5002550). GA acknowledges support from the Slovenian Research Agency grants P1-0391, J4-8225, and J4-2547. GG acknowledges support from the Institute for Medical Research and Occupational Health, Zagreb, Croatia. E.A.B.U. is supported by a Norwegian Research Council FRIPRO-YRT Fellowship no. 287462. This work was supported by the European Cooperation in Science and Technology (CA COAT Action CA19144 – European Venom Network (EUVEN).

Author contributions

Lead, major conceptualization and graphics BMvR, all authors wrote the main text and edited the final manuscript. Except for the first author, authors are listed alphabetically with respect to the last name. All authors have read and agreed to the published version of the manuscript.

Acknowledgements

We like to thank Ronald Jenner and Stuart Ainsworth for commenting and editing the final manuscript version. MD is grateful to Prof. R. D. Süßmuth for the supervision and support during the PhD time, in which this manuscript was achieved. BMvR and IK thank Andreas Vilcinskas for support and work space at the Institute of Insectbiotechnology within the group Animal Venomics.

References

1. Holford M, Daly M, King GF, Norton RS. Venoms to the rescue. *Science*. 2018; doi: 10.1126/science.aau7761.

2. McDermott A. News Feature: Venom back in vogue as a wellspring for drug candidates. *PNAS*. National Academy of Sciences; 2020; doi: 10.1073/pnas.2004486117.

3. Fry BG, Roelants K, Champagne DE, Scheib H, Tyndall JDA, King GF, et al.. The toxicogenomic multiverse: convergent recruitment of proteins into animal venoms. *Annual Review of Genomics and Human Genetics*. 2009; doi: 10.1146/annurev.genom.9.081307.164356.

4. Casewell NR, Wüster W, Vonk FJ, Harrison RA, Fry BG. Complex cocktails: the evolutionary novelty of venoms. *Trends in Ecology & Evolution*. 2013; doi: 10.1016/j.tree.2012.10.020.

5. Schendel V, Rash LD, Jenner RA, Undheim EAB. The Diversity of Venom: The Importance of Behavior and Venom System Morphology in Understanding Its Ecology and Evolution. *Toxins*. Multidisciplinary Digital Publishing Institute; 2019; doi: 10.3390/toxins11110666.

6. von Reumont BM, Campbell LI, Jenner RA. *Quo vadis venomics*? A roadmap to neglected venomous invertebrates. *Toxins*. 2014; doi: 10.3390/toxins6123488.

7. Sunagar K, Morgenstern D, Reitzel AM, Moran Y. Ecological venomics: How genomics, transcriptomics and proteomics can shed new light on the ecology and evolution of venom. *Journal of Proteomics*. 2016; doi: 10.1016/j.jprot.2015.09.015.

8. King G. Venoms to Drugs. Royal Society of Chemistry;

9. King GF. Tying pest insects in knots: the deployment of spider-venom-derived knottins as bioinsecticides. *Pest management science*. 2019; doi: 10.1007/978-1-4899-1834-5_13.

10. Bordon K de CF, Cologna CT, Fornari-Baldo EC, Pinheiro-Júnior EL, Cerni FA, Amorim FG, et al.. From Animal Poisons and Venoms to Medicines: Achievements, Challenges and Perspectives in Drug Discovery. *Front Pharmacol*. Frontiers; 2020; doi: 10.3389/fphar.2020.01132.

11. Modica MV, Ahmad R, Ainsworth S, Anderluh G, Antunes A, Beis D, et al.. The new COST Action European Venom Network (EUVEN)—synergy and future perspectives of modern venomics. *GigaScience*. 2021; doi: 10.1093/gigascience/giab019.

12. Rodrigo C, Gnanathasan A. Management of scorpion envenoming: a systematic review and meta-analysis of controlled clinical trials. *Systematic Reviews*. 2017; doi: 10.1186/s13643-017-0469-8.

13. Pla D, Rodríguez Y, Calvete JJ. Third Generation Antivenomics: Pushing the Limits of the In Vitro Preclinical Assessment of Antivenoms. *Toxins*. Multidisciplinary Digital Publishing Institute; 2017; doi: 10.3390/toxins9050158.

14. Gutiérrez JM, Calvete JJ, Habib AG, Harrison RA, Williams DJ, Warrell DA. Snakebite envenoming. *Nat Rev Dis Primers*. 2017; doi: 10.1038/nrdp.2017.63.

15. Needleman RK, Neylan IP, Erickson T. Potential Environmental and Ecological Effects of Global Climate Change on Venomous Terrestrial Species in the Wilderness. *Wilderness & Environmental Medicine*. 2018; doi: 10.1016/j.wem.2017.11.004.

16. Dias-Lopes C, Paiva AL, Guerra-Duarte C, Molina F, Felicori L. Venomous Arachnid Diagnostic Assays, Lessons from Past Attempts. *Toxins*. Multidisciplinary Digital Publishing Institute; 2018; doi: 10.3390/toxins10090365.

17. Pucca MB, Cerni FA, Oliveira IS, Jenkins TP, Argemí L, Sørensen CV, et al.. Bee Updated: Current Knowledge on Bee Venom and Bee Envenoming Therapy. *Front Immunol*. Frontiers; 2019; doi: 10.3389/fimmu.2019.02090.

18. Linardich C, Brookson CB, Green SJ. Trait-based vulnerability reveals hotspots of potential impact for a global marine invader. *Global Change Biology*. 2021; doi: 10.1111/gcb.15732.

19. Giallongo G, Douek J, Harbuzov Z, Galil BS, Rinkevich B. Long-term changes in population genetic features of a rapidly expanding marine invader: implication for invasion success. *Biol Invasions*. 2021; doi: 10.1007/s10530-021-02521-8.

20. Wägele H, Klussmann-Kolb A, Kuhlmann M, Haszprunar G, Lindberg D, Koch A, et al.. The taxonomist - an endangered race. A practical proposal for its survival. *Front Zool*. 2011; doi: 10.1186/1742-9994-8-25.

21. Britz R, Hundsdörfer A, Fritz U. Funding, training, permits—the three big challenges of taxonomy. *MT*. 2020; doi: 10.11646/megataxa.1.1.10.

22. Coleman CO, Radulovici AE. Challenges for the future of taxonomy: talents, databases and knowledge growth. *MT*. 2020; doi: 10.11646/megataxa.1.1.5.

23. Casewell NR, Jackson TNW, Laustsen AH, Sunagar K. Causes and Consequences of Snake Venom Variation. *Trends in Pharmacological Sciences*. Elsevier; 2020; doi: 10.1016/j.tips.2020.05.006.

24. Ambler J, Diallo AA, Dearden PK, Wilcox P, Hudson M, Tiffin N. Including Digital Sequence Data in the Nagoya Protocol Can Promote Data Sharing. *Trends in Biotechnology*. 2021; doi: 10.1016/j.tibtech.2020.06.009.

25. UNEP-CBD Secretariat: Convention on Biological Diversity - The Access and Benefit-Sharing Clearing-House. Convention on Biological Diversity - The Access and Benefit-Sharing Clearing-House. https://www.cbd.int/ (2021). Accessed 2021 Nov 1.

26. Prathapan KD, Pethiyagoda R, Bawa KS, Raven PH, Rajan PD, 172 co-signatories from 35 countries. When the cure kills—CBD limits biodiversity research. *Science*. 2018; doi: 10.1126/science.aat9844.

27. Heinrich M, Scotti F, Andrade-Cetto A, Berger-Gonzalez M, Echeverría J, Friso F, et al.. Access and Benefit Sharing Under the Nagoya Protocol—Quo Vadis? Six Latin American Case Studies Assessing Opportunities and Risk. *Front Pharmacol*. Frontiers; 2020; doi: 10.3389/fphar.2020.00765.

28. Karger EJ, Scholz AH. DSI, the Nagoya Protocol, and Stakeholders' Concerns. *Trends in Biotechnology*. 2021; doi: 10.1016/j.tibtech.2020.09.008.

29. Fry BG, Undheim EAB, Jackson TNW, Georgieva D, Vetter I, Calvete J, et al.. Research Methods. *Venomous Reptiles and Their Toxins Evolution, Pathophysiology and Biodiscovery*. Oxford University Press; p. 153–214.

30. Low DHW, Sunagar K, Undheim EAB, Ali SA, Alagon AC, Ruder T, et al.. Dracula's children: molecular evolution of vampire bat venom. *Journal of Proteomics*. 2013; doi: 10.1016/j.jprot.2013.05.034.

31. Mailho-Fontana PL, Antoniazzi MM, Alexandre C, Pimenta DC, Sciani JM, Brodie ED, et al.. Morphological Evidence for an Oral Venom System in Caecilian Amphibians. *iScience*. 2020; doi: 10.1016/j.isci.2020.101234.

32. Harris RJ, Jenner RA. Evolutionary Ecology of Fish Venom: Adaptations and Consequences of Evolving a Venom System. *Toxins*. 2019; doi: 10.3390/toxins11020060.

33. Frederico A, Américo D, Stéphane B, Beatriz R, Francisco V, Joana R, et al.. A simple and practical technique for fish venom extraction - Protein content analysis for future biotechnological applications. *Front Mar Sci*. 2016; doi: 10.3389/conf.FMARS.2016.04.00124.

34. Saggiomo SL, Zelenka C, Seymour J. Relationship between food and venom production in the estuarine stonefish *Synanceia horrida*. *Toxicon*. 2017; doi: 10.1016/j.toxicon.2016.11.250.

35. Maček P, Senčič L, Lebez D. Isolation and partial characterisation of three lethal and hemolytic toxins from the sea anemone *Actinia cari*. *Toxicon*. 1982; doi: 10.1016/0041-0101(82)90189-1.

36. Kimura A, Nakagawa H, Hayashi H, Endo K. Seasonal changes in contractile activity of a toxic substance from the pedicellaria of the sea urchin *Toxopneustes pileolus*. *Toxicon*. 1984; doi: 10.1016/0041-0101(84)90079-5.

37. Kem WR, Parten B, Pennington MW, Price DA, Dunn BM. Isolation, characterization, and amino acid sequence of a polypeptide neurotoxin occurring in the sea anemone *Stichodactyla helianthus*. *Biochemistry*. 1989; doi: 10.1021/bi00434a050.

38. Purushottama G, Venkateshvaran K, Pani Prasad K, Nalini P. Bioactivities of extracts from the marine sponge *Halichondria panicea*. *J Venom Anim Toxins incl Trop Dis*. 2009; doi: 10.1590/S1678-91992009000300007.

39. Jouiaei M, Casewell NR, Yanagihara AA, Nouwens A, Cribb BW, Whitehead D, et al.. Firing the Sting: Chemically Induced Discharge of Cnidae Reveals Novel Proteins and Peptides from Box Jellyfish (*Chironex fleckeri)* Venom. *Toxins*. Multidisciplinary Digital Publishing Institute; 2015; doi: 10.3390/toxins7030936.

40. Dutertre S, Jin A, Vetter I, Hamilton B, Sunagar K, Lavergne V, et al.. Evolution of separate predation- and defence-evoked venoms in carnivorous cone snails. *Nature Communications*. 2014; doi: 10.1038/ncomms4521.

41. Hopkins C, Grilley M, Miller C, Shon KJ, Cruz LJ, Gray WR, et al.. A new family of *Conus* peptides targeted to the nicotinic acetylcholine receptor. *J Biol Chem*. 1995; doi: 10.1074/jbc.270.38.22361.

42. Gonçalves Paterson Fox E, Russ Solis D, Delazari dos Santos L, Aparecido dos Santos Pinto JR, Ribeiro da Silva Menegasso A, Cardoso Maciel Costa Silva R, et al.. A simple, rapid method for the

extraction of whole fire ant venom (Insecta: Formicidae: Solenopsis). *Toxicon*. 2013; doi: 10.1016/j.toxicon.2012.12.009.

43. Garb JE. Extraction of Venom and Venom Gland Microdissections from Spiders for Proteomic and Transcriptomic Analyses. *Journal of Visualized Experiments*. 2014; doi: 10.3791/51618.

44. Undheim EAB, Fry BG, King GF. Centipede venom: recent discoveries and current state of knowledge. *Toxins*. 2015; doi: 10.3390/toxins7030679.

45. von Reumont BM, Blanke A, Richter S, Alvarez F, Bleidorn C, Jenner RA. The first venomous crustacean revealed by transcriptomics and functional morphology: remipede venom glands express a unique toxin cocktail dominated by enzymes and a neurotoxin. *Molecular Biology and Evolution*. 2014; doi: 10.1093/molbev/mst199.

46. Walker AA, Rosenthal M, Undheim EEA, King GF. Harvesting venom toxins from assassin bugs and other heteropteran insects. *Journal of Visualized Experiments*. 2018; doi: 10.3791/57729.

47. Piek T. Methods for the collection of venoms. *Venoms of the Hymenoptera*. Academic Press; p. 45–54.

48. Aili SR, Touchard A, Petitclerc F, Dejean A, Orivel J, Padula MP, et al.. Combined Peptidomic and Proteomic Analysis of Electrically Stimulated and Manually Dissected Venom from the South American Bullet Ant *Paraponera clavata*. *J Proteome Res*. 2017; doi: 10.1021/acs.jproteome.6b00948.

49. Walker AA, Robinson SD, Hamilton BF, Undheim EAB, King GF. Deadly Proteomes: A Practical Guide to Proteotranscriptomics of Animal Venoms. *PROTEOMICS*. 2020; doi: https://doi.org/10.1002/pmic.201900324.

50. Jesupret C, Baumann K, Jackson TNW, Ali SA, Yang DC, Greisman L, et al.. Vintage venoms: proteomic and pharmacological stability of snake venoms stored for up to eight decades. *Journal of Proteomics*. 2014; doi: 10.1016/j.jprot.2014.01.004.

51. Klupczynska A, Pawlak M, Kokot Z, Matysiak J. Application of Metabolomic Tools for Studying Low Molecular-Weight Fraction of Animal Venoms and Poisons. *Toxins*. 2018; doi: 10.3390/toxins10080306.

52. Gorrochategui E, Jaumot J, Lacorte S, Tauler R. Data analysis strategies for targeted and untargeted LC-MS metabolomic studies: Overview and workflow. *TrAC Trends in Analytical Chemistry*. 2016; doi: 10.1016/j.trac.2016.07.004.

53. Lee DY, Bowen BP, Northen TR. Mass spectrometry—based metabolomics, analysis of metabolite-protein interactions, and imaging. *BioTechniques*. 2010; doi: 10.2144/000113451.

54. Hutchinson DA, Savitzky AH, Burghardt GM, Nguyen C, Meinwald J, Schroeder FC, et al.. Chemical defense of an Asian snake reflects local availability of toxic prey and hatchling diet. *Journal of Zoology*. 2013; doi: 10.1111/jzo.12004.

55. Aird S, Villar Briones A, Roy M, Mikheyev A. Polyamines as Snake Toxins and Their Probable Pharmacological Functions in Envenomation. *Toxins*. 2016; doi: 10.3390/toxins8100279.

56. Villar-Briones A, Aird S. Organic and Peptidyl Constituents of Snake Venoms: The Picture Is Vastly More Complex Than We Imagined. *Toxins*. 2018; doi: 10.3390/toxins10100392.

57. Acunha T, Nardini V, Faccioli LH. A lipidomics approach reveals new insights into *Crotalus durissus terrificus* and *Bothrops moojeni* snake venoms. *Arch Toxicol*. 2021; doi: 10.1007/s00204-020-02896-y.

58. Palma MS, Itagaki Y, Fujita T, Naoki H, Nakajima T. Structural characterization of a new acylpolyaminetoxin from the venom of Brazilian garden spider *Nephilengys cruentata*. *Toxicon*. 1998; doi: 10.1016/S0041-0101(97)00139-6.

59. Hisada M, Fujita T, Naoki H, Itagaki Y, Irie H, Miyashita M, et al.. Structures of spider toxins: Hydroxyindole-3-acetylpolyamines and a new generalized structure of type-E compounds obtained

from the venom of the Joro spider, *Nephila clavata*. *Toxicon*. 1998; doi: 10.1016/S0041-0101(98)00086-5.

60. Schroeder FC, Taggi AE, Gronquist M, Malik RU, Grant JB, Eisner T, et al.. NMR-spectroscopic screening of spider venom reveals sulfated nucleosides as major components for the brown recluse and related species. *Proceedings of the National Academy of Sciences*. 2008; doi: 10.1073/pnas.0806840105.

61. Forster YM, Reusser S, Forster F, Bienz S, Bigler L. VenoMS—A Website for the Low Molecular Mass Compounds in Spider Venoms. *Metabolites*. Multidisciplinary Digital Publishing Institute; 2020; doi: 10.3390/metabo10080327.

62. Lai L-C, Huang R-N, Wu W-J. Venom alkaloids of monogyne and polygyne forms of the red imported fire ant, *Solenopsis invicta*, in Taiwan. *Insect Soc*. 2008; doi: 10.1007/s00040-008-1025-2.

63. Chen L, Fadamiro HY. Re-investigation of venom chemistry of *Solenopsis* fire ants. II. Identification of novel alkaloids in S. invicta. *Toxicon*. 2009; doi: 10.1016/j.toxicon.2009.01.016.

64. Chen L, Fadamiro HY. Re-investigation of venom chemistry of *Solenopsis* fire ants. I. Identification of novel alkaloids in S. richteri. *Toxicon*. 2009; doi: 10.1016/j.toxicon.2008.12.019.

65. Pawlak M, Klupczynska A, Kokot ZJ, Matysiak J. Extending Metabolomic Studies of *Apis mellifera* Venom: LC-MS-Based Targeted Analysis of Organic Acids. *Toxins*. Multidisciplinary Digital Publishing Institute; 2020; doi: 10.3390/toxins12010014.

66. Klupczynska A, Plewa S, Dereziński P, Garrett TJ, Rubio VY, Kokot ZJ, et al.. Identification and quantification of honeybee venom constituents by multiplatform metabolomics. *Sci Rep*. 2020; doi: 10.1038/s41598-020-78740-1.

67. Torres VDO, Piva RC, Antonialli Junior WF, Cardoso CAL. Free Amino Acids Analysis in the Venom of the Social Wasp *Polistes lanio* Under Different Forms of Preservation. *Orbital: Electron J Chem*. 2018; doi: 10.17807/orbital.v10i1.1005.

68. Yates JR, Ruse CI, Nakorchevsky A. Proteomics by Mass Spectrometry: Approaches, Advances, and Applications. *Annual Review of Biomedical Engineering*. 2009; doi: 10.1146/annurev-bioeng-061008-124934.

69. Damm M, Hempel B-F, Nalbantsoy A, Süssmuth RD. Comprehensive Snake Venomics of the Okinawa Habu Pit Viper, *Protobothrops flavoviridis*, by Complementary Mass Spectrometry-Guided Approaches. *Molecules*. Multidisciplinary Digital Publishing Institute; 2018; doi: 10.3390/molecules23081893.

70. Bastos VA, Gomes-Neto F, Rocha SLG, Teixeira-Ferreira A, Perales J, Neves-Ferreira AGC, et al.. The interaction between the natural metalloendopeptidase inhibitor BJ46a and its target toxin jararhagin analyzed by structural mass spectrometry and molecular modeling. *Journal of Proteomics*. 2020; doi: 10.1016/j.jprot.2020.103761.

71. Mouchbahani-Constance S, Sharif-Naeini R. Proteomic and Transcriptomic Techniques to Decipher the Molecular Evolution of Venoms. *Toxins*. Multidisciplinary Digital Publishing Institute; 2021; doi: 10.3390/toxins13020154.

72. Calvete JJ, Lomonte B, Saviola AJ, Bonilla F, Sasa M, Williams DJ, et al.. Mutual enlightenment: A toolbox of concepts and methods for integrating evolutionary and clinical toxinology via snake venomics and the contextual stance. *Toxicon: X*. 2021; doi: 10.1016/j.toxcx.2021.100070.

73. Calvete JJ, Pla D, Els J, Carranza S, Damm M, Hempel B-F, et al.. Combined Molecular and Elemental Mass Spectrometry Approaches for Absolute Quantification of Proteomes: Application to the Venomics Characterization of the Two Species of Desert Black Cobras, *Walterinnesia aegyptia* and *Walterinnesia morgani*. *J Proteome Res*. 2021; doi: 10.1021/acs.jproteome.1c00608.

74. Lomonte B, Calvete JJ. Strategies in “snake venomics” aiming at an integrative view of compositional, functional, and immunological characteristics of venoms. *Journal of Venomous Animals and Toxins including Tropical Diseases*. 2017; doi: 10.1186/s40409-017-0117-8.

75. Dupree EJ, Jayathirtha M, Yorkey H, Mihasan M, Petre BA, Darie CC. A Critical Review of Bottom-Up Proteomics: The Good, the Bad, and the Future of This Field. *Proteomes*. Multidisciplinary Digital Publishing Institute; 2020; doi: 10.3390/proteomes8030014.

76. Slagboom J, Kaal C, Arrahman A, Vonk FJ, Somsen GW, Calvete JJ, et al.. Analytical strategies in venomics. *Microchemical Journal*. 2022; doi: 10.1016/j.microc.2022.107187.

77. Melani RD, Goto-Silva L, Nogueira FCS, Junqueira M, Domont GB. Shotgun Approaches for Venom Analysis. In: Gopalakrishnakone P, Calvete JJ, editors. *Venom Genomics and Proteomics*. Dordrecht: Springer Netherlands;

78. Huang T, Wang J, Yu W, He Z. Protein inference: a review. *Briefings in Bioinformatics*. 2012; doi: 10.1093/bib/bbs004.

79. Toby TK, Fornelli L, Kelleher NL. Progress in Top-Down Proteomics and the Analysis of Proteoforms. *Annual Rev Anal Chem*. 2016; doi: 10.1146/annurev-anchem-071015-041550.

80. Melani RD, Nogueira FCS, Domont GB. It is time for top-down venomics. *Journal of Venomous Animals and Toxins including Tropical Diseases*. 2017; doi: 10.1186/s40409-017-0135-6.

81. Hempel B-F, Damm M, Mrinalini, Göçmen B, Karış M, Nalbantsoy A, et al.. Extended Snake Venomics by Top-Down In-Source Decay: Investigating the Newly Discovered Anatolian Meadow Viper Subspecies, *Vipera anatolica senliki*. *J Proteome Res*. 2020; doi: 10.1021/acs.jproteome.9b00869.

82. Donnelly DP, Rawlins CM, DeHart CJ, Fornelli L, Schachner LF, Lin Z, et al.. Best practices and benchmarks for intact protein analysis for top-down mass spectrometry. *Nat Methods*. 2019; doi: 10.1038/s41592-019-0457-0.

83. Ghezellou P, Garikapati V, Kazemi SM, Strupat K, Ghassempour A, Spengler B. A perspective view of top-down proteomics in snake venom research. *Rapid Communications in Mass Spectrometry*. 2019; doi: 10.1002/rcm.8255.

84. Damm M, Hempel B-F, Süssmuth RD. Old World Vipers—A Review about Snake Venom Proteomics of Viperinae and Their Variations. *Toxins*. Multidisciplinary Digital Publishing Institute; 2021; doi: 10.3390/toxins13060427.

85. Vaudel M, Burkhart JM, Zahedi RP, Oveland E, Berven FS, Sickmann A, et al.. PeptideShaker enables reanalysis of MS-derived proteomics data sets. *Nat Biotechnol*. 2015; doi: 10.1038/nbt.3109.

86. Chen C, Hou J, Tanner JJ, Cheng J. Bioinformatics Methods for Mass Spectrometry-Based Proteomics Data Analysis. *International Journal of Molecular Sciences*. Multidisciplinary Digital Publishing Institute; 2020; doi: 10.3390/ijms21082873.

87. Hus KK, Marczak Ł, Petrilla V, Petrillová M, Legáth J, Bocian A. Different Research Approaches in Unraveling the Venom Proteome of *Naja ashei*. *Biomolecules*. Multidisciplinary Digital Publishing Institute; 2020; doi: 10.3390/biom10091282.

88. Yang H, Chi H, Zeng W-F, Zhou W-J, He S-M. pNovo 3: precise *de novo* peptide sequencing using a learning-to-rank framework. *Bioinformatics*. 2019; doi: 10.1093/bioinformatics/btz366.

89. Brahma RK, McCleary RJR, Kini RM, Doley R. Venom gland transcriptomics for identifying, cataloging, and characterizing venom proteins in snakes. *Toxicon*. 2015; doi: 10.1016/j.toxicon.2014.10.022.

90. Petras D, Hempel B-F, Göçmen B, Karis M, Whiteley G, Wagstaff SC, et al.. Intact protein mass spectrometry reveals intraspecies variations in venom composition of a local population of *Vipera kaznakovi* in Northeastern Turkey. *Journal of Proteomics*. 2019; doi: 10.1016/j.jprot.2019.02.004.

91. Catherman AD, Skinner OS, Kelleher NL. Top Down proteomics: Facts and perspectives. *Biochemical and Biophysical Research Communications*. 2014; doi: 10.1016/j.bbrc.2014.02.041.

92. Wang CR, Bubner ER, Jovcevski B, Mittal P, Pukala TL. Interrogating the higher order structures of snake venom proteins using an integrated mass spectrometric approach. *Journal of Proteomics*. 2020; doi: 10.1016/j.jprot.2020.103680.

93. Melani RD, Skinner OS, Fornelli L, Domont GB, Compton PD, Kelleher NL. Mapping Proteoforms and Protein Complexes From King Cobra Venom Using Both Denaturing and Native Top-down Proteomics *. *Molecular & Cellular Proteomics*. Elsevier; 2016; doi: 10.1074/mcp.M115.056523.

94. von Reumont BM. Studying Smaller and Neglected Organisms in Modern Evolutionary Venomics Implementing RNASeq (Transcriptomics)—A Critical Guide. *Toxins*. 2018; doi: 10.3390/toxins10070292.

95. Smith JJ, Undheim EAB. True Lies: Using Proteomics to Assess the Accuracy of Transcriptome-Based Venomics in Centipedes Uncovers False Positives and Reveals Startling Intraspecific Variation in *Scolopendra subspinipes*. *Toxins*. 2018; doi: 10.3390/toxins10030096.

96. Schuierer S, Carbone W, Knehr J, Petitjean V, Fernandez A, Sultan M, et al.. A comprehensive assessment of RNA-seq protocols for degraded and low-quantity samples. *BMC Genomics*. 2017; doi: 10.1186/s12864-017-3827-y.

97. Earl D, Bradnam K, St John J, Darling A, Lin D, Fass J, et al.. Assemblathon 1: a competitive assessment of de novo short read assembly methods. *Genome Research*. 2011; doi: 10.1101/gr.126599.111.

98. Hara Y, Tatsumi K, Yoshida M, Kajikawa E, Kiyonari H, Kuraku S. Optimizing and benchmarking *de novo* transcriptome sequencing: from library preparation to assembly evaluation. *BMC Genomics*. 2015; doi: 10.1186/s12864-015-2007-1.

99. Conesa A, Madrigal P, Tarazona S, Gomez-Cabrero D, Cervera A, McPherson A, et al.. A survey of best practices for RNA-seq data analysis. *Genome Biology*. 2016; doi: 10.1186/s13059-016-0881-8.

100. Holding ML, Margres MJ, Mason AJ, Parkinson CL, Rokyta DR. Evaluating the Performance of *De Novo* Assembly Methods for Venom-Gland Transcriptomics. *Toxins*. 2018; doi: 10.3390/toxins10060249.

101. Hölzer M, Marz M. De novo transcriptome assembly: A comprehensive cross-species comparison of short-read RNA-Seq assemblers. *GigaScience*. 2019; doi: 10.1093/gigascience/giz039.

102. Schurch NJ, Schofield P, Gierliński M, Cole C, Sherstnev A, Singh V, et al.. How many biological replicates are needed in an RNA-seq experiment and which differential expression tool should you use? *RNA*. 2016; doi: 10.1261/rna.053959.115.

103. Van den Berge K, Hembach KM, Soneson C, Tiberi S, Clement L, Love MI, et al.. RNA Sequencing Data: Hitchhiker's Guide to Expression Analysis. *Annual Review of Biomedical Data Science*. 2019; doi: 10.1146/annurev-biodatasci-072018-021255.

104. Steijger T, Abril JF, Engström PG, Kokocinski F, RGASP Consortium, Hubbard TJ, et al.. Assessment of transcript reconstruction methods for RNA-seq. *Nature Methods*. 2013; doi: 10.1038/nmeth.2714.

105. Venturini L, Caim S, Kaithakottil GG, Mapleson DL, Swarbreck D. Leveraging multiple transcriptome assembly methods for improved gene structure annotation. *GigaScience*. 2018; doi: 10.1093/gigascience/giy093.

106. Cerveau N, Jackson DJ. Combining independent *de novo* assemblies optimizes the coding transcriptome for nonconventional model eukaryotic organisms. *BMC Bioinformatics*. 2016; doi: 10.1186/s12859-016-1406-x.

107. MacManes MD. The Oyster River Protocol: a multi-assembler and kmer approach for *de novo* transcriptome assembly. *PeerJ*. PeerJ Inc.; 2018; doi: 10.7717/peerj.5428.

108. Rivera-Vicéns RE, Escudero CG, Conci N, Eitel M, Wörheide G. TransPi – a comprehensive TRanscriptome ANalysiS PIpeline for de novo transcriptome assembly. 2021 Feb.

109. Ramberg S, Høyheim B, Østbye T-KK, Andreassen R. A de novo Full-Length mRNA Transcriptome Generated From Hybrid-Corrected PacBio Long-Reads Improves the Transcript Annotation and Identifies Thousands of Novel Splice Variants in Atlantic Salmon. *Frontiers in Genetics*. 2021; doi: 10.3389/fgene.2021.656334.

110. Post Y, Puschhof J, Beumer J, Kerkkamp HM, de Bakker MAG, Slagboom J, et al.. Snake Venom Gland Organoids. *Cell*. 2020; doi: 10.1016/j.cell.2019.11.038.

111. Surm JM, Moran Y. Insights into how development and life-history dynamics shape the evolution of venom. *EvoDevo*. 2021; doi: 10.1186/s13227-020-00171-w.

112. García-Castro H, Kenny NJ, Iglesias M, Álvarez-Campos P, Mason V, Elek A, et al.. ACME dissociation: a versatile cell fixation-dissociation method for single-cell transcriptomics. *Genome Biol*. 2021; doi: 10.1186/s13059-021-02302-5.

113. Levy S, Elek A, Grau-Bové X, Menéndez-Bravo S, Iglesias M, Tanay A, et al.. A stony coral cell atlas illuminates the molecular and cellular basis of coral symbiosis, calcification, and immunity. *Cell*. 2021; doi: 10.1016/j.cell.2021.04.005.

114. Sebé-Pedrós A, Saudemont B, Chomsky E, Plessier F, Mailhé M-P, Renno J, et al.. Cnidarian Cell Type Diversity and Regulation Revealed by Whole-Organism Single-Cell RNA-Seq. *Cell*. Elsevier; 2018; doi: 10.1016/j.cell.2018.05.019.

115. Siebert S, Farrell JA, Cazet JF, Abeykoon Y, Primack AS, Schnitzler CE, et al.. Stem cell differentiation trajectories in *Hydra* resolved at single-cell resolution. *Science*. American Association for the Advancement of Science; 2019; doi: 10.1126/science.aav9314.

116. Yan F, Powell DR, Curtis DJ, Wong NC. From reads to insight: a hitchhiker's guide to ATAC-seq data analysis. *Genome Biol*. 2020; doi: 10.1186/s13059-020-1929-3.

117. Buenrostro JD, Giresi PG, Zaba LC, Chang HY, Greenleaf WJ. Transposition of native chromatin for fast and sensitive epigenomic profiling of open chromatin, DNA-binding proteins and nucleosome position. *Nat Methods*. 2013; doi: 10.1038/nmeth.2688.

118. Undheim EAB, Hamilton BR, Kurniawan ND, Bowlay G, Cribb BW, Merritt DJ, et al.. Production and packaging of a biological arsenal: evolution of centipede venoms under morphological constraint. *Proceedings of the National Academy of Sciences of the United States of America*. 2015; doi: 10.1073/pnas.1424068112.

119. Wurmbach H. Die Gewebe. *Lehrbuch der Zoologie - Zoologie und Ökologie*. Gustav Fischer Verlag Stuttgart; p. 72–136.

120. Müller CHG, Rosenberg J, Hilken G. Ultrastructure, functional morphology and evolution of recto-canal epidermal glands in Myriapoda. *Arthropod Structure & Development*. 2014; doi: 10.1016/j.asd.2013.08.001.

121. Farkaš R. Apocrine secretion: New insights into an old phenomenon. *Biochimica et Biophysica Acta (BBA) - General Subjects*. 2015; doi: 10.1016/j.bbagen.2015.05.003.

122. Ritman EL. Current Status of Developments and Applications of Micro-CT. *Annu Rev Biomed Eng*. 2011; doi: 10.1146/annurev-bioeng-071910-124717.

123. Gutiérrez Y, Ott D, Töpperwien M, Salditt T, Scherber C. X-ray computed tomography and its potential in ecological research: A review of studies and optimization of specimen preparation. *Ecology and Evolution*. 2018; doi: 10.1002/ece3.4149.

124. Hunter L, Dewanckele J. Evolution of Micro-CT: Moving from 3D to 4D. *Micros Today*. 2021; doi: 10.1017/S1551929521000651.

125. Arbuckle K. From molecules to macroevolution: Venom as a model system for evolutionary biology across levels of life. *Toxicon: X*. 2020; doi: 10.1016/j.toxcx.2020.100034.

126. Robinson SD, Mueller A, Clayton D, Starobova H, Hamilton BR, Payne RJ, et al.. A comprehensive portrait of the venom of the giant red bull ant, *Myrmecia gulosa*, reveals a hyperdiverse hymenopteran toxin gene family. *Science advances*. 2018; doi: 10.1126/sciadv.aau4640.

127. Walker, Robinson, Undheim, Jin, Han, Fry, et al.. Missiles of Mass Disruption: Composition and Glandular Origin of Venom Used as a Projectile Defensive Weapon by the Assassin Bug *Platymeris rhadamanthus*. *Toxins*. 2019; doi: 10.1074/mcp.M111.013987.

128. Walker AA, Mayhew ML, Jin J, Herzig V, Undheim EAB, Sombke A, et al.. The assassin bug *Pristhesancus plagipennis* produces two distinct venoms in separate gland lumens. *Nature Communications*. 2018; doi: 10.1038/s41467-018-03091-5.

129. Arvidson R, Kaiser M, Lee SS, Urenda J-P, Dail C, Mohammed H, et al.. Parasitoid Jewel Wasp Mounts Multipronged Neurochemical Attack to Hijack a Host Brain. *Molecular & Cellular Proteomics*. 2019; doi: 10.1074/mcp.RA118.000908.

130. Escalante T, Shannon J, Moura-da-Silva AM, María Gutiérrez J, Fox JW. Novel insights into capillary vessel basement membrane damage by snake venom hemorrhagic metalloproteinases: A biochemical and immunohistochemical study. *Archives of Biochemistry and Biophysics*. 2006; doi: 10.1016/j.abb.2006.09.018.

131. Baldo C, Ferreira MJ, Lopes DS, Izidoro LFM, Gomes AO, Ferro E a. V, et al.. Action of neuwiedase, a metalloproteinase isolated from *Bothrops neuwiedi* venom, on skeletal muscle: an ultrastructural and immunocytochemistry study. *J Venom Anim Toxins incl Trop Dis*. Centro de Estudos de Venenos e Animais Peçonhentos (CEVAP/UNESP); 2010; doi: 10.1590/S1678-91992010000300013.

132. Richter S, Helm C, Meunier FA, Hering L, Campbell LI, Drukewitz SH, et al.. Comparative analyses of glycerotoxin expression unveil a novel structural organization of the bloodworm venom system. *BMC Evol Biol*. 2017; doi: 10.1186/s12862-017-0904-4.

133. Lachumanan R, Armugam A, Durairaj P, Gopalakrishnakone P, Tan CH, Jeyaseelan K. In Situ Hybridization and Immunohistochemical Analysis of the Expression of Cardiotoxin and Neurotoxin Genes in *Naja naja sputatrix*. *J Histochem Cytochem*. 1999; doi: 10.1177/002215549904700414.

134. Han J, Permentier H, Bischoff R, Groothuis G, Casini A, Horvatovich P. Imaging of protein distribution in tissues using mass spectrometry: An interdisciplinary challenge. *TrAC Trends in Analytical Chemistry*. 2019; doi: 10.1016/j.trac.2018.12.016.

135. Madio B, Peigneur S, Chin YKY, Hamilton BR, Henriques ST, Smith JJ, et al.. PHAB toxins: a unique family of predatory sea anemone toxins evolving via intra-gene concerted evolution defines a new peptide fold. *Cell Mol Life Sci*. 2018; doi: 10.1007/s00018-018-2897-6.

136. Hamilton BR, Marshall DL, Casewell NR, Harrison RA, Blanksby SJ, Undheim EAB. Mapping Enzyme Activity on Tissue by Functional Mass Spectrometry Imaging. *Angewandte Chemie International Edition*. 2020; doi: 10.1002/anie.201911390.

137. Ghezellou P, Heiles S, Kadesch P, Ghassempour A, Spengler B. Venom Gland Mass Spectrometry Imaging of Saw-Scaled Viper, *Echis carinatus sochureki*, at High Lateral Resolution. *Journal of the American Society for Mass Spectrometry*. American Chemical Society; 2021; doi: 10.1021/jasms.1c00042.

138. Spraker JE, Luu GT, Sanchez LM. Imaging mass spectrometry for natural products discovery: a review of ionization methods. *Nat Prod Rep*. The Royal Society of Chemistry; 2020; doi: 10.1039/C9NP00038K.

139. Ståhl PL, Salmén F, Vickovic S, Lundmark A, Navarro JF, Magnusson J, et al.. Visualization and analysis of gene expression in tissue sections by spatial transcriptomics. *Science*. American Association for the Advancement of Science; 2016; doi: 10.1126/science.aaf2403.

140. Giacomello S, Salmén F, Terebieniec BK, Vickovic S, Navarro JF, Alexeyenko A, et al.. Spatially resolved transcriptome profiling in model plant species. *Nature Plants*. 2017; doi: 10.1038/nplants.2017.61.

141. Mantri M, Scuderi GJ, Abedini-Nassab R, Wang MFZ, McKellar D, Shi H, et al.. Spatiotemporal single-cell RNA sequencing of developing chicken hearts identifies interplay between cellular differentiation and morphogenesis. *Nat Commun*. 2021; doi: 10.1038/s41467-021-21892-z.

142. Giacomello S. A new era for plant science: spatial single-cell transcriptomics. *Current Opinion in Plant Biology*. 2021; doi: 10.1016/j.pbi.2021.102041.

143. Drukewitz SH, von Reumont BM. The Significance of Comparative Genomics in Modern Evolutionary Venomics. *Front Ecol Evol*. 2019; doi: 10.3389/fevo.2019.00163.

144. Lewin HA, Robinson GE, Kress WJ, Baker WJ, Coddington J, Crandall KA, et al.. Earth BioGenome Project: Sequencing life for the future of life. *Proc Natl Acad Sci USA*. 2018; doi: 10.1073/pnas.1720115115.

145. Rhie A, McCarthy SA, Fedrigo O, Damas J, Formenti G, Koren S, et al.. Towards complete and error-free genome assemblies of all vertebrate species. *Nature*. 2021; doi: 10.1038/s41586-021-03451-0.

146. Yin W, Wang Z-J, Li Q-Y, Lian J-M, Zhou Y, Lu B-Z, et al.. Evolutionary trajectories of snake genes and genomes revealed by comparative analyses of five-pacer viper. *Nature Communications*. 2016; doi: 10.1038/ncomms13107.

147. Gendreau KL, Haney RA, Schwager EE, Wierschin T, Stanke M, Richards S, et al.. House spider genome uncovers evolutionary shifts in the diversity and expression of black widow venom proteins associated with extreme toxicity. *BMC Genomics*. 2017; doi: 10.1186/s12864-017-3551-7.

148. Shibata H, Chijiwa T, Oda-Ueda N, Nakamura H, Yamaguchi K, Hattori S, et al.. The habu genome reveals accelerated evolution of venom protein genes. *Scientific Reports*. 2018; doi: 10.1038/s41598-018-28749-4.

149. Casewell NR, Petras D, Card DC, Suranse V, Mychajliw AM, Richards D, et al.. *Solenodon* genome reveals convergent evolution of venom in eulipotyphlan mammals. *Proc Natl Acad Sci USA*. 2019; doi: 10.1073/pnas.1906117116.

150. Suryamohan K, Krishnankutty SP, Guillory J, Jevit M, Schröder MS, Wu M, et al.. The Indian cobra reference genome and transcriptome enables comprehensive identification of venom toxins. *Nat Genet*. 2020; doi: 10.1038/s41588-019-0559-8.

151. Almeida DD, Viala VL, Nachtigall PG, Broe M, Gibbs HL, Serrano SM de T, et al.. Tracking the recruitment and evolution of snake toxins using the evolutionary context provided by the *Bothrops jararaca* genome. *PNAS*. National Academy of Sciences; 2021; doi: 10.1073/pnas.2015159118.

152. Sheffer MM, Hoppe A, Krehenwinkel H, Uhl G, Kuss AW, Jensen L, et al.. Chromosome-level reference genome of the European wasp spider *Argiope bruennichi*: a resource for studies on range expansion and evolutionary adaptation. *GigaScience*. 2021; doi: 10.1093/gigascience/giaa148.

153. Pardos-Blas JR, Irisarri I, Abalde S, Afonso CML, Tenorio MJ, Zardoya R. The genome of the venomous snail *Lautoconus ventricosus* sheds light on the origin of conotoxin diversity. *GigaScience*. 2021; doi: 10.1093/gigascience/giab037.

154. Drukewitz SH, Bokelmann L, Undheim EAB, von Reumont BM. Toxins from scratch? Diverse, multimodal gene origins in the predatory robber fly *Dasypogon diadema* indicate a dynamic venom evolution in dipteran insects. *GigaScience*. 2019; doi: 10.1093/gigascience/giz081.

155. Salzberg SL. Next-generation genome annotation: we still struggle to get it right. *Genome Biology*. 2019; doi: 10.1186/s13059-019-1715-2.

156. Koludarov I, Jackson TN, Suranse V, Pozzi A, Sunagar K, Mikheyev AS. Reconstructing the evolutionary history of a functionally diverse gene family reveals complexity at the genetic origins of novelty. Molecular Biology; 2019 Mar.

157. Jackson TNW, Koludarov I. How the Toxin got its Toxicity. *Frontiers in Pharmacology*. 2020; doi: 10.3389/fphar.2020.574925.

158. Malhotra A, Creer S, Harris JB, Thorpe RS. The importance of being genomic: Non-coding and coding sequences suggest different models of toxin multi-gene family evolution. *Toxicon*. 2015; doi: 10.1016/j.toxicon.2015.08.009.

159. Kini RM. Accelerated evolution of toxin genes: Exonization and intronization in snake venom disintegrin/metalloprotease genes. *Toxicon*. 2018; doi: 10.1016/j.toxicon.2018.04.005.

160. Bergthorsson U, Andersson DI, Roth JR. Ohno's dilemma: Evolution of new genes under continuous selection. *Proceedings of the National Academy of Sciences*. 2007; doi: 10.1073/pnas.0707158104.

161. Patthy L. Protein Evolution. John Wiley & Sons;

162. Innan H, Kondrashov F. The evolution of gene duplications: classifying and distinguishing between models. *Nat Rev Genet*. 2010; doi: 10.1038/nrg2689.

163. Espinosa-Cantú A, Ascencio D, Barona-Gómez F, DeLuna A. Gene duplication and the evolution of moonlighting proteins. *Frontiers in Genetics*. 2015; doi: 10.3389/fgene.2015.00227.

164. Veltri D, Wight MM, Crouch JA. SimpleSynteny: a web-based tool for visualization of microsynteny across multiple species. *Nucleic Acids Res*. 2016; doi: 10.1093/nar/gkw330.

165. Haug-Baltzell A, Stephens SA, Davey S, Scheidegger CE, Lyons E. SynMap2 and SynMap3D: web-based whole-genome synteny browsers. Hancock J, editor. *Bioinformatics*. 2017; doi: 10.1093/bioinformatics/btx144.

166. Ankenbrand MJ, Hohlfeld S, Hackl T, Förster F. AliTV—interactive visualization of whole genome comparisons. *PeerJ Comput Sci*. PeerJ Inc.; 2017; doi: 10.7717/peerj-cs.116.

167. Hilbrant M, Damen WGM, McGregor AP. Evolutionary crossroads in developmental biology: the spider *Parasteatoda tepidariorum*. *Development*. 2012; doi: 10.1242/dev.078204.

168. Oda H, Akiyama-Oda Y. The common house spider *Parasteatoda tepidariorum*. *EvoDevo*. 2020; doi: 10.1186/s13227-020-00152-z.

169. Li M, Au LYC, Douglah D, Chong A, White BJ, Ferree PM, et al.. Generation of heritable germline mutations in the jewel wasp *Nasonia vitripennis* using CRISPR/Cas9. *Sci Rep*. 2017; doi: 10.1038/s41598-017-00990-3.

170. Hu XF, Zhang B, Liao CH, Zeng ZJ. High-Efficiency CRISPR/Cas9-Mediated Gene Editing in Honeybee (*Apis mellifera*) Embryos. *G3: Genes, Genomes, Genetics*. G3: Genes, Genomes, Genetics; 2019; doi: 10.1534/g3.119.400130.

171. Chiu Y-K, Hsu J-C, Chang T, Huang Y-C, Wang J. Mutagenesis mediated by CRISPR/Cas9 in the red imported fire ant, *Solenopsis invicta*. *Insect Soc*. 2020; doi: 10.1007/s00040-020-00755-8.

172. Karabulut A, He S, Chen C-Y, McKinney SA, Gibson MC. Electroporation of short hairpin RNAs for rapid and efficient gene knockdown in the starlet sea anemone, *Nematostella vectensis*. *Developmental Biology*. 2019; doi: 10.1016/j.ydbio.2019.01.005.

173. Layden MJ, Rentzsch F, Röttinger E. The rise of the starlet sea anemone *Nematostella vectensis* as a model system to investigate development and regeneration. *WIREs Developmental Biology*. 2016; doi: 10.1002/wdev.222.

174. Zancolli G, Casewell NR. Venom Systems as Models for Studying the Origin and Regulation of Evolutionary Novelties. Kelley J, editor. *Molecular Biology and Evolution*. 2020; doi: 10.1093/molbev/msaa133.

175. Columbus-Shenkar YY, Sachkova MY, Macrander J, Fridrich A, Modepalli V, Reitzel AM, et al.. Dynamics of venom composition across a complex life cycle. *Elife*. 2018; doi: 10.7554/eLife.35014.

176. Moran Y, Genikhovich G, Gordon D, Wienkoop S, Zenkert C, Oezbek S, et al.. Neurotoxin localization to ectodermal gland cells uncovers an alternative mechanism of venom delivery in sea anemones. *Proceedings of the Royal Society B: Biological Sciences*. 2012; doi: 10.1098/rspb.2011.1731.

177. Sunagar K, Columbus-Shenkar YY, Fridrich A, Gutkovich N, Aharoni R, Moran Y. Cell type-specific expression profiling unravels the development and evolution of stinging cells in sea anemone. *BMC Biol*. 2018; doi: 10.1186/s12915-018-0578-4.

178. Herzig V, King GF, Undheim EAB. Can we resolve the taxonomic bias in spider venom research? *Toxicon: X*. 2019; doi: 10.1016/j.toxcx.2018.100005.

179. Lüddecke T, Vilcinskas A, Lemke S. Phylogeny-Guided Selection of Priority Groups for Venom Bioprospecting: Harvesting Toxin Sequences in Tarantulas as a Case Study. *Toxins*. Multidisciplinary Digital Publishing Institute; 2019; doi: 10.3390/toxins11090488.

180. Jin A-H, Muttenthaler M, Dutertre S, Himaya SWA, Kaas Q, Craik DJ, et al.. Conotoxins: Chemistry and Biology. *Chemical Reviews*. American Chemical Society; 2019; doi: 10.1021/acs.chemrev.9b00207.

181. Wang Y-M, Tsai I-H, Chen J-M, Cheng A-C, Khoo K-H. Correlation between the Glycan Variations and Defibrinogenating Activities of Acutobin and Its Recombinant Glycoforms. *PLOS ONE*. Public Library of Science; 2014; doi: 10.1371/journal.pone.0100354.

182. Luna-Ramirez K, Csoti A, McArthur JR, Chin YKY, Anangi R, Najera R del C, et al.. Structural basis of the potency and selectivity of Urotoxin, a potent Kv1 blocker from scorpion venom. *Biochemical Pharmacology*. 2020; doi: 10.1016/j.bcp.2019.113782.

183. Lee H-K, Zhang L, Smith MD, Walewska A, Vellore NA, Baron R, et al.. A marine analgesic peptide, Contulakin-G, and neurotensin are distinct agonists for neurotensin receptors: uncovering structural determinants of desensitization properties. *Frontiers in Pharmacology*. 2015; doi: 10.3389/fphar.2015.00011.

184. Saikia C, Ben-Nissan G, Reuveny E, Karbat I. Chapter Seven - Production of recombinant venom peptides as tools for ion channel research. In: Minor DL, Colecraft HM, editors. *Methods in Enzymology*. Academic Press;

185. Turchetto J, Sequeira AF, Ramond L, Peysson F, Brás JLA, Saez NJ, et al.. High-throughput expression of animal venom toxins in *Escherichia coli* to generate a large library of oxidized disulphide-reticulated peptides for drug discovery. *Microbial Cell Factories*. 2017; doi: 10.1186/s12934-016-0617-1.

186. Derman AI, Prinz WA, Belin D, Beckwith J. Mutations that Allow Disulfide Bond Formation in the Cytoplasm of *Escherichia coli*. *Science*. American Association for the Advancement of Science; 1993; doi: 10.1126/science.8259521.

187. Bessette PH, Aslund F, Beckwith J, Georgiou G. Efficient folding of proteins with multiple disulfide bonds in the *Escherichia coli* cytoplasm. *Proceedings of the National Academy of Sciences*. 1999; doi: 10.1073/pnas.96.24.13703.

188. de Marco A. Strategies for successful recombinant expression of disulfide bond-dependent proteins in *Escherichia coli*. *Microbial Cell Factories*. 2009; doi: 10.1186/1475-2859-8-26.

189. Hatahet F, Nguyen VD, Salo KE, Ruddock LW. Disruption of reducing pathways is not essential for efficient disulfide bond formation in the cytoplasm of *E. coli*. *Microbial Cell Factories*. 2010; doi: 10.1186/1475-2859-9-67.

190. Klint JK, Senff S, Saez NJ, Seshadri R, Lau HY, Bende NS, et al.. Production of Recombinant Disulfide-Rich Venom Peptides for Structural and Functional Analysis via Expression in the

Periplasm of *E. coli*. *PLOS ONE*. Public Library of Science; 2013; doi: 10.1371/journal.pone.0063865.

191. Bertelsen AB, Hackney CM, Bayer CN, Kjelgaard LD, Rennig M, Christensen B, et al.. DisCoTune: versatile auxiliary plasmids for the production of disulphide-containing proteins and peptides in the *E. coli* T7 system. *Microbial Biotechnology*. 2021; doi: 10.1111/1751-7915.13895.

192. Nielsen LD, Foged MM, Albert A, Bertelsen AB, Søltoft CL, Robinson SD, et al.. The three-dimensional structure of an H-superfamily conotoxin reveals a granulin fold arising from a common ICK cysteine framework. *Journal of Biological Chemistry*. Elsevier; 2019; doi: 10.1074/jbc.RA119.007491.

193. Nozach H, Fruchart-Gaillard C, Fenaille F, Beau F, Ramos OHP, Douzi B, et al.. High throughput screening identifies disulfide isomerase DsbC as a very efficient partner for recombinant expression of small disulfide-rich proteins in *E. coli*. *Microbial Cell Factories*. 2013; doi: 10.1186/1475-2859-12-37.

194. Sequeira AF, Turchetto J, Saez NJ, Peysson F, Ramond L, Duhoo Y, et al.. Gene design, fusion technology and TEV cleavage conditions influence the purification of oxidized disulphide-rich venom peptides in *Escherichia coli*. *Microbial Cell Factories*. 2017; doi: 10.1186/s12934-016-0618-0.

195. Correnti CE, Gewe MM, Mehlin C, Bandaranayake AD, Johnsen WA, Rupert PB, et al.. Screening, large-scale production and structure-based classification of cystine-dense peptides. *Nat Struct Mol Biol*. 2018; doi: 10.1038/s41594-018-0033-9.

196. Crook ZR, Sevilla GP, Friend D, Brusniak M-Y, Bandaranayake AD, Clarke M, et al.. Mammalian display screening of diverse cystine-dense peptides for difficult to drug targets. *Nat Commun*. 2017; doi: 10.1038/s41467-017-02098-8.

197. Wang Y, Xu W, Kou X, Luo Y, Zhang Y, Ma B, et al.. Establishment and optimization of a wheat germ cell-free protein synthesis system and its application in venom kallikrein. *Protein Expression and Purification*. 2012; doi: 10.1016/j.pep.2012.05.006.

198. Vlasak R, Kreil G. Nucleotide sequence of cloned cDNAs coding for preprosecapin, a major product of queen-bee venom glands. *European Journal of Biochemistry*. 1984; doi: 10.1111/j.1432-1033.1984.tb08549.x.

199. Pennington MW, Czerwinski A, Norton RS. Peptide therapeutics from venom: Current status and potential. *Bioorganic & Medicinal Chemistry*. 2018; doi: 10.1016/j.bmc.2017.09.029.

200. Robinson SD, Undheim EAB, Ueberheide B, King GF. Venom peptides as therapeutics: advances, challenges and the future of venom-peptide discovery. *Expert Review of Proteomics*. 2017; doi: 10.1080/14789450.2017.1377613.

201. Theakston RDG, Reid HA. Development of simple standard assay procedures for the characterization of snake venoms. 61:949–561983;

202. Giacomotto J, Ségalat L. High-throughput screening and small animal models, where are we?: High-throughput screening and small animal models. *British Journal of Pharmacology*. 2010; doi: 10.1111/j.1476-5381.2010.00725.x.

203. Vetter I, Hodgson WC, Adams DJ, McIntyre P. CHAPTER 4:Venoms-Based Drug Discovery: Bioassays, Electrophysiology, High-Throughput Screens and Target Identification. *Venoms to Drugs*.

204. Herzig V, Cristofori-Armstrong B, Israel MR, Nixon SA, Vetter I, King GF. Animal toxins — Nature's evolutionary-refined toolkit for basic research and drug discovery. *Biochemical Pharmacology*. 2020; doi: 10.1016/j.bcp.2020.114096.

205. Gutiérrez JM, Vargas M, Segura Á, Herrera M, Villalta M, Solano G, et al.. In Vitro Tests for Assessing the Neutralizing Ability of Snake Antivenoms: Toward the 3Rs Principles. *Frontiers in Immunology*. 2021; doi: 10.3389/fimmu.2020.617429.

206. Mejia M, Heghinian MD, Busch A, Armishaw CJ, Marí F, Godenschwege TA. A novel approach for *in vivo* screening of toxins using the Drosophila Giant Fiber circuit. *Toxicon*. 2010; doi: 10.1016/j.toxicon.2010.08.005.

207. Freshney IR. Culture of Animal Cells: A Manual of Basic Technique and Specialized ApplicationsFreshney. John Wiley & Sons, Inc.;

208. Mathie A, Veale EL, Holden RG. Heterologous Expression of Ion Channels in Mammalian Cell Lines. In: Dallas M, Bell D, editors. *Patch Clamp Electrophysiology: Methods and Protocols*. New York, NY: Springer US;

209. Penner R. A Practical Guide to Patch Clamping. In: Sakmann B, Neher E, editors. *Single-Channel Recording*. Boston, MA: Springer US;

210. Schreibmayer W, Lester HA, Dascal N. Voltage clamping of *Xenopus laevis* oocytes utilizing agarose-cushion electrodes. *Pflugers Arch*. 1994; doi: 10.1007/BF00388310.

211. van Cann M, Kuzmenkov A, Isensee J, Andreev-Andrievskiy A, Peigneur S, Khusainov G, et al.. Scorpion toxin MeuNaTxα-1 sensitizes primary nociceptors by selective modulation of voltage-gated sodium channels. *The FEBS Journal*. 2021; doi: 10.1111/febs.15593.

212. Broichhagen J, Frank JA, Trauner D. A Roadmap to Success in Photopharmacology. *Acc Chem Res*. American Chemical Society; 2015; doi: 10.1021/acs.accounts.5b00129.

213. Russell WMS, Burch RL, Universities Federation for Animal Welfare. The Principles of humane experimental technique. Wheathampstead: Universities Federation for Animal Welfare;

214. Gordon D, Chen R, Chung S-H. Computational Methods of Studying the Binding of Toxins From Venomous Animals to Biological Ion Channels: Theory and Applications. *Physiological Reviews*. American Physiological Society; 2013; doi: 10.1152/physrev.00035.2012.

215. Mouhat S, Jouirou B, Mosbah A, De Waard M, Sabatier J-M. Diversity of folds in animal toxins acting on ion channels. *Biochemical Journal*. 2004; doi: 10.1042/bj20031860.

216. Lavergne V, Alewood PF, Mobli M, King GF. CHAPTER 2:The Structural Universe of Disulfide-Rich Venom Peptides. *Venoms to Drugs*.

217. Callaway E. Revolutionary cryo-EM is taking over structural biology. *Nature*. 2020; doi: 10.1038/d41586-020-00341-9.

218. Baconguis I, Bohlen CJ, Goehring A, Julius D, Gouaux E. X-Ray Structure of Acid-Sensing Ion Channel 1–Snake Toxin Complex Reveals Open State of a Na+-Selective Channel. *Cell*. 2014; doi: 10.1016/j.cell.2014.01.011.

219. Shen H, Li Z, Jiang Y, Pan X, Wu J, Cristofori-Armstrong B, et al.. Structural basis for the modulation of voltage-gated sodium channels by animal toxins. *Science*. American Association for the Advancement of Science; 2018; doi: 10.1126/science.aau2596.

220. Clairfeuille T, Cloake A, Infield DT, Llongueras JP, Arthur CP, Li ZR, et al.. Structural basis of α-scorpion toxin action on Nav channels. *Science*. American Association for the Advancement of Science; 2019; doi: 10.1126/science.aav8573.

221. Maeda S, Xu J, N. Kadji FM, Clark MJ, Zhao J, Tsutsumi N, et al.. Structure and selectivity engineering of the M1 muscarinic receptor toxin complex. *Science*. American Association for the Advancement of Science; 2020; doi: 10.1126/science.aax2517.

222. Pagadala NS, Syed K, Tuszynski J. Software for molecular docking: a review. *Biophys Rev*. 2017; doi: 10.1007/s12551-016-0247-1.

223. Hollingsworth SA, Dror RO. Molecular Dynamics Simulation for All. *Neuron*. 2018; doi: 10.1016/j.neuron.2018.08.011.

224. Karbat I, Altman-Gueta H, Fine S, Szanto T, Hamer-Rogotner S, Dym O, et al.. Pore-modulating toxins exploit inherent slow inactivation to block K+ channels. *PNAS*. National Academy of Sciences; 2019; doi: 10.1073/pnas.1908903116.

225. Saikia C, Dym O, Altman-Gueta H, Gordon D, Reuveny E, Karbat I. A Molecular Lid Mechanism of K+ Channel Blocker Action Revealed by a Cone Peptide. *Journal of Molecular Biology*. 2021; doi: 10.1016/j.jmb.2021.166957.

226. Yi M, Tjong H, Zhou H-X. Spontaneous conformational change and toxin binding in α7 acetylcholine receptor: Insight into channel activation and inhibition. *PNAS*. National Academy of Sciences; 2008; doi: 10.1073/pnas.0710530105.

227. Jumper J, Evans R, Pritzel A, Green T, Figurnov M, Ronneberger O, et al.. Highly accurate protein structure prediction with AlphaFold. *Nature*. 2021; doi: 10.1038/s41586-021-03819-2.

228. Isensee J, van Cann M, Despang P, Araldi D, Moeller K, Petersen J, et al.. Depolarization induces nociceptor sensitization by CaV1.2-mediated PKA-II activation. *Journal of Cell Biology*. 2021; doi: 10.1083/jcb.202002083.

229. Wilson D, Daly NL. Venomics: A Mini-Review. *High-Throughput*. Multidisciplinary Digital Publishing Institute; 2018; doi: 10.3390/ht7030019.

230. Prashanth JR, Hasaballah N, Vetter I. Pharmacological screening technologies for venom peptide discovery. *Neuropharmacology*. 2017; doi: 10.1016/j.neuropharm.2017.03.038.

231. Clark GC, Casewell NR, Elliott CT, Harvey AL, Jamieson AG, Strong PN, et al.. Friends or Foes? Emerging Impacts of Biological Toxins. *Trends in Biochemical Sciences*. Elsevier Ltd; 2019; doi: 10.1016/j.tibs.2018.12.004.

232. Vetter I, Davis JL, Rash LD, Anangi R, Mobli M, Alewood PF, et al.. Venomics: a new paradigm for natural products-based drug discovery. *Amino acids*. 2011; doi: 10.1007/s00726-010-0516-4.

233. Bhaswati C. Animal Venoms have Potential to Treat Cancer. *Current Topics in Medicinal Chemistry*. 18:2555–662018;

234. Chan YS, Cheung RCF, Xia L, Wong JH, Ng TB, Chan WY. Snake venom toxins: toxicity and medicinal applications. *Appl Microbiol Biotechnol*. 2016; doi: 10.1007/s00253-016-7610-9.

235. Harvey AL. Toxins and drug discovery. *Toxicon*. 2014; doi: 10.1016/j.toxicon.2014.10.020.

236. Lewis RJ, Garcia ML. Therapeutic potential of venom peptides. *Nat Rev Drug Discov*. 2003; doi: 10.1038/nrd1197.

237. Trim CM, Byrne LJ, Trim SA. Chapter One - Utilisation of compounds from venoms in drug discovery. In: Witty DR, Cox B, editors. *Progress in Medicinal Chemistry*. Elsevier;

238. Cardoso FC, Hasan M, Zhao T, Lewis RJ. Toxins in pain. *Current Opinion in Supportive and Palliative Care*. 2018; doi: 10.1097/SPC.0000000000000335.

239. Geron M, Hazan A, Priel A. Animal Toxins Providing Insights into TRPV1 Activation Mechanism. *Toxins (Basel)*. 2017; doi: 10.3390/toxins9100326.

240. Andreev YA, Kozlov SA, Korolkova YV, Dyachenko IA, Bondarenko DA, Skobtsov DI, et al.. Polypeptide Modulators of TRPV1 Produce Analgesia without Hyperthermia. *Marine Drugs*. Multidisciplinary Digital Publishing Institute; 2013; doi: 10.3390/md11125100.

241. Koivisto A, Chapman H, Jalava N, Korjamo T, Saarnilehto M, Lindstedt K, et al.. TRPA1: A Transducer and Amplifier of Pain and Inflammation. *Basic & Clinical Pharmacology & Toxicology*. 2014; doi: 10.1111/bcpt.12138.

242. Escoubas P, Weille JRD, Lecoq A, Diochot S, Waldmann R, Champigny G, et al.. Isolation of a Tarantula Toxin Specific for a Class of Proton-gated Na+ Channels *. *Journal of Biological Chemistry*. Elsevier; 2000; doi: 10.1074/jbc.M003643200.

243. Diochot S, Baron A, Salinas M, Douguet D, Scarzello S, Dabert-Gay A-S, et al.. Black mamba venom peptides target acid-sensing ion channels to abolish pain. *Nature*. 2012; doi: 10.1038/nature11494.

244. Lee JYP, Saez NJ, Cristofori-Armstrong B, Anangi R, King GF, Smith MT, et al.. Inhibition of acid-sensing ion channels by diminazene and APETx2 evoke partial and highly variable antihyperalgesia in a rat model of inflammatory pain. *Br J Pharmacol*. 2018; doi: 10.1111/bph.14089.

245. Postic G, Gracy J, Perin C, Chiche L, Gelly J-C. KNOTTIN: the database of inhibitor cystine knot scaffold after 10 years, toward a systematic structure modeling. *Nucleic Acids Research*. 2018; doi: 10.1093/nar/gkx1084.

246. Moore SJ, Leung CL, Cochran JR. Knottins: disulfide-bonded therapeutic and diagnostic peptides. *Drug Discovery Today: Technologies*. Elsevier Ltd; 2012; doi: 10.1016/j.ddtec.2011.07.003.

247. McDowell GC, Pope JE. Intrathecal Ziconotide: Dosing and Administration Strategies in Patients With Refractory Chronic Pain. *Neuromodulation*. 2016; doi: 10.1111/ner.12392.

248. Brust A, Croker DE, Colless B, Ragnarsson L, Andersson Å, Jain K, et al.. Conopeptide-Derived κ-Opioid Agonists (Conorphins): Potent, Selective, and Metabolic Stable Dynorphin A Mimetics with Antinociceptive Properties. *J Med Chem*. American Chemical Society; 2016; doi: 10.1021/acs.jmedchem.5b00911.

249. Castro J, Harrington AM, Garcia-Caraballo S, Maddern J, Grundy L, Zhang J, et al.. α-Conotoxin Vc1.1 inhibits human dorsal root ganglion neuroexcitability and mouse colonic nociception via GABAB receptors. *Gut*. BMJ Publishing Group; 2017; doi: 10.1136/gutjnl-2015-310971.

250. Nasiripourdori A, Taly V, Grutter T, Taly A. From Toxins Targeting Ligand Gated Ion Channels to Therapeutic Molecules. *Toxins (Basel)*. 2011; doi: 10.3390/toxins3030260.

251. Nilius B, Szallasi A. Transient Receptor Potential Channels as Drug Targets: From the Science of Basic Research to the Art of Medicine. Sibley DR, editor. *Pharmacol Rev*. American Society for Pharmacology and Experimental Therapeutics; 2014; doi: 10.1124/pr.113.008268.

252. Heinen TE, da Veiga ABG. Arthropod venoms and cancer. *Toxicon*. 2011; doi: 10.1016/j.toxicon.2011.01.002.

253. Gajski G, Garaj-Vrhovac V. Melittin: A lytic peptide with anticancer properties. *Environmental Toxicology and Pharmacology*. 2013; doi: 10.1016/j.etap.2013.06.009.

254. Fernandez-Rojo MA, Deplazes E, Pineda SS, Brust A, Marth T, Wilhelm P, et al.. Gomesin peptides prevent proliferation and lead to the cell death of devil facial tumour disease cells. *Cell Death Discovery*. 2018; doi: 10.1038/s41420-018-0030-0.

255. Ikonomopoulou MP, Fernandez-Rojo MA, Pineda SS, Cabezas-Sainz P, Winnen B, Morales RAV, et al.. Gomesin inhibits melanoma growth by manipulating key signaling cascades that control cell death and proliferation. *Sci Rep*. 2018; doi: 10.1038/s41598-018-29826-4.

256. Moral-Sanz J, Fernandez-Rojo MA, Potriquet J, Mukhopadhyay P, Brust A, Wilhelm P, et al.. ERK and mTORC1 Inhibitors Enhance the Anti-Cancer Capacity of the Octpep-1 Venom-Derived Peptide in Melanoma BRAF(V600E) Mutations. *Toxins*. Multidisciplinary Digital Publishing Institute; 2021; doi: 10.3390/toxins13020146.

257. Panagides N, Jackson TNW, Ikonomopoulou MP, Arbuckle K, Pretzler R, Yang DC, et al.. How the Cobra Got Its Flesh-Eating Venom: Cytotoxicity as a Defensive Innovation and Its Co-Evolution with Hooding, Aposematic Marking, and Spitting. *Toxins*. Multidisciplinary Digital Publishing Institute; 2017; doi: 10.3390/toxins9030103.

258. Goldenberg J, Cipriani V, Jackson TNW, Arbuckle K, Debono J, Dashevsky D, et al.. Proteomic and functional variation within black snake venoms (Elapidae: *Pseudechis*). *Comparative Biochemistry and Physiology Part C: Toxicology & Pharmacology*. 2018; doi: 10.1016/j.cbpc.2018.01.001.

259. op den Brouw B, Coimbra FCP, Bourke LA, Huynh TM, Vlecken DHW, Ghezellou P, et al.. Extensive Variation in the Activities of Pseudocerastes and Eristicophis Viper Venoms Suggests Divergent Envenoming Strategies Are Used for Prey Capture. *Toxins*. Multidisciplinary Digital Publishing Institute; 2021; doi: 10.3390/toxins13020112.

260. Duffy C, Sorolla A, Wang E, Golden E, Woodward E, Davern K, et al.. Honeybee venom and melittin suppress growth factor receptor activation in HER2-enriched and triple-negative breast cancer. *npj Precis Onc*. 2020; doi: 10.1038/s41698-020-00129-0.

261. Li L, Huang J, Lin Y. Snake Venoms in Cancer Therapy: Past, Present and Future. *Toxins*. Multidisciplinary Digital Publishing Institute; 2018; doi: 10.3390/toxins10090346.

262. Gajski G, Domijan A-M, Žegura B, Štern A, Gerić M, Novak Jovanović I, et al.. Melittin induced cytogenetic damage, oxidative stress and changes in gene expression in human peripheral blood lymphocytes. *Toxicon*. 2016; doi: 10.1016/j.toxicon.2015.12.005.

263. Dabbagh Moghaddam F, Akbarzadeh I, Marzbankia E, Farid M, khaledi L, Reihani AH, et al.. Delivery of melittin-loaded niosomes for breast cancer treatment: an *in vitro* and *in vivo* evaluation of anti-cancer effect. *Cancer Nanotechnology*. 2021; doi: 10.1186/s12645-021-00085-9.

264. Jimenez R, Ikonomopoulou MP, Lopez JA, Miles JJ. Immune drug discovery from venoms. *Toxicon*. 2018; doi: 10.1016/j.toxicon.2017.11.006.

265. Minutti-Zanella C, Gil-Leyva EJ, Vergara I. Immunomodulatory properties of molecules from animal venoms. *Toxicon*. 2021; doi: 10.1016/j.toxicon.2020.12.018.

266. Ryan RYM, Seymour J, Loukas A, Lopez JA, Ikonomopoulou MP, Miles JJ. Immunological Responses to Envenomation. *Frontiers in Immunology*. 2021; doi: 10.3389/fimmu.2021.661082.

267. Freitas AP, Favoretto BC, Clissa PB, Sampaio SC, Faquim-Mauro EL. Crotoxin Isolated from Crotalus durissus terrificus Venom Modulates the Functional Activity of Dendritic Cells via Formyl Peptide Receptors. *Journal of Immunology Research*. Hindawi; 2018; doi: 10.1155/2018/7873257.

268. Almeida C de S, Andrade-Oliveira V, Câmara NOS, Jacysyn JF, Faquim-Mauro EL. Crotoxin from *Crotalus durissus terrificus* Is Able to Down-Modulate the Acute Intestinal Inflammation in Mice. *PLOS ONE*. Public Library of Science; 2015; doi: 10.1371/journal.pone.0121427.

269. Huan Y, Kong Q, Mou H, Yi H. Antimicrobial Peptides: Classification, Design, Application and Research Progress in Multiple Fields. *Frontiers in Microbiology*. 2020; doi: 10.3389/fmicb.2020.582779.

270. Datta S, Roy A. Antimicrobial Peptides as Potential Therapeutic Agents: A Review. *Int J Pept Res Ther*. 2021; doi: 10.1007/s10989-020-10110-x.

271. Gan BH, Gaynord J, Rowe SM, Deingruber T, Spring DR. The multifaceted nature of antimicrobial peptides: current synthetic chemistry approaches and future directions. *Chem Soc Rev*. The Royal Society of Chemistry; 2021; doi: 10.1039/D0CS00729C.

272. de Barros E, Gonçalves RM, Cardoso MH, Santos NC, Franco OL, Cândido ES. Snake Venom Cathelicidins as Natural Antimicrobial Peptides. *Frontiers in Pharmacology*. 2019; doi: 10.3389/fphar.2019.01415.

273. Manniello MD, Moretta A, Salvia R, Scieuzo C, Lucchetti D, Vogel H, et al.. Insect antimicrobial peptides: potential weapons to counteract the antibiotic resistance. *Cell Mol Life Sci*. 2021; doi: 10.1007/s00018-021-03784-z.

274. Moreau SJ. "It stings a bit but it cleans well": Venoms of Hymenoptera and their antimicrobial potential. *Journal of Insect Physiology*. 2013; doi: 10.1016/j.jinsphys.2012.10.005.

275. Mylonakis E, Podsiadlowski L, Muhammed M, Vilcinskas A. Diversity, evolution and medical applications of insect antimicrobial peptides. *Philosophical Transactions of the Royal Society B: Biological Sciences*. Royal Society; 2016; doi: 10.1098/rstb.2015.0290.

276. Zhou W, Qiu H, Guo Y, Guo W. Molecular Insights into Distinct Detection Properties of α-Hemolysin, MspA, CsgG, and Aerolysin Nanopore Sensors. *J Phys Chem B*. American Chemical Society; 2020; doi: 10.1021/acs.jpcb.9b10702.

277. Crnković A, Srnko M, Anderluh G. Biological Nanopores: Engineering on Demand. *Life*. Multidisciplinary Digital Publishing Institute; 2021; doi: 10.3390/life11010027.

278. Morton D, Mortezaei S, Yemenicioglu S, Isaacman MJ, Nova IC, Gundlach JH, et al.. Tailored polymeric membranes for Mycobacterium smegmatis porin A (MspA) based biosensors. *J Mater Chem B*. The Royal Society of Chemistry; 2015; doi: 10.1039/C5TB00383K.

279. Huang G, Voet A, Maglia G. FraC nanopores with adjustable diameter identify the mass of opposite-charge peptides with 44 dalton resolution. *Nat Commun*. 2019; doi: 10.1038/s41467-019-08761-6.

280. Carter J-M, Hussain S. Robust long-read native DNA sequencing using the ONT CsgG Nanopore system. Wellcome Open Research;

281. Wloka C, Mutter NL, Soskine M, Maglia G. Alpha-Helical Fragaceatoxin C Nanopore Engineered for Double-Stranded and Single-Stranded Nucleic Acid Analysis. *Angewandte Chemie International Edition*. 2016; doi: 10.1002/anie.201606742.

282. Zernia S, van der Heide NJ, Galenkamp NS, Gouridis G, Maglia G. Current Blockades of Proteins inside Nanopores for Real-Time Metabolome Analysis. *ACS Nano*. American Chemical Society; 2020; doi: 10.1021/acsnano.9b09434.

283. Lucas FLR, Versloot RCA, Yakovlieva L, Walvoort MTC, Maglia G. Protein identification by nanopore peptide profiling. *Nat Commun*. 2021; doi: 10.1038/s41467-021-26046-9.

284. Laszlo AH, Derrington IM, Ross BC, Brinkerhoff H, Adey A, Nova IC, et al.. Decoding long nanopore sequencing reads of natural DNA. *Nat Biotechnol*. 2014; doi: 10.1038/nbt.2950.

285. Schäfer RB, Liess M, Altenburger R, Filser J, Hollert H, Roß-Nickoll M, et al.. Future pesticide risk assessment: narrowing the gap between intention and reality. *Environmental Sciences Europe*. 2019; doi: 10.1186/s12302-019-0203-3.

286. Sharma A, Kumar V, Shahzad B, Tanveer M, Sidhu GPS, Handa N, et al.. Worldwide pesticide usage and its impacts on ecosystem. *SN Appl Sci*. 2019; doi: 10.1007/s42452-019-1485-1.

287. Zhu YC, Adamczyk J, Rinderer T, Yao J, Danka R, Luttrell R, et al.. Spray Toxicity and Risk Potential of 42 Commonly Used Formulations of Row Crop Pesticides to Adult Honey Bees (Hymenoptera: Apidae). *Journal of Economic Entomology*. 2015; doi: 10.1093/jee/tov269.

288. Desneux N, Decourtye A, Delpuech J-M. The sublethal effects of pesticides on beneficial arthropods. *Annual Review of Entomology*. 2007; doi: 10.1146/annurev.ento.52.110405.091440.

289. Hallmann CA, Sorg M, Jongejans E, Siepel H, Hofland N, Schwan H, et al.. More than 75 percent decline over 27 years in total flying insect biomass in protected areas. *PLoS ONE*. 2017; doi: 10.1371/journal.pone.0185809.

290. Ikonomopoulou M, King G. Natural Born Insect Killers: Spider-venom Peptides and Their Potential for Managing Arthropod Pests. *Outlooks on Pest Management*. 2013; doi: 10.1564/v24_feb_05.

291. Lüddecke T, Herzig V, Reumont BM von, Vilcinskas A. The biology and evolution of spider venoms. *Biological Reviews*. 2021; doi: 10.1111/brv.12793.

292. Ikonomopoulou MP, Smith JJ, Herzig V, Pineda SS, Dziemborowicz S, Er S-Y, et al.. Isolation of two insecticidal toxins from venom of the Australian theraphosid spider *Coremiocnemis tropix*. *Toxicon*. 2016; doi: 10.1016/j.toxicon.2016.10.013.

293. Smith JJ, Herzig V, Ikonomopoulou MP, Dziemborowicz S, Bosmans F, Nicholson GM, et al.. Insect-Active Toxins with Promiscuous Pharmacology from the African Theraphosid Spider

Monocentropus balfouri. *Toxins*. Multidisciplinary Digital Publishing Institute; 2017; doi: 10.3390/toxins9050155.

294. Herzig V, Ikonomopoulou M, Smith JJ, Dziemborowicz S, Gilchrist J, Kuhn-Nentwig L, et al.. Molecular basis of the remarkable species selectivity of an insecticidal sodium channel toxin from the African spider *Augacephalus ezendami. Sci Rep*. 2016; doi: 10.1038/srep29538.

295. Marsh NA. Diagnostic Uses of Snake Venom. *PHT*. Karger Publishers; 2001; doi: 10.1159/000048065.

296. Marsh N, Williams V. Practical applications of snake venom toxins in haemostasis. *Toxicon*. 2005; doi: 10.1016/j.toxicon.2005.02.016.

297. Perchuc AM, Wilmer M. Diagnostic Use of Snake Venom Components in the Coagulation Laboratory. In: Kini RM, Clemetson KJ, Markland FS, McLane MA, Morita T, editors. *Toxins and Hemostasis: From Bench to Bedside*. Dordrecht: Springer Netherlands;

298. Jay WF, Solange MTS. Approaching the Golden Age of Natural Product Pharmaceuticals from Venom Libraries: An Overview of Toxins and Toxin-Derivatives Currently Involved in Therapeutic or Diagnostic Applications. *Current Pharmaceutical Design*. 13:2927–342007;

299. Dardevet L, Rani D, Aziz T, Bazin I, Sabatier J-M, Fadl M, et al.. Chlorotoxin: A Helpful Natural Scorpion Peptide to Diagnose Glioma and Fight Tumor Invasion. *Toxins*. 2015; doi: 10.3390/toxins7041079.

300. Longbottom J, Shearer FM, Devine M, Alcoba G, Chappuis F, Weiss DJ, et al.. Vulnerability to snakebite envenoming: a global mapping of hotspots. *The Lancet*. Elsevier; 2018; doi: 10.1016/S0140-6736(18)31224-8.

301. Chippaux J-P. Snakebite envenomation turns again into a neglected tropical disease! *Journal of Venomous Animals and Toxins including Tropical Diseases*. 2017; doi: 10.1186/s40409-017-0127-6.

302. Williams DJ, Faiz MA, Abela-Ridder B, Ainsworth S, Bulfone TC, Nickerson AD, et al.. Strategy for a globally coordinated response to a priority neglected tropical disease: Snakebite envenoming. *PLOS Neglected Tropical Diseases*. Public Library of Science; 2019; doi: 10.1371/journal.pntd.0007059.

303. León G, Vargas M, Segura Á, Herrera M, Villalta M, Sánchez A, et al.. Current technology for the industrial manufacture of snake antivenoms. *Toxicon*. 2018; doi: 10.1016/j.toxicon.2018.06.084.

304. Pucca MB, Cerni FA, Janke R, Bermúdez-Méndez E, Ledsgaard L, Barbosa JE, et al.. History of Envenoming Therapy and Current Perspectives. *Frontiers in Immunology*. 2019; doi: 10.3389/fimmu.2019.01598.

305. Habib AG, Brown NI. The snakebite problem and antivenom crisis from a health-economic perspective. *Toxicon*. 2018; doi: 10.1016/j.toxicon.2018.05.009.

306. Kurtović T, Lang Balija M, Brvar M, Dobaja Borak M, Mateljak Lukačević S, Halassy B. Comparison of Preclinical Properties of Several Available Antivenoms in the Search for Effective Treatment of *Vipera ammodytes* and *Vipera berus* Envenoming. *Toxins*. Multidisciplinary Digital Publishing Institute; 2021; doi: 10.3390/toxins13030211.

307. Lamb T, de Haro L, Lonati D, Brvar M, Eddleston M. Antivenom for European *Vipera* species envenoming. *Clinical Toxicology*. Taylor & Francis; 2017; doi: 10.1080/15563650.2017.1300261.

308. Halassy B, Kurtović T, Lang Balija M. Comment on "Antivenom for European *Vipera* species envenoming." *Clinical Toxicology*. Taylor & Francis; 2018; doi: 10.1080/15563650.2018.1448402.

309. Eddleston M, Lamb T, Brvar M. Response to Halassy and colleagues. *Clinical Toxicology*. Taylor & Francis; 2018; doi: 10.1080/15563650.2018.1452253.

310. Laustsen AH, María Gutiérrez J, Knudsen C, Johansen KH, Bermúdez-Méndez E, Cerni FA, et al.. Pros and cons of different therapeutic antibody formats for recombinant antivenom development. *Toxicon*. 2018; doi: 10.1016/j.toxicon.2018.03.004.

1968

Supplementary Tables 1-5

Click here to access/download
Supplementary Material
vonReumont_etal_ModernVenomics_SuppTabls_1_5.pd
f
