# Supplementary material for: Modern venomics—Current insights, novel methods, and future perspectives in biological and applied animal venom research
Source: Gigascience. 2022 May 18;11:giac048. doi: 10.1093/gigascience/giac048 (PMC9155608; doi:10.1093/gigascience/giac048)
Supplement: giac048_Supplemental_File [file giac048_supplemental_file.pdf]

Table S1. Overview of current protocols and methods to collect animal venoms.

Higher Clade	Subgroup	Method	Reference
**Snakes**	Solenoglyphous	Traditional milking	Venomous Reptiles and Their Toxins. Evolution, Pathophysiology and Biodiscovery, 2015, Oxford Press, Chapter Research Methods, ISBN 978-0199309399
	Proteroglyphous	Traditional milking	
	Opisthoglyphous	Injection of pilocarpine and pipetting from fang	
**Lizards**	Helodermatidae	Bite on tube and collection of dripping venom	
	Anguimorpha and Igua	Injection of pilocarpine and pipetting from mouth	
**Amphibians**	Gymnophiona	Pilocarpine injection and manual release post-euthanization	https://www.sciencedirect.com/science/article/pii/S2589004220304193
**Mammals**	Vampire bats	Injection of pilocarpine and collection of saliva	https://www.sciencedirect.com/science/article/abs/pii/S1874391913003102?via%3Dihub
	Loris	Swabs	https://www.sciencedirect.com/science/article/abs/pii/S0041010114006291?via%3Dihub
	Shrews	Dissection of venom glands	https://www.pnas.org/content/101/20/7542
	Platypus	Manual release from stinger	http://dx.doi.org/10.1351/PAC-CON-11-08-18
	Solenodon	Bite on tube and collection of dripping venom	https://www.pnas.org/content/116/51/25745
**Cartiligous fish**	stingray	Scratch of venomous tissue from stinger	https://www.sciencedirect.com/science/article/abs/pii/S0041010114003420?via%3Dihub
**Bony fish**	Stonefish	Emptying of venom glands with syringe	https://pubmed.ncbi.nlm.nih.gov/27867093/
	Lionfish	Extract from spine	https://www.sciencedirect.com/science/article/abs/pii/0041010189900688?via%3Dihub
	Fangblennys	Bite in Swabs	https://www.sciencedirect.com/science/article/pii/S0960982217302695#app2
	Catfishes	Extract from spine	https://bmcecolevol.biomedcentral.com/articles/10.1186/1471-2148-9-282#Sec7
	Weeverfish	Dissection of venom glands	https://www.sciencedirect.com/science/article/abs/pii/004101019290503W?via%3Dihub
	Flatheads	Extract from spine	https://www.mdpi.com/2072-6651/9/2/67/htm
	Sea breams	Extract from spine	https://www.mdpi.com/2072-6651/9/2/67/htm
	Sea chubs	Extract from spine	https://www.mdpi.com/2072-6651/9/2/67/htm
	Mullet	Extract from spine	https://www.mdpi.com/2072-6651/9/2/67/htm
	Fish in general	Sponge in a tube method	https://www.frontiersin.org/10.3389/conf.FMARS.2016.04.00124/event_abstract
**Sponges**	Sponges	Organic extraction	https://www.scielo.br/j/jvatitd/a/N8yHwps9XZGF8F89QbTQ4Sz/?lang=en
**Cnidaria**	Anemones	Extract from homogenized tentacles	https://www.sciencedirect.com/science/article/abs/pii/0041010182901891?via%3Dihub
		Pressing over collection beakers	https://pubmed.ncbi.nlm.nih.gov/1982253/
		Extract from homogenized full body	https://pubmed.ncbi.nlm.nih.gov/2568126/
	Jellyfish	Extract from homogenized tentacles (beads)	https://www.nature.com/articles/s41467-019-09681-1
		Extract from homogenized tentacles (sonication)	https://pubmed.ncbi.nlm.nih.gov/27461980/
		Chemical induced discharge	https://www.mdpi.com/2072-6651/7/3/936
**Echinoderms**	Starfish	Extract from homogenized spines	https://www.jstage.jst.go.jp/article/jts1976/21/1/21_1_11/_pdf/-char/en
	sea urchins	Aqueuous extraction of pedicellariae	https://www.sciencedirect.com/science/article/abs/pii/0041010184900795
**Moluscs**	Cephalopods	Dissection of venom glands	https://science.sciencemag.org/content/199/4325/188
		Plastic bag method	https://www.sciencedirect.com/science/article/abs/pii/0041010172900098?via%3Dihub
	Cone snails	Injection into mock prey (tube)	https://journals.plos.org/plosone/article?id=10.1371/journal.pone.0098991#s2
		Dissection of venom glands	https://www.mdpi.com/1660-3397/17/8/432/htm
		Injection into mock prey (mock fish)	https://pubmed.ncbi.nlm.nih.gov/7673220/
	Terebridae	Dissection of venom glands	https://journals.plos.org/plosone/article?id=10.1371/journal.pone.0094122#s4
**Annelids**	Polychaetes	Mechanical stimulation	https://www.nature.com/articles/s41598-018-26031-1#Sec8
**Polychaetes**	Blood worms	Extract from homogenized glands (Milking also possible)	https://www.sciencedirect.com/science/article/abs/pii/0305049175902941
**Annelids**	Leeches	Mock feeding	https://bmcgenomics.biomedcentral.com/articles/10.1186/s12864-020-6748-0#Sec29
		Regurgitation	https://journals.iium.edu.my/ejournal/index.php/iiumej/article/view/156/196
**Arachnids**	Funnel web spiders	Pipetting from Chlicerae	https://bmcgenomics.biomedcentral.com/articles/10.1186/1471-2164-15-177
	Tarantulas	Electrostimulation	https://www.sciencedirect.com/science/article/pii/S0041010108005813
	orb weaver spiders	Dissection of venom glands	https://www.mdpi.com/2218-273X/10/7/978
	Widow spiders	Electrostimulation	https://www.jove.com/de/t/51618/extraction-venom-venom-gland-microdissections-from-spiders-for
	Wolf spiders	Mock bite	https://www.scielo.br/j/jvatitd/a/XgnVhx3yKG3776yQSfR7rLh/?format=html
		Mock bite and electrostimulation	https://www.mdpi.com/2072-6651/12/8/501/htm#B32-toxins-12-00501
	Daddy long-leg spiders	Electrostimulation	https://www.frontiersin.org/articles/10.3389/fevo.2019.00256/full
**Crustaceans**	Remipedes	Dissection of venom glands	https://www.mdpi.com/2072-6651/9/8/234/htm
**Insects**	Parasitoid wasp	Dissection of venom glands	https://www.ncbi.nlm.nih.gov/pmc/articles/PMC6950128/
	Bees (and other aculeat	Electrostimulation	https://www.sciencedirect.com/science/article/abs/pii/S0731708516300073?via%3Dihub
	Ants	Dissection of venom glands and electrostimulation	https://pubmed.ncbi.nlm.nih.gov/28118015/
		Hive extraction	https://www.sciencedirect.com/science/article/pii/S0041010112008380
	Robber flys	Dissection of venom glands	https://www.mdpi.com/2072-6651/10/1/29/htm
	Assasin bugs	Harassment and electrostimulation	https://www.mdpi.com/2072-6651/10/1/29/htm
		Mock bite	https://www.mdpi.com/2227-9059/9/7/819
	Caterpillars	Mock sting	https://www.pnas.org/content/118/18/e2023815118.long

Table S2. Examples of approved venom peptides or derived drugs for human use in clinics

Species	Drug Molecule & Brand name	Pharmacology&Mechanism of Action & Molecular target	Indication&Use	Year approved (US FDA) & Reference
Snake *(Bothrops jararaca)*	Captopril (Capoten®)	Inhibitor of angiotensin converting enzyme (ACE)	Hypertension, cardiac failure	1981, [1]
Snake *(Bothrops jararaca)*	Enalapril (Vasotec®)	Inhibitor of angiotensin converting enzyme (ACE)	Hypertension, cardiac failure	2001, [1]
Snake *(Echis carinatus)*	Tirofiban (Aggrastat®)	Glycoprotein IIb/IIIa inhibitors	Heart attack	1999, [2]
Snake *(Sistrurus miliarius)*	Eptifibatide (Integrilin®)	Glycoprotein IIb/IIIa inhibitors	Acute coronary syndrome	1988, [3]
Snake *(Bothrops atrox* and *Bothrops moojeni)*	Batroxobin (Defibrase®/ Plateltex-Act®/ Reptilase®)	Converts fibrinogen into fibrin through the release of fibrinopeptide A from fibrinogen	Stroke, pulmonary embolism, deep vein thrombosis, myocardial infarction and perioperative bleeding.	Outside USA only [2, 4]
Snake *(Naja naja atra)*	Cobratide (Ketongning, cobratoxin)	Blockage of nicotinic receptors neuropathic headache	Chronic arthralgia, sciatica	1998 [5]
Cone snail *(Conus magus)*	Ziconotide (Prialt®)	Blocks CaV2.2 voltage-gated calcium channel	Severe chronic pain	2004, [1]
Lizard *(Heloderma suspectum)*	Exenatide (Byetta®)	Glucagon-like peptide-1 receptor agonist	Type 2 diabetes mellitus	2005, [1]
Lizard *(Heloderma suspectum)*	Lixisenatide (Lyxumia®, Adlyxin®	Glucagon-like peptide-1 receptor agonist	Type 2 diabetes mellitus	2016, [1,4]
Leech *(Hirudo medicinalis)*	Bivalirudin (Angiomax)	Inhibits platelet aggregation and the coagulation cascade	Stroke, deep vein thrombosis and pulmonary embolism	2000, [1]
Leech *(Hirudo medicinalis)*	Desirudin (Iprivask®)	Selective and near irreversible inhibitor of thrombin	Prevention of venous Thrombotic events	2003, [1]

Table S3. Examples of venom peptides or derivatives in clinical trials

Species	Molecule/Brand name	Pharmacology & Mechanism of Action	Indication & Use	Reference
Honeybee *(Apis mellifera)*	Whole venom (Apitox®)	Antiinflammatory action; alteration of the immune response via antigen competition	Pain associated with osteoarthritis and multiple sclerosis	Phase III [6]
Snake *(Calloselasma rhodostoma)*	Ancrod (Viprinex®)	Anti-coagulant	Stroke, cerebral ischemia, brain infarction	Phase III [7]
Cone snail	v-Conotoxin MrIA (Xen2174)	Inhibits noradrenalin transporter	Chronic pain	Phase II [7]
Sea anemone (*Stichodactyla helianthus*)	ShK-186 (Dalazatide)	Blocks KV1.3, Kv1.1 and Kv1.6voltage-gated potassium channel	Autoimmune Diseases (psoriatic arthritis, multiple sclerosis, lupus, rheumatoid arthritis)	Phase II [8]
Scorpion *(Leiurus quinquestriatus quinquestriatus)*	Chlorotoxin (TM-601)	Binds MMP2, Annexin A2 and CLC-3 chloride cannels on surface of glioma Cells and other tumors of neuroectodermal origin, impairing invasion ability	Glioma, Intraoperative imaging of solid cancer cells using as tumor paint	Phase I [7]

Tick (Ornithodoros moubata)	Nomacopan (Formerly Coversin)	Acts on complement component C5 responsible for in inflammatory and neuropathic pain	Moderates severe atopic keratoconjunctivitis as corneal and eye surface disease.	Phase III

Table S4. Examples of venom peptides with neuroactive properties

Species	Molecules/Brand name	Translational perspectives	References
Bungarus sp.	Beta-bungarotoxin	The phospholipase activity of PLA2 from the β-BuTx B chain has shown great promise for its potent non-steroid anti-inflammatory effects. Moreover in addition to the above-mentioned capabilities with their potential downstream effects on neuronal and synaptic processes, changes in the activity of endogenous PLA2 are thought to be crucial in inflammatory processes related to numerous acute and chronic neurological disorders accompanying neurodegenerative diseases, such as Alzheimer's and Parkinson's diseases as well as brain tumors.	[9,10]
Dendroaspis sp.	Dendrotoxins	Because reduction ofα-DTX binding by brain tissue indicates the degeneration of synaptic terminals and connections enriched with KV1 channels, α-DTX binding has been used as a direct biomarker for detecting the integrity of synaptic connections and neural circuits. In Alzheimer's disease, for example, α-DTX has been applied to detect the extent of synaptic loss in hippocampal tissue. Using a similar approach, age-related changes in synaptic density have been shown in the rat brain. DTXs have been also instrumental in targeting and functional validation of the unique juxta-paranodal expression of KV1 channels in demyelinated axons. In denuded axons of optic nerves, for instance, it was shown that α-DTX restores the functionality of demyelinated nerves by blocking KV1.1 containing channels displaced into nodal and intermodal regions. Finally, through the use of DTXs and synthetic blockers of KV1 channels, demyelination-related remodeling of KV1 with emergence of a novel KV1.1 homotetramer has been shown in optic nerve axons, which might be of major relevance for diagnosis and treatment of multiple sclerosis and other axonal disorders associated with myelin loss.	[9,10,11]
Elapid snakes (e.g., cobras, mambas, kraits, sea snakes, etc.)	-Neurotoxins	a-bungarotoxin enabled the first isolation and characterization of nAChR, contributing towards unraveling mechanisms of neuromuscular diseases, such as myasthenia gravis. k-Bungarotoxin has also been used for deciphering molecular mechanisms of long-term potentiation and synaptic memory, whereas the three-finger muscarinic toxins (MT1 and MT2 from various African mamba venoms) selective for M4 muscarinic receptors (mainly found in the central nervous system) have been used for induction of retrograde amnesia and memory-deficit animal models. Cobratoxin from Thailand cobra (*Naja kauothia*) has been considered as therapeutic candidates for the treatment of adrenomyeloneuropathy and multiple sclerosis, whereas a-cobratoxin has shown	[9,10]

		promise as a potential anticancer drug in non-small cell lung cancer	
Dendroaspis sp.	Fasciculins	Low mobility and inability to diffuse far from the site of injection render fasciculins suitable for studies in the central nervous system, using microinjections into specific brain regions, causing localized and long-lasting inhibitory effects. Given that fasciculins can be labeled with I^{125}, they have also been applied as probes for AChE in quantitative autoradiography. Likewise, in developmental studies, fasciculins have been utilized to investigate the role of AChE in governing the outgrowth of neurites from nerve cells, an effect attributable to the hydrolytic action of enzymes on ACh as well as its role as an adhesion factor. Similarly, in neuroblastoma cells transfected to express AChE, treatment with fasciculins caused a significant decrease in both the number of neurites and their length. Even though the poor penetration of fasciculins to the brain and spinal cord impose major limits for their utility as enhancers of cholinergic drive in central synapses, the use of different types of natural AChE blocker for countering cognitive deficit and memory loss in Alzheimer's disease has been widely discussed	[9,10]

Table S5. Antivenom varieties and properties [12]

<table>
<tr><th>Origin</th><th>Specificity</th><th>Mode of Precipitation</th><th>Further purification</th></tr>
<tr><td rowspan="3">1. Ovine-derived (IgG, Fab fragments/ papain digestion);
2. Equine-derived (IgG, $F(ab')_2$ fragments/ pepsin digestion);
3. Camelid-derived (experimental)</td><td>• Monospecific (25 antivenoms available on the Market)
• Polyspecific (70 WHO registered antivenoms)</td><td>• Ammonium sulfate precipitation
• Caprilic acid precipitation</td><td>• Aqueous two-phase systems (ATPS)
• Ion exchange chromatography</td></tr>
<tr><td colspan="3">Liquid formulation with/without preservatives (phenol/cresol)</td></tr>
<tr><td colspan="3">Freeze-drying or lyophilization</td></tr>
</table>

References

1. US Food and Drug Administration. Drugs@FDA: FDA approved drug products. 2021; Available at: https://www.accessdata.fda.gov/scripts/cder/daf/ (Accessed on 31th of October 2021).
2. Abd El-Aziz TM, Garcia Soares A, Stockand JD. Snake Venoms in Drug Discovery: Valuable Therapeutic Tools for Life Saving. *Toxins*. 2019; doi: 10.3390/toxins11100564.
3. Robinson SD, Undheim EAB, Ueberheide B and King GF. Venom peptides as therapeutics: advances, challenges and the future of venom-peptide discovery, *Expert Review of Proteomics.* 2017; doi: 10.1080/14789450.2017.1377613.
4. Bordon KCF, Cologna CT, Fornari-Baldo EC, Pinheiro-Júnior EL, Cerni FA, Amorim FG, Anjolette FAP, Cordeiro FA, Wiezel GA, Cardoso IA, Ferreira IG, de Oliveira IS, Boldrini-França J., Pucca, MB, Bald, MA, Arantes EC. From Animal Poisons and Venoms to Medicines: Achievements, Challenges and Perspectives in Drug Discovery. *Front. Pharmacol*. 2020; doi: 10.3389/fphar.2020.01132.
5. Zhang Y. Why do we study animal toxins? *Zoological Research* 2015; doi: 10.13918/j.issn.2095-8137.2015.4.183.
6. US National Library of Medicine. 2021; ClinicalTrials.gov, Available at: https://clinicaltrials.gov/ (Accessed on 31th of October 2021).

7. Vetter I, Davis JL, Rash LD, Anangi R, Mobli M, Alewood PF, Lewis RJ, King GF. Venomics: a new paradigm for natural producs-based drug discovery, *Amino Acids*. 2011; doi: 10.1007/s00726-010-0516-4.
8. Coulter-Parkhill A, McClean S, Gault VA, and Irwin N. Therapeutic Potential of Peptides Derived from Animal Venoms: Current Views and Emerging Drugs for Diabetes. *Clinical Medicine Insights:Endocrinology and Diabetes*. 2021; doi:10.1177/11795514211006071.
9. Ayvazyan NM, O'Leary VB, Dolly JO, Ovsepian SV. Neurobiology and therapeutic utility of neurotoxins targeting postsynaptic mechanisms of neuromuscular transmission. *Drug Discov.* 2019; doi: 10.1016/J.DRUDIS.2019.06.012.
10. Ovsepian SV, O'Leary VB, Ayvazyan NM, Al-Sabi A, Ntziachristos V, Dolly JO. Neurobiology and therapeutic applications of neurotoxins targeting transmitter release. *Pharmacol. Ther.* 2019; doi:10.1016/J.PHARMTHERA.2018.08.016.
11. Harvey AL, Twenty years of dendrotoxins. *Toxicon*, 2001; doi: 10.1016/s0041-0101(00)00162-8.
12. WHO 2021, https://www.who.int/bloodproducts/snake_antivenoms/snakeantivenomguideline.pdf (Accessed on 31th of October 2021).
