# Supplementary material for: Modern venomics—Current insights, novel methods, and future perspectives in biological and applied animal venom research
Source: Gigascience. 2022 May 18;11:giac048. doi: 10.1093/gigascience/giac048 (PMC9155608; doi:10.1093/gigascience/giac048)
Supplement: giac048_Response_to_Reviewer_Comments_Original-Submission [file giac048_response_to_reviewer_comments_original-submission.pdf]

Author's Response To Reviewer Comments

Close

Reply to Reviewer 1

Reviewer #1: This review aims to synthesize the new and diverse evolutionary and more applied approaches used to characterizing venom diversity across a suite of venomous organisms. Overall, I like the structure of the review, from sample collection to data generation, although I did find the specifics on analysis in some sections lacking (e.g., genome assembly). I also found it strange that the review went from protein to RNA to morphology (section 6) rather than first to DNA/genomics (section 7). I think the flow would be improved if the central dogma outline was retained with morphology being section 7 (and genomics 6), but that is merely a suggestion. I also generally like the figures; I think they are well done. I outline a few major and minor concerns below, but the largest concern is that the authors exclude several recent, high-profile and relevant papers in their discussion of different techniques and questions. This can easily be addressed, but their exclusion was puzzling given their relevance to the particular sections.

MAJOR:

1. In section 2.1 the authors state how, until recently, most venomic studies have focused on snakes, spiders, and scorpions. I agree, but it would be useful to see a figure showing the number of published studies on various taxonomic groups to more convincingly demonstrate this point and show this knowledge gap. Over the past 10 years, for example, how many studies on snakes, scorpions, snails, bees, centipedes, etc.?
Reply:
We see the point of reviewer 1 and added a new figure (now figure 2) that displays the number of venomous species and reviewed venom proteins that are available in the Unitprot/ToxProt database. This gives a more precise idea of the taxon bias in venomics. The literature search is indeed a good idea, but quite difficult to conduct and not really to automate due to persistent misleading results (False positives) based on search terms.

2. Figure 3 caption needs to be improved. Currently only a title sentence is supplied with no description of the figure itself.

Reply:
We include now a more detailed figure capture as suggested.

3. The authors mention several new techniques but do not cite venomic studies using such techniques. For example, they mention ATAC-seq in section 5.2, but do not cite a recent venom study that used this approach:
Margres, M. J., Rautsaw, R. M., Strickland, J. L., Mason, A. J., Schramer, T. D., Hofmann, E. P., ... & Parkinson, C. L. (2021). The Tiger Rattlesnake genome reveals a complex genotype underlying a simple venom phenotype. Proceedings of the National Academy of Sciences, 118(4).

Reply:
Thanks for this reference, we missed this indeed and include and highlight it now briefly.

Similarly, in section 7, they cite a number of new snake, spider, and cone snail genomes but exclude the above citation as well as the other rattlesnake genome that was recently published:
Schield, Drew R., Daren C. Card, Nicole R. Hales, Blair W. Perry, Giulia M. Pasquesi, Heath Blackmon, Richard H. Adams et al. "The origins and evolution of chromosomes, dosage compensation, and mechanisms underlying venom regulation in snakes." Genome research 29, no. 4 (2019): 590-601.
I am less familiar with spiders and cone snails, but in reviews like this, these types of works certainly need to be included. The authors should make sure all recent, high-quality genomes are cited. Perhaps a table would be beneficial.

Reply:
We checked for further and new publications and include now both references given by the reviewer, and a recent cone snail genome paper we missed (Peng e tal. 2021).

4. Similarly, the authors discuss venom gene origin in section 7.1 but ignore three papers from Sean Carroll's group that are perhaps our best current examples of such work:

Dowell, N., Giorgianni, M., Kassner, V., Selegue, J., Sanchez, E., & Carroll, S. (2016). The deep origin and recent loss of venom toxin genes in rattlesnakes. Current Biology, 26(18), 2434-2445.
Dowell, N. L., Giorgianni, M. W., Griffin, S., Kassner, V. A., Selegue, J. E., Sanchez, E. E., & Carroll, S. B. (2018). Extremely divergent haplotypes in two toxin gene complexes encode alternative venom types within rattlesnake species. Current Biology, 28(7), 1016-1026.
Giorgianni, M. W., Dowell, N. L., Griffin, S., Kassner, V. A., Selegue, J. E., & Carroll, S. B. (2020). The origin and diversification of a novel protein family in venomous snakes. Proceedings of the National Academy of Sciences, 117(20), 10911-10920.
These papers certainly need to be included and discussed in this section.

Reply:
We agree and include the cited papers. The Dowell 2016 reference shows also some erroneous assumptions overlooking orphan exons that are discussed within the additional citation of Jackson and Koludarov 2021.

5. Why are no evolutionary studies of venom function using in-vivo assays discussed on lines 753-760? The entire bioassay section ignores evolutionary implications, which, according to the introduction was half of the focus of the review, and much work has been done in this area. Further, although in vitro and ex vivo assays can provide more precise information regarding the interaction of specific tissues and toxins as the authors describe, they do not characterize the overall ecological interaction between predator and prey (regardless of who is venomous) that in vivo assays do. Each has their strengths and place, and this should be discussed.

Reply:
In this section 9 we focused as stated in the beginning paragraph on applied aspects. However, we include now ecological aspects asked for by the reviewer, which makes this section indeed more integrative, though slightly longer. We briefly highlight now differences in the assays in regards to ecological questions and their importance to asses predator prey interactions for example (lines 786-790, 806-807, 821-826). We include also new references which should overall link the matter of assays for applied research better with the ecological context.

MINOR:

Typo in Abstract lines 106-107: "to identify more efficiently" should be "to more efficiently identify", please fix.

Reply:
Changed.

Line 118: "ensuring the fitness" strikes me as inappropriate; perhaps "that it is a critical component of the fitness" instead

Reply:
Thanks for the suggestion, it is rephrased.

Line 128: "make" should me "making"

Reply:
We change this.

Line 140: "challenge" should be "challenges"

Reply:
Is changed.

Lines 416-417: "identify as well house-keeping genes" should be "identify house-keeping genes as well", and "guarantee the venom secretion" again seems inappropriate; perhaps "participate in venom production"?

Reply:
Thanks for this suggestion, we changed this phrasing to assist venom production.

Line 497: comma after period at end of line

Reply:
Corrected.

Lines 753-754: Grammar - needs to be "posing", "being", and "often not being"

Reply:
Thanks, we corrected this.

Within section 9.1, the subsections are not numbered. Are they supposed to be?

Reply:
Yes, indeed, we use italics caption instead to make it easier to read.

Typo section title line 899: Immunomdulation

Reply.
Typo is corrected.

- - -

Reply to Reviewer 2

Reviewer #2:

Review - Modern venomics - Current insights, novel methods and future perspectives in biological and applied animal venom research

The authors present a thorough review that spans the field to the bench to the bedside where venom research is concerned. The review covers new ground by mentioning the application of the latest in omic technologies and other new technological developments, while also serving as a 'review of reviews' as several aspects of venom biology and translational research have been extensively reviewed previously. They appropriately are scant on previously reviewed material while collating the pertinent facts and figures into a review that can serve as an up-to-date guide for anyone getting into venomics in the 2020s and standing on more than a century of work. I provide comments below on clarity and content improvement for several sections of the manuscript. Overall, a really nice piece of work that will help solidify and move the field.

Major:
Line 97-98: It will be more impactful to justify/explain exactly why the combination of "the evolutionary and the applied perspective" will be interesting or useful in the future, rather than just state that you are doing it.

Reply:
We rephrased that as suggested.

Line 127-128: There are still comparatively few studies supporting a clear role for coevolution (defined as reciprocal selection between predator and prey and minimally suggested by local or species-level adaptation of venom or venom resistance) and these are not cited here even. So, this statement is a vast overstatement. I think coevolution plays a role in many (maybe most) venoms, but the way you state this here implies a large body of support that does not exist. Build the statement better with citations and context. An alternative to coevolution is that venom evolves to attack physiological targets and then prey, because maybe they have large R selected populations, do not evolve resistance due to

trade-offs.

Reply:
We agree with the reviewer and rephrased more carefully by extending a bit on the still little studied co-evolutionary aspects (ll. 143-154). We address now as suggested the few studies that work on venom resistance arms races between predator-prey as co-evolutionary adaptation.

Line 164-173: You take too long to get to the important point here, and make the point very obliquely. State your thesis at the top of the paragraph: "An understanding of venomous animal diversity is crucial to understanding venomous animal diversity and novel protein functions. Yet, taxonomic expertise in many venomous animal groups has been on the decline".

Reply:
We agree and shortened here. We also address earlier the point of animal venom diversity.

Line 184 to 188: This is too important of a point set to include in a single long sentence. Break up proposed benefits and costs of Nagoya into two sentences. Doing so will let you expand more specifically on what you mean by hinderance of "translational applications". Do you mean the speed with which they can be discovered and enacted at scale? Additionally, you present these points without really taking a stance, even a nuanced one. For example, given that the WHO wants to half snakebite by 2030, if Nagoya protocol is perceived to slow progress there, then two international agreements/bodies are in potential conflict. Right now this paragraph merely raises in the most vague terms some benefits and costs of the very important Nagoya protocol, and so leaves the reader to draw whatever conclusions they want to without additional context that could easily be made available by the authors. I believe this is the first time I have seen Nagoya raised in a venom paper, so the treatment needs to be thorough, thoughtful, and nuanced or not at all.

Reply:
We split the sentence of objections in two, as suggested and expand more on hurdles. We also give slightly more background by trying to avoid to be long.

Line 222 - 257: It would be helpful to readers new to metabolomics to expand a bit on two things: First is to methodologically differentiate metabolomics from the better-known proteomics. What specific molecule types can be detected and how? Second, can you talk in more physiologic detail about at least one of the toxins discovered through metabolomics? For example, you mention the acylpolyamines in line 240 and then several other molecules in lnes 248-250. Were any of these subsequently tested in vivo in mice or insect models and show to be toxic? If show, recitation of those specific details in brief would benefit this section in terms of biological reality.

Reply:
We see the reviewer’s point and expand now the differences between metabolomics and proteomics, but try to keep it as short as possible. The metabolites that are known so far are named and briefly discussed. Largely the function of metabolites in venoms is still unknown. Identifying their role in connection with the proteomic profile is a present challenge of the venom research, which we highlight more clearly. The study of small molecules in the venom has so far been focused on the identification of molecules (Peptides, Proteins) that have been known to be toxins above some concentrations or other drastic molecules that could play a role as potential drugs. It is a significant current objective of the venom community to try to understand the richness of the metabolic profile. The systematic investigation of the vastness of the venom metabolites and their function has not been done so far but is in process within the new research field metabolomics. This is pointed out better now.

Lines 361-372: The authors are focusing heavily on the utility of RNA-seq data for making databases for proteomics. However, there is an entire subfield of venom biology that uses the RNAseq data alone to study differential expression of venom genes. The authors should highlight and discuss this as it is the most common use of venom rna-seq data.

Reply:
We agree and state that also later in sections 6.3 and 7. However, we mention now in a sentence the importance of RNA-seq to address differentially expressed venom genes and refer then to sections 6.3 and 7.

Lines 455 - 545: This section on morphological analysis of venom production is generally well-written

and informative. I think it currently lacks a broader justification for why this should be an interesting topic of investigation. The previous sections set up venom variation as important for various biological and translational reasons, but why is venom morphology/spatial analysis of expression interesting? I am intrinsically interested, but would ask the authors to expand more specifically on what they think we can learn by integrating morphology and venomics, beyond the very general explanations they give (e.g. lines 542-545). What hypotheses exist and what can be tested? Can we discover new venom functions or new venom proteins based on spatial analyses? What else? Without this information this otherwise well-done and interesting section sticks out as an "add-on" to the rest of the paper.

Reply:
We expand and add more details now and the context why morphology is important to understand the often not uniform venom expression and secretion. In section 6.2 we give a couple of examples of the discoveries that can be made by integrating morphological and molecular data, including identifying previously unrecognized parts of the venom apparatus and novel venom producing tissues, as well as revealing the structural organization and compartmentalization of venom glands (lines 599-602). Nevertheless, we have added a paragraph at the beginning of Section 6.1 to expand on why is important, and what can we learn from integrating morphology and venomics (lines 552-558).

Lines 819-826: most readers will likely have only heard of these methods, if that. It would be helpful to the reader to give a very brief idea of how these various methods might be useful in venom biology. Otherwise, it is just a list of methods that could equally be substituted for any other. You could summarize in a table that has columns of "method" and "proposed use

Reply:
We agree and expanded Section 9.2 to give a better idea of the methods and their use to understand venom biology better.

References cited: Likely due to my own reading biases, a disproportionate citation of venom research from the Eastern hemisphere is noted. The comprehensiveness of the review would benefit from more representative considerations of the work done given that the review addresses global challenges.

Reply:
We see the point and assure that this was not our intention. After the inclusion of several suggested citations including new ones on snakes, cone snails and spiders that we had missed and new citations linked to our revision of section 1 and 2, we give now much more balanced references.

Length: I found the review to be a bit long. I think with shoring up of the writing with an eye for conciseness, the authors could reduce the length of the paper by 10-20%, while still being as informative but more impactful.

Reply:
We definitely see the point and tried to shorten more - where possible.

Minor:
Line 92: "taxasuch" needs a space. Many other combined words are noted throughout the paper and need to be search out.

Reply:
Changed. We did replace other combined words throughout the manuscript as well.

Line 92-96: Run-on sentence can be split and simplified to increase impactfulness: "There are many outstanding questions on the evolution of toxin arsenals, such as how venom genes originate, how venom contributes to the fitness of venomous species, and which modifications at the genomic, transcriptomic and protein level drive their evolution. These questions have received particularly little attention outside of snakes, spiders, and scorpions".

Reply:
Thanks for the helpful suggestion, we rephrased the sentence.

Line 97: Reword "translational research aspects", as it is not really clear what you mean.

Reply:

We rephrased this sentence.

Line 106-107: "more efficiently identify"

Reply:
Phrase is changed accordingly.

Line 107: What is an "adapted system". Seems like you mean in vitro, but can be confused with evolutionary adaptation.

Reply:
We rephrased to expression system to clarify this.

Line 118: "such an effective adaptation" is a jump in logic. It might have evolved 100 times because it is easy (i.e. a small jump in the fitness landscape using exisiting genes and glands) rather than effective. It is okay to say venom is everywhere or ubiquitous in natural ecosystems or something similar.

Reply:
Indeed, we agree and use the phrase „successful adaptation" now.

Line 121: Again, I don't think there is evidence that venom ensures the stability of food webs. If you want to argue that, for example, ants, bees, jellyfish and other very common important species are important to ecosystem functioni, and therefore than venoms are involved at a basic level in community interactions, that makes more sense. But this is too straightforwardly stated without a corresponding citation or appropriate context.

Reply:
Good point, we rephrased here and use now the term ‚ecological networks'.

Line 136-137: Written somewhat informally. How about: "The modern field of venomics integrates the evolution, ecology, and translational research on venoms".

Reply:
We rephrased this sentence.

Line 137-138: Sentence seems to be contrasting venomics with snakebite, but venomics certainly also concerns itself with better snakebite treatments. Overall, the sentence starting with "However" seems unnecessary and a bit flowery. I would remove it.

Reply:
We find this phrasing is a matter of personal preference, but we deleted the sentence, also to shorten the manuscript as suggested.

Line 140: "challenges"

Reply:
Changed.

Line 141: I think "Neozoic" is defining a broader time range than is desired here. Do you simply mean to state that "Additionally, climate change may facilitate movement of venomous species into new areas, posing risks to previously unexposed humans, native species, and livestock"? Honestly, I think the previous sentence would be a better end to this paragraph and this climate change sentence can be deleted.

Reply:
We changed neozoic (which was indeed not meant) with neobiota. However, we like to leave this sentence, it is directly linked to the sentence before and climate change impacts on envenomation problematics by migrating venomous species.

Line 148: relace "all the way towards the" with "to". Then put a period after research, so that the last sentence just reads "We aim to create a blueprint for future venomics studies and a roadmap toward new methodological perspectives".

Reply:
Thanks for the suggestion, we replaced this.

Line 153: "evolutionary trait" is a vague and somewhat useless phrase. I think you could just short to "the importance and impact of venom". It is already implied that it is a trait, and it evolves.

Reply:
We agree and changed this.

Line 218: What is meant by "pre-purified". This could be fractionation or just spinning out large cell debris, or something else. Explain further please.

Reply:
We rephrased this and explained further, it is indeed meant as presumed by the reviewer, only a pre-filtering step from tissue-remains is addressed here.

Line 231: Delete "but gradually picking up"

Reply.
Deleted.

Line 237: The mentioned 1936 work does not appear to be cited.

Reply:
We rephrased and shortened this sentence deleting no-relevant parts, by doing this the part with the missing citation from 1936 was deleted as well.

Line 264: Delete "nowadays".

Reply:
deleted.

Line 435: Authors should probably cite the single ATAC-seq containing venom paper:
https://www.pnas.org/content/118/4/e2014634118

Reply:
Thanks a lot for the suggestion, the refence is now included.

Line 899: Header misspelled

Reply:
Corrected.

Line 905 : "rattlesnake" is on word

Reply:
We corrected this.

Lines 911-918: Several citations are not numbered here.

Reply:
Thanks to point this out, they are corrected now.

Line 927: It is an oversell to say that they ARE the future. Tone down.

Reply:
we agree and changed this.

Line 944: MinION may require a trademark symbol?

Reply:

We added the trademark symbol.

Line 980: This sentence does not make sense. Something missing or in need of rewording.

Reply:
We rephrased this sentence to make it more clear

Line 1003: "excising"

Reply:
Thanks. It's corrected

Figures:
Figure 1. Beautiful figure overall! I would argue that regulatory evolution is a major basic research component that you are missing hre.

Reply:
Thanks for the compliment and suggestion, regulatory evolution is now added.

Figure 2. The fading color on the connecting tracks make it more difficult to discern the connections being drawn. Use solid color throughout.

Reply:
We would argue this is rather personal preference, but changed this as suggested.

Best,
Matt Holding

- - -

Reviewer #3:

The authors provided a very extensive and thorough review of modern venomics, focusing on current approaches and potential applications. The authors provided a robust framework to guide the readers through work that has been done in this area with broad taxonomic representation. This review brings attention to areas of modern venomics that have been largely ignored, for example the importance of taxonomic expertise, the use of metabolomics, and limitations to widely accepted comparative venomic methods. The authors also emphasize the drawbacks the field faces, such as the detriment of clinical applications of treating envenomation in areas where it is badly needed, the importance of data sharing and collection permits, and the roadblocks we currently face with high throughput functional annotation.

The manuscript is well written (although I have some very minor comments in the attached manuscript draft) and would be of broad interest to the GigaScience readers, as well as bring some venom researchers to the GigaScience journal that I feel has a lot the field of comparative venomics with big data and analysis. In the attached pdf I have some minor comments with regards to editing, and suggest the authors review the journal preference for the use (or disuse) of the Oxford comma as it seems to be used inconsistently. Although there is a large number of venom review papers out in the current literature, I feel that this review is a substantial and necessary addition to the field of modern comparative venomics. It will be a positive and significant contribution to the field of modern venomics.

Reply to reviewer 3

We thank reviewer 3 for comments and suggestions. Most are considered and included in the revised manuscript as listed:

1.) Abstract, p. 4, l. 94 – run on sentence is changed.

2.) Background, p. 5, l. 128 – make changed to making

3.) Background, p.8, l. 168 - we did not include the suggested reference here, because we shortened the paragraph (ass suggested by reviewer 2) and deleted the reference to envenomations by spiders etc. The focus here is the collection of all species.

4.) 4. Proteome analyses of crude venoms, p. 13, l. 264 – statement revised as suggested.

5.) 4.2, p. 15, l 332 - the comment on the limiting factor to identify protein families of unexplored venom organisms is added.

6.) p.19, l 411 – The comment on the downside of commercial software is added. However, we have to state here that most analyses demand more bioinformatics skill and scripts that are rather adapted for R, Perl or python environment and not for gGUI-based commercial software.

7.) p.20, l. 416 – Rephrased, we meant here house-keeping genes that assist venom secretion but are not per se venom proteins.

8.) p. 20, 424 – we rephrased this sentence to make clear that the reference refers to scRNA-seq in general.

9.) p. 20, l 49-430 – this could be the case but should be tested in the future in other vneomous taxa. We added this clarification.

10.) p. 27, ll 551-552 – Given the length of the article and the focus on recent, general venomous species, we prefer to not include museum species linked to limited genomics here. The field of 'museomics' is moving fast and genomics is nowadays part of this. There are currently methods in development to use diverse type material, also for 'HMW' based extractions and often longer reads can be obtained (despite limitations exist, as stated by the reviewer). One development is to go to hybrid assemblies with a chromosome level genome of an extant sister or related species. However, this topic would go too far and could not be addressed in one sentence, despite it is indeed very interesting, see e.g. Raxworthy & Smith 2021, https://doi.org/10.1016/j.tree.2021.07.009 or the current research topic in frontiers: Recent advances in Museomics: Revolutionizing biodiversity research: https://www.frontiersin.org/research-topics/25109/recent-advances-in-museomics-revolutionizing-biodiversity-research#articles

11.) p 25, ll 554-556 – We added one reference for molluscs and a CTAB based protocol for invertebrates, however, this is indeed kind of 'common knowledge' gained by practical experience for which not much literature exists.

12.) p29, Figure 4 – The textbox is reduced, it can't be taken away because then the red x on grey mollusc cannot be seen well (contrast problem).

13.) p37, l 814 – Yes, thanks for the correction, it should be 9.2 Critical and future aspects on current bioassays. The mis-numbering is corrected.

14.) p 39 – l 848 – thanks for the comment, indeed the headers were inconsistent. We corrected that.

15.) p 44 l 967 – The citation format was corrected.

16.) p 44, l. 976 – We included the suggested reference (Yu et al 2021)

Close
