# Supplementary material for: Modern venomics—Current insights, novel methods, and future perspectives in biological and applied animal venom research
Source: Gigascience. 2022 May 18;11:giac048. doi: 10.1093/gigascience/giac048 (PMC9155608; doi:10.1093/gigascience/giac048)
Supplement: giac048_Reviewer_1_Report_Original_Submission — Mark J. Margres -- 2/17/2022 Reviewed [file giac048_reviewer_1_report_original_submission.pdf]

Reviewer Report

Title: Modern venomics â€“ Current insights, novel methods and future perspectives in biological and applied animal venom research

Version: Original Submission **Date:** 2/17/2022

Reviewer name: Mark J. Margres

Reviewer Comments to Author:

This review aims to synthesize the new and diverse evolutionary and more applied approaches used to characterizing venom diversity across a suite of venomous organisms. Overall, I like the structure of the review, from sample collection to data generation, although I did find the specifics on analysis in some sections lacking (e.g., genome assembly). I also found it strange that the review went from protein to RNA to morphology (section 6) rather than first to DNA/genomics (section 7). I think the flow would be improved if the central dogma outline was retained with morphology being section 7 (and genomics 6), but that is merely a suggestion. I also generally like the figures; I think they are well done. I outline a few major and minor concerns below, but the largest concern is that the authors exclude several recent, high-profile and relevant papers in their discussion of different techniques and questions. This can easily be addressed, but their exclusion was puzzling given their relevance to the particular sections.
MAJOR:
1. In section 2.1 the authors state how, until recently, most venomic studies have focused on snakes, spiders, and scorpions. I agree, but it would be useful to see a figure showing the number of published studies on various taxonomic groups to more convincingly demonstrate this point and show this knowledge gap. Over the past 10 years, for example, how many studies on snakes, scorpions, snails, bees, centipedes, etc.?
2. Figure 3 caption needs to be improved. Currently only a title sentence is supplied with no description of the figure itself.
3. The authors mention several new techniques but do not cite venomic studies using such techniques. For example, they mention ATAC-seq in section 5.2, but do not cite a recent venom study that used this approach:
Margres, M. J., Rautsaw, R. M., Strickland, J. L., Mason, A. J., Schramer, T. D., Hofmann, E. P., ... & Parkinson, C. L. (2021). The Tiger Rattlesnake genome reveals a complex genotype underlying a simple venom phenotype. Proceedings of the National Academy of Sciences, 118(4).
Similarly, in section 7, they cite a number of new snake, spider, and cone snail genomes but exclude the above citation as well as the other rattlesnake genome that was recently published:
Schield, Drew R., Daren C. Card, Nicole R. Hales, Blair W. Perry, Giulia M. Pasquesi, Heath Blackmon, Richard H. Adams et al. "The origins and evolution of chromosomes, dosage compensation, and mechanisms underlying venom regulation in snakes." Genome research 29, no. 4 (2019): 590-601.
I am less familiar with spiders and cone snails, but in reviews like this, these types of works certainly need to be included. The authors should make sure all recent, high-quality genomes are cited. Perhaps a table would be beneficial.

4. Similarly, the authors discuss venom gene origin in section 7.1 but ignore three papers from Sean Carroll's group that are perhaps our best current examples of such work:
Dowell, N., Giorgianni, M., Kassner, V., Selegue, J., Sanchez, E., & Carroll, S. (2016). The deep origin and recent loss of venom toxin genes in rattlesnakes. Current Biology, 26(18), 2434-2445.
Dowell, N. L., Giorgianni, M. W., Griffin, S., Kassner, V. A., Selegue, J. E., Sanchez, E. E., & Carroll, S. B. (2018). Extremely divergent haplotypes in two toxin gene complexes encode alternative venom types within rattlesnake species. Current Biology, 28(7), 1016-1026.
Giorgianni, M. W., Dowell, N. L., Griffin, S., Kassner, V. A., Selegue, J. E., & Carroll, S. B. (2020). The origin and diversification of a novel protein family in venomous snakes. Proceedings of the National Academy of Sciences, 117(20), 10911-10920.
These papers certainly need to be included and discussed in this section.
5. Why are no evolutionary studies of venom function using in-vivo assays discussed on lines 753-760? The entire bioassay section ignores evolutionary implications, which, according to the introduction was half of the focus of the review, and much work has been done in this area. Further, although in vitro and ex vivo assays can provide more precise information regarding the interaction of specific tissues and toxins as the authors describe, they do not characterize the overall ecological interaction between predator and prey (regardless of who is venomous) that in vivo assays do. Each has their strengths and place, and this should be discussed.
MINOR:
Typo in Abstract lines 106-107: "to identify more efficiently" should be "to more efficiently identify", please fix.
Line 118: "ensuring the fitness" strikes me as inappropriate; perhaps "that it is a critical component of the fitness" instead?
Line 128: "make" should me "making"
Line 140: "challenge" should be "challenges"
Lines 416-417: "identify as well house-keeping genes" should be "identify house-keeping genes as well", and "guarantee the venom secretion" again seems inappropriate; perhaps "participate in venom production"?
Line 497: comma after period at end of line
Lines 753-754: Grammar - needs to be "posing", "being", and "often not being"
Within section 9.1, the subsections are not numbered. Are they supposed to be?
Typo section title line 899: Immunomdulation

Methods

Are the methods appropriate to the aims of the study, are they well described, and are necessary controls included? Choose an item.

Conclusions

Are the conclusions adequately supported by the data shown? Choose an item.

Reporting Standards

Does the manuscript adhere to the journal's guidelines on [minimum standards of reporting?]() Choose an item.

Choose an item.

Statistics

Are you able to assess all statistics in the manuscript, including the appropriateness of statistical tests used? Choose an item.

Quality of Written English

Please indicate the quality of language in the manuscript: Choose an item.

Declaration of Competing Interests

Please complete a declaration of competing interests, considering the following questions:

- Have you in the past five years received reimbursements, fees, funding, or salary from an organisation that may in any way gain or lose financially from the publication of this manuscript, either now or in the future?
- Do you hold any stocks or shares in an organisation that may in any way gain or lose financially from the publication of this manuscript, either now or in the future?
- Do you hold or are you currently applying for any patents relating to the content of the manuscript?
- Have you received reimbursements, fees, funding, or salary from an organization that holds or has applied for patents relating to the content of the manuscript?
- Do you have any other financial competing interests?
- Do you have any non-financial competing interests in relation to this paper?

If you can answer no to all of the above, write 'I declare that I have no competing interests' below. If your reply is yes to any, please give details below.

I declare that I have no competing interests.

I agree to the open peer review policy of the journal. I understand that my name will be included on my report to the authors and, if the manuscript is accepted for publication, my named report including any attachments I upload will be posted on the website along with the authors' responses. I agree for my report to be made available under an Open Access Creative Commons CC-BY license (http://creativecommons.org/licenses/by/4.0/). I understand that any comments which I do not wish to be included in my named report can be included as confidential comments to the editors, which will not be published.

Choose an item.

To further support our reviewers, we have joined with Publons, where you can gain additional credit to further highlight your hard work (see: https://publons.com/journal/530/gigascience). On publication of this paper, your review will be automatically added to Publons, you can then choose whether or not to claim your Publons credit. I understand this statement.

Yes Choose an item.
