# Supplementary material for: Modern venomics—Current insights, novel methods, and future perspectives in biological and applied animal venom research
Source: Gigascience. 2022 May 18;11:giac048. doi: 10.1093/gigascience/giac048 (PMC9155608; doi:10.1093/gigascience/giac048)
Supplement: giac048_Reviewer_2_Report_Original_Submission — Matthew Holding -- 2/21/2022 Reviewed [file giac048_reviewer_2_report_original_submission.pdf]

Reviewer Report

Title: Modern venomics â€“ Current insights, novel methods and future perspectives in biological and applied animal venom research

Version: Original Submission **Date:** 2/21/2022

Reviewer name: Matthew Holding

Reviewer Comments to Author:

Review - Modern venomics - Current insights, novel methods and future perspectives in biological and applied animal venom research
The authors present a thorough review that spans the field to the bench to the bedside where venom research is concerned. The review covers new ground by mentioning the application of the latest in omic technologies and other new technological developments, while also serving as a 'review of reviews' as several aspects of venom biology and translational research have been extensively reviewed previously. They appropriately are scant on previously reviewed material while collating the pertinent facts and figures into a review that can serve as an up-to-date guide for anyone getting into venomics in the 2020s and standing on more than a century of work. I provide comments below on clarity and content improvement for several sections of the manuscript. Overall, a really nice piece of work that will help solidify and move the field.
Major:
Line 97-98: It will be more impactful to justify/explain exactly why the combination of "the evolutionary and the applied perspective" will be interesting or useful in the future, rather than just state that you are doing it.
Line 127-128: There are still comparatively few studies supporting a clear role for coevolution (defined as reciprocal selection between predator and prey and minimally suggested by local or species-level adaptation of venom or venom resistance) and these are not cited here even. So, this statement is a vast overstatement. I think coevolution plays a role in many (maybe most) venoms, but the way you state this here implies a large body of support that does not exist. Build the statement better with citations and context. An alternative to coevolution is that venom evolves to attack physiological targets and then prey, because maybe they have large R selected populations, do not evolve resistance due to trade-offs.
Line 164-173: You take too long to get to the important point here, and make the point very obliquely. State your thesis at the top of the paragraph: "An understanding of venomous animal diversity is crucial to understanding venomous animal diversity and novel protein functions. Yet, taxonomic expertise in many venomous animal groups has been on the decline".
Line 184 to 188: This is too important of a point set to include in a single long sentence. Break up proposed benefits and costs of Nagoya into two sentences. Doing so will let you expand more specifically on what you mean by hinderance of "translational applications". Do you mean the speed with which they can be discovered and enacted at scale? Additionally, you present these points without really taking a stance, even a nuanced one. For example, given that the WHO wants to half snakebite by 2030, if Nagoya protocol is perceived to slow progress there, then two international agreements/bodies

are in potential conflict. Right now this paragraph merely raises in the most vague terms some benefits and costs of the very important Nagoya protocol, and so leaves the reader to draw whatever conclusions they want to without additional context that could easily be made available by the authors. I believe this is the first time I have seen Nagoya raised in a venom paper, so the treatment needs to be thorough, thoughtful, and nuanced or not at all.

Line 222 - 257: It would be helpful to readers new to metabolomics to expand a bit on two things: First is to methodologically differentiate metabolomics from the better-known proteomics. What specific molecule types can be detected and how? Second, can you talk in more physiologic detail about at least one of the toxins discovered through metabolomics? For example, you mention the acylpolyamines in line 240 and then several other molecules in lnes 248-250. Were any of these subsequently tested in vivo in mice or insect models and show to be toxic? If show, recitation of those specific details in brief would benefit this section in terms of biological reality.

Lines 361-372: The authors are focusing heavily on the utility of RNA-seq data for making databases for proteomics. However, there is an entire subfield of venom biology that uses the RNAseq data alone to study differential expression of venom genes. The authors should highlight and discuss this as it is the most common use of venom rna-seq data.

Lines 455 - 545: This section on morphological analysis of venom production is generally well-written and informative. I think it currently lacks a broader justification for why this should be an interesting topic of investigation. The previous sections set up venom variation as important for various biological and translational reasons, but why is venom morphology/spatial analysis of expression interesting? I am intrinsically interested, but would ask the authors to expand more specifically on what they think we can learn by integrating morphology and venomics, beyond the very general explanations they give (e.g. lines 542-545). What hypotheses exist and what can be tested? Can we discover new venom functions or new venom proteins based on spatial analyses? What else? Without this information this otherwise well-done and interesting section sticks out as an "add-on" to the rest of the paper.

Lines 819-826: most readers will likely have only heard of these methods, if that. It would be helpful to the reader to give a very brief idea of how these various methods might be useful in venom biology. Otherwise, it is just a list of methods that could equally be substituted for any other. You could summarize in a table that has columns of "method" and "proposed use in venom research", or just write it out. But more detail required or delete the section entirely.

References cited: Likely due to my own reading biases, a disproportionate citation of venom research from the Eastern hemisphere is noted. The comprehensiveness of the review would benefit from more representative considerations of the work done given that the review addresses global challenges.

Length: I found the review to be a bit long. I think with shoring up of the writing with an eye for conciseness, the authors could reduce the length of the paper by 10-20%, while still being as informative but more impactful.

Minor:

Line 92: "taxasuch" needs a space. Many other combined words are noted throughout the paper and need to be search out.

Line 92-96: Run-on sentence can be split and simplified to increase impactfulness: "There are many outstanding questions on the evolution of toxin arsenals, such as how venom genes originate, how venom contributes to the fitness of venomous species, and which modifications at the genomic,

transcriptomic and protein level drive their evolution. These questions have received particularly little attention outside of snakes, spiders, and scorpions".
Line 97: Reword "translational research aspects", as it is not really clear what you mean.
Line 106-107: "more efficiently identify"
Line 107: What is an "adapted system". Seems like you mean in vitro, but can be confused with evolutionary adaptation.
Line 118: "such an effective adaptation" is a jump in logic. It might have evolved 100 times because it is easy (i.e. a small jump in the fitness landscape using exisiting genes and glands) rather than effective. It is okay to say venom is everywhere or ubiquitous in natural ecosystems or something similar.
Line 121: Again, I don't think there is evidence that venom ensures the stability of food webs. If you want to argue that, for example, ants, bees, jellyfish and other very common important species are important to ecosystem functioni, and therefore than venoms are involved at a basic level in community interactions, that makes more sense. But this is too straightforwardly stated without a corresponding citation or appropriate context.
Line 136-137: Written somewhat informally. How about: "The modern field of venomics integrates the evolution, ecology, and translational research on venoms".
Line 137-138: Sentence seems to be contrasting venomics with snakebite, but venomics certainly also concerns itself with better snakebite treatments. Overall, the sentence starting with "However" seems unnecessary and a bit flowery. I would remove it.
Line 140: "challenges"
Line 141: I think "Neozoic" is defining a broader time range than is desired here. Do you simply mean to state that "Additionally, climate change may facilitate movement of venomous species into new areas, posing risks to previously unexposed humans, native species, and livestock"? Honestly, I think the previous sentence would be a better end to this paragraph and this climate change sentence can be deleted.
Line 148: relace "all the way towards the" with "to". Then put a period after research, so that the last sentence just reads "We aim to create a blueprint for future venomics studies and a roadmap toward new methodological perspectives".
Line 153: "evolutionary trait" is a vague and somewhat useless phrase. I think you could just short to "the importance and impact of venom". It is already implied that it is a trait, and it evolves.
Line 218: What is meant by "pre-purified". This could be fractionation or just spinning out large cell debris, or something else. Explain further please.
Line 231: Delete "but gradually picking up".
Line 237: The mentioned 1936 work does not appear to be cited.
Line 264: Delete "nowadays".
Line 435: Authors should probably cite the single ATAC-seq containing venom paper:
https://www.pnas.org/content/118/4/e2014634118
Line 899: Header misspelled
Line 905 : "rattlesnake" is on word
Lines 911-918: Several citations are not numbered here.
Line 927: It is an oversell to say that they ARE the future. Tone down.
Line 944: MinION may require a trademark symbol?

Line 980: This sentence does not make sense. Something missing or in need of rewording.
Line 1003: "excising"
Figures:
Figure 1. Beautiful figure overall! I would argue that regulatory evolution is a major basic research component that you are missing hre.
Figure 2. The fading color on the connecting tracks make it more difficult to discern the connections being drawn. Use solid color throughout.
Best,
Matt Holding

Methods

Are the methods appropriate to the aims of the study, are they well described, and are necessary controls included? Choose an item.

Conclusions

Are the conclusions adequately supported by the data shown? Choose an item.

Reporting Standards

Does the manuscript adhere to the journal's guidelines on minimum standards of reporting? Choose an item.

Choose an item.

Statistics

Are you able to assess all statistics in the manuscript, including the appropriateness of statistical tests used? Choose an item.

Quality of Written English

Please indicate the quality of language in the manuscript: Choose an item.

Declaration of Competing Interests

Please complete a declaration of competing interests, considering the following questions:

- Have you in the past five years received reimbursements, fees, funding, or salary from an organisation that may in any way gain or lose financially from the publication of this manuscript, either now or in the future?
- Do you hold any stocks or shares in an organisation that may in any way gain or lose financially from the publication of this manuscript, either now or in the future?

- Do you hold or are you currently applying for any patents relating to the content of the manuscript?
- Have you received reimbursements, fees, funding, or salary from an organization that holds or has applied for patents relating to the content of the manuscript?
- Do you have any other financial competing interests?
- Do you have any non-financial competing interests in relation to this paper?

If you can answer no to all of the above, write 'I declare that I have no competing interests' below. If your reply is yes to any, please give details below.

I declare that I have no competing interests

I agree to the open peer review policy of the journal. I understand that my name will be included on my report to the authors and, if the manuscript is accepted for publication, my named report including any attachments I upload will be posted on the website along with the authors' responses. I agree for my report to be made available under an Open Access Creative Commons CC-BY license (http://creativecommons.org/licenses/by/4.0/). I understand that any comments which I do not wish to be included in my named report can be included as confidential comments to the editors, which will not be published.

Choose an item.

To further support our reviewers, we have joined with Publons, where you can gain additional credit to further highlight your hard work (see: https://publons.com/journal/530/gigascience). On publication of this paper, your review will be automatically added to Publons, you can then choose whether or not to claim your Publons credit. I understand this statement.

Yes Choose an item.
