# Supplementary material for: Modern venomics—Current insights, novel methods, and future perspectives in biological and applied animal venom research
Source: Gigascience. 2022 May 18;11:giac048. doi: 10.1093/gigascience/giac048 (PMC9155608; doi:10.1093/gigascience/giac048)
Supplement: giac048_Reviewer_3_Report_Original_Submission — Jason Macrander, Ph. D. -- 3/8/2022 Reviewed [file giac048_reviewer_3_report_original_submission.pdf]

Reviewer Report

Title: Modern venomics â€“ Current insights, novel methods and future perspectives in biological and applied animal venom research

Version: Original Submission **Date:** 3/8/2022

Reviewer name: Jason Macrander, Ph. D.

Reviewer Comments to Author:

The authors provided a very extensive and thorough review of modern venomics, focusing on current approaches and potential applications. The authors provided a robust framework to guide the readers through work that has been done in this area with broad taxonomic representation. This review brings attention to areas of modern venomics that have been largely ignored, for example the importance of taxonomic expertise, the use of metabolomics, and limitations to widely accepted comparative venomic methods. The authors also emphasize the drawbacks the field faces, such as the detriment of clinical applications of treating envenomation in areas where it is badly needed, the importance of data sharing and collection permits, and the roadblocks we currently face with high throughput functional annotation.

The manuscript is well written (although I have some very minor comments in the attached manuscript draft) and would be of broad interest to the GigaScience readers, as well as bring some venom researchers to the GigaScience journal that I feel has a lot the field of comparative venomics with big data and analysis. In the attached pdf I have some minor comments with regards to editing, and suggest the authors review the journal preference for the use (or disuse) of the Oxford comma as it seems to be used inconsistently. Although there is a large number of venom review papers out in the current literature, I feel that this review is a substantial and necessary addition to the field of modern comparative venomics. It will be a positive and significant contribution to the field of modern venomics.

Methods

Are the methods appropriate to the aims of the study, are they well described, and are necessary controls included? Choose an item.

Conclusions

Are the conclusions adequately supported by the data shown? Choose an item.

Reporting Standards

Does the manuscript adhere to the journal's guidelines on [minimum standards of reporting?]() Choose an item.

Choose an item.

Statistics

Are you able to assess all statistics in the manuscript, including the appropriateness of statistical tests used? Choose an item.

Quality of Written English

Please indicate the quality of language in the manuscript: Choose an item.

Declaration of Competing Interests

Please complete a declaration of competing interests, considering the following questions:

- Have you in the past five years received reimbursements, fees, funding, or salary from an organisation that may in any way gain or lose financially from the publication of this manuscript, either now or in the future?
- Do you hold any stocks or shares in an organisation that may in any way gain or lose financially from the publication of this manuscript, either now or in the future?
- Do you hold or are you currently applying for any patents relating to the content of the manuscript?
- Have you received reimbursements, fees, funding, or salary from an organization that holds or has applied for patents relating to the content of the manuscript?
- Do you have any other financial competing interests?
- Do you have any non-financial competing interests in relation to this paper?

If you can answer no to all of the above, write 'I declare that I have no competing interests' below. If your reply is yes to any, please give details below.

I declare that I have no competing interests

I agree to the open peer review policy of the journal. I understand that my name will be included on my report to the authors and, if the manuscript is accepted for publication, my named report including any attachments I upload will be posted on the website along with the authors' responses. I agree for my report to be made available under an Open Access Creative Commons CC-BY license (http://creativecommons.org/licenses/by/4.0/). I understand that any comments which I do not wish to be included in my named report can be included as confidential comments to the editors, which will not be published.

Choose an item.

To further support our reviewers, we have joined with Publons, where you can gain additional credit to further highlight your hard work (see: https://publons.com/journal/530/gigascience). On publication of this paper, your review will be automatically added to Publons, you can then choose whether or not to claim your Publons credit. I understand this statement.

Yes Choose an item.
